# Re and ^99m^Tc complexes of BodP_3_ – multi-modality imaging probes^†^

**DOI:** 10.1039/c4cc06367h

**Published:** 2014-09-24

**Authors:** Laura H. Davies, Benjamin B. Kasten, Paul D. Benny, Rory L. Arrowsmith, Haobo Ge, Sofia I. Pascu, Stan W. Botchway, William Clegg, Ross W. Harrington, Lee J. Higham

**Affiliations:** a School of Chemistry, Newcastle University, Bedson Building, Newcastle upon Tyne, NE1 7RU, UK; b Department of Chemistry, Washington State University, Pullman, WA 99164, USA; c Chemistry Department, University of Bath, Bath, BA2 7AY, UK; d Central Laser Facility, STFC, Rutherford Appleton Laboratory, Harwell Science and Innovation Campus, Oxfordshire, OX11 0QX, UK

## Abstract

A fluorescent tridentate phosphine, BodP_3_ (2), forms rhenium complexes which effectively image cancer cells. Related technetium analogues are also readily prepared and have potential as dual SPECT/fluorescent biological probes.

^99m^Tc–phosphine complexes are used in approved and emerging Single-Photon Emission Computed Tomography (SPECT) imaging agents due to the attractive nuclear properties of ^99m^Tc (*γ* = 140 keV, *t*_1/2_ = 6 h) and to the facile formation of inert Tc–P coordinate bonds.^1^ Resolving the cellular fate of such radiopharmaceuticals remains challenging due to the spatial limitations of SPECT, thus there is a drive to develop novel probes with fluorescent tags in order to facilitate high-resolution imaging by fluorescence microscopy. ^2^ Such a species has to be (i) kinetically inert, (ii) highly fluorescent upon metal ligation, and (iii) resistant to degradation by biological molecules. Here we report our work on developing phosphorus-based probes for this and related applications, including therapeutics. We recently described the synthesis of tridentate phosphine **2**, BodP_3_, (Scheme 1) from air-stable **1**, with both retaining the attractive photophysical properties common to Bodipy.^3^ Bianchini reported the synthesis of the single isomer *cis, fac*-[ReCl(CO)_2_(triphos-Me)] by refluxing *mer*-[ReCl(CO)_3_(PPh_3_)_2_] with 1,1,1-tris(diphenylphosphinomethyl) ethane.^4^ With this in mind, **2** was reacted with *mer*-[ReCl(CO)_3_(PPh_3_)_2_] under similar conditions; however, a mixture of three stereoisomers was generated, **3a–c**.

These were separated *via* chromatography and all three were then characterised by X-ray crystallography (Fig. 1 and ESI^†^).

Bis(diphenylphosphinoethyl)phenylphosphine (triphos-Ph) reacts with [ReCl(CO)_5_] in refluxing toluene to form the related isomers reported here, but on prolonged reaction times (> 24 h) at 170 °C, only the *cis,mer*(1) isomer was produced.^5^ Phosphine **2** was therefore reacted with *mer*-[ReCl(CO)_3_(PPh_3_)_2_] in refluxing mesitylene and after 4 h, ^31^P{^1^H} NMR spectroscopy indeed showed only **3a**. In comparison, the radioactive synthon *fac*-[^99m^Tc(CO)_3_(OH_2_)_3_]^+^ is readily prepared *in situ* from an IsoLinks^®^ kit.^6^ Substitution of the aqua ligands under aqueous or mixed polar organic conditions readily occurs, requiring the preparation of rhenium analogues under more polar conditions, to better correlate with the ^99m^Tc experiments. Thus triphos-Ph was reacted with [Re(CO)_5_][OTf] in refluxing ethanol to give *fac*-[Re(CO)_3_(triphos-Ph)][OTf], **4**; a sample suitable for X-ray crystallographic analysis was obtained by pentane diffusion into a tetrahydrofuran solution (Fig. 2). To synthesise a fluorescent analogue, **2** was reacted with [Re(CO)_5_][OTf] to give exclusively *fac*-[Re(CO)_3_(**2**)][OTf], **5**, characterised by X-ray diffraction (Fig. 3). The Re–P and Re–C bond lengths and angles are typical for such complexes.^4,5^ The photophysical properties of **2, 3a–c** and **5** were then measured (Table 1). The absorption spectra of **2** and the complexes showed a strong S_0_–S_1_ (*π–π*^⋆^) transition with a maximum of 512 or 513 nm, assigned to the Bodipy core.^7^

Compound **2** has a typically high molar absorption coefficient of 90 000 M^−1^ cm^−1^ which is lowered for the associated metal complexes (60 000–64 000 M^−1^ cm^−1^). A lower-intensity, broader absorption band between 370 and 380 nm (*ε* = 2500–4200 M^−1^ cm^−1^) is attributed to the S_0_–S_2_ (*π–π*^⋆^) transition of the Bodipy core;^7^ the absorption profiles are displayed in Fig. 4. Phosphine **2** and its complexes all exhibit emission at room temperature in tetrahydrofuran, dichloromethane andmethanol, on excitation at 485 nm. The emission maximum (*λ*_em_) is seen at 527 nmin tetrahydrofuran for **2**, and is shifted to lower wavelengths in dichloromethane and methanol. Upon complexation, no or very little change is observed in the emission maxima. For all the complexes, when the solvent is changed from dichloromethane to methanol, the emission maxima are slightly blue-shifted. The Stokes shift for the complexes are small (14–15 nm in THF), suggesting negligible structural change on excitation. The fluorescence quantum yield (*Φ*_F_) for **2** is 0.34 in tetrahydrofuran, which is comparable to the parent primary phosphine **1** (*Φ*_F_ = 0.33).^3^ This is important, as it shows that the two additional phosphorus groups in this tridentate derivative do not impact negatively on the fluorescence, and indicates reductive-PeT is not occurring.

On coordination, the fluorescence quantum yields are slightly lowered for all the complexes (Table 1). One explanation for this may be the heavy atom effect, causing spin–orbit coupling and giving rise to intersystem crossing to the triplet state.^8^

The *Φ*_F_ values are high in comparison to tridentate quinolinederived nitrogen-based rhenium complexes, which have *Φ*_F_ of 0.003–0.015, and therefore these phosphorus-based probes may be even more sensitive *in vitro* cell imaging agents.^2^

The corresponding technetium complexes were first investigated using triphos-Ph as a mimic for **2**, to establish the general labelling conditions. Reacting *fac*-[^99m^Tc(CO)_3_(OH_2_)_3_]^+^ with 1 × 10^−5^ M triphos-Ph in a pH 7.2 sodium phosphate buffer–ethanol solution at 85 °C for 1 h resulted in quantitative radiochemical conversion to **6**. A single HPLC peak for **6** closely matched that of *fac*-[Re(CO)_3_(triphos-Ph)]^+^ **4** (Scheme 2 and Fig. 5). Under the same conditions, the Bodipy phosphine, **2**, produced *fac*-[^99m^Tc(CO)_3_(**2**)]^+^ **7** in similar conversion, giving a single major HPLC peak that correlated with the rhenium analogue **5** (Fig. 5). The stabilities of **6** and **7** were examined by radio-HPLC using competitive amino acid challenge assays (1 mM histidine or cysteine) to simulate an *in vivo* environment ([PO_4_]^3−^ 10 mM, pH 7.2, 37 °C). Analysis by HPLC indicated that both complexes remained >97% stable up to 18 h; no *trans*-chelation of the *fac*-[^99m^Tc(CO)_3_]^+^ core in **6** or **7** with either amino acid was observed during the study. The radiolabelling yields, purity and stability of **6** and **7** indicate that the tridentate phosphine ligand system is comparable for *fac*-[^99m^Tc(CO)_3_]^+^ to other potent tridentate chelates (*e.g*. histidine, bis(2-pyridylmethyl)-amine [DPA]).^9^ Therefore, to the best of our knowledge, complex **7** represents the first example of a phosphine-based, multi-functional imaging tool, combining (i) a tridentate phosphine for kinetic stability, (ii) a fluorophore for *in vitro* imaging, and (iii) a radioactive metal for *in vivo* imaging *via γ*-detection by SPECT.

Radiolabelling of the complexes constitutes the first, crucial step towards their use in nuclear medicine for *in vivo* tissue imaging, but SPECT does not provide information at the subcellular level due to resolution limitations (1–2 mm). In contrast, optical imaging methods allow for the direct visualisation of the uptake and localisation of complexes within cells which often contributes to the understanding of the mechanism of action of such probes in cellular environments, due to the sub-micron resolution level.^10^ Thus, in a preliminary screening, the rhenium complexes **3a** and **5** were imaged in prostate carcinoma (PC-3) cells (cultured as described in the ESI^†^), by epi-fluorescence microscopy using single-photon excitation between 460 and 500 nm and an emission filtered at 510 nm (Fig. 6). Remarkably, exchanging a chloride for a carbonyl ligand renders the cellular behaviour of **3a** and **5** very different; whereas [ReCl(CO)_2_(**2**)]^+^ allows for high-resolution imaging and enables visualisation of organelles without any apparent cytotoxicity, *fac*-[Re(CO)_3_(**2**)]^+^, **5**, on the other hand causes some morphological changes. Further investigations would elucidate the sub-cellular localisation of the rhenium complexes into specific organelles. MTT assays were carried out in PC-3 cells, which indicated an MI_50_ (the concentration required to reduce mitochondrial metabolism to 50%) of 45 μM ± 5 μM for complex **5**, confirming its cytotoxic effect (ESI^†^). Furthermore, MTT assays indicated that complex **3a** was innocuous up to 250 μM after 48 h incubation (ESI^†^). Moreover, both **3a** and **5** possess negligible cytotoxicity at the concentrations required for detection *via* SPECT, confirming that they are highly appropriate for use as imaging probes,^11^ and our work is now focused in this direction.

We thank Dr Dyszlewski at Covidien for providing the Isolink^®^ kits, the NIH/NIGMS (Institutional Award T32-GM008336) and EPSRC for funding (EP/G005206/1) and the NMSSC, Swansea for Mass Spectra. SIP and SWB thank the Royal Society, MRC and STFC for support. We also thank Prof. Jon Dilworth for rhenium salts and advice.

## Supplementary Material

Supplemental

## Figures and Tables

**Fig 1:**
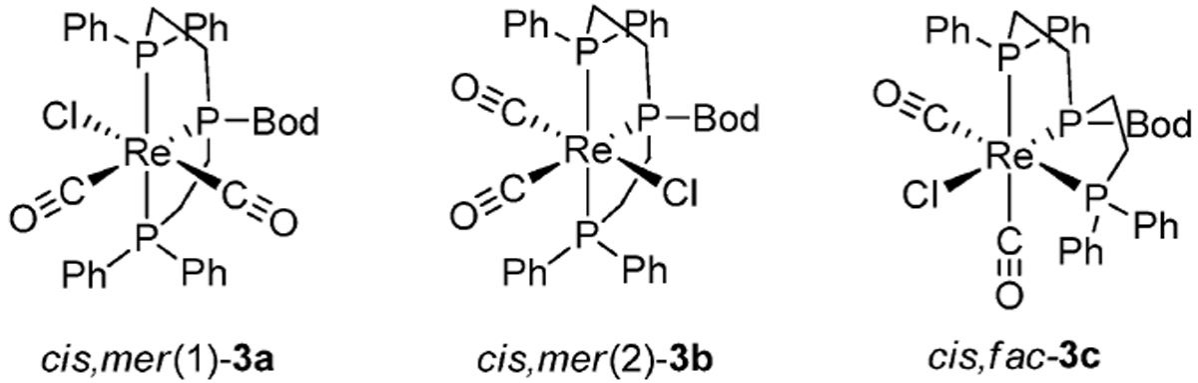
The three isomers isolated from the reaction of the tridentate phosphine **2** and [ReCl(CO)_3_(PPh_3_)_2_] in refluxing toluene.

**Fig 2:**
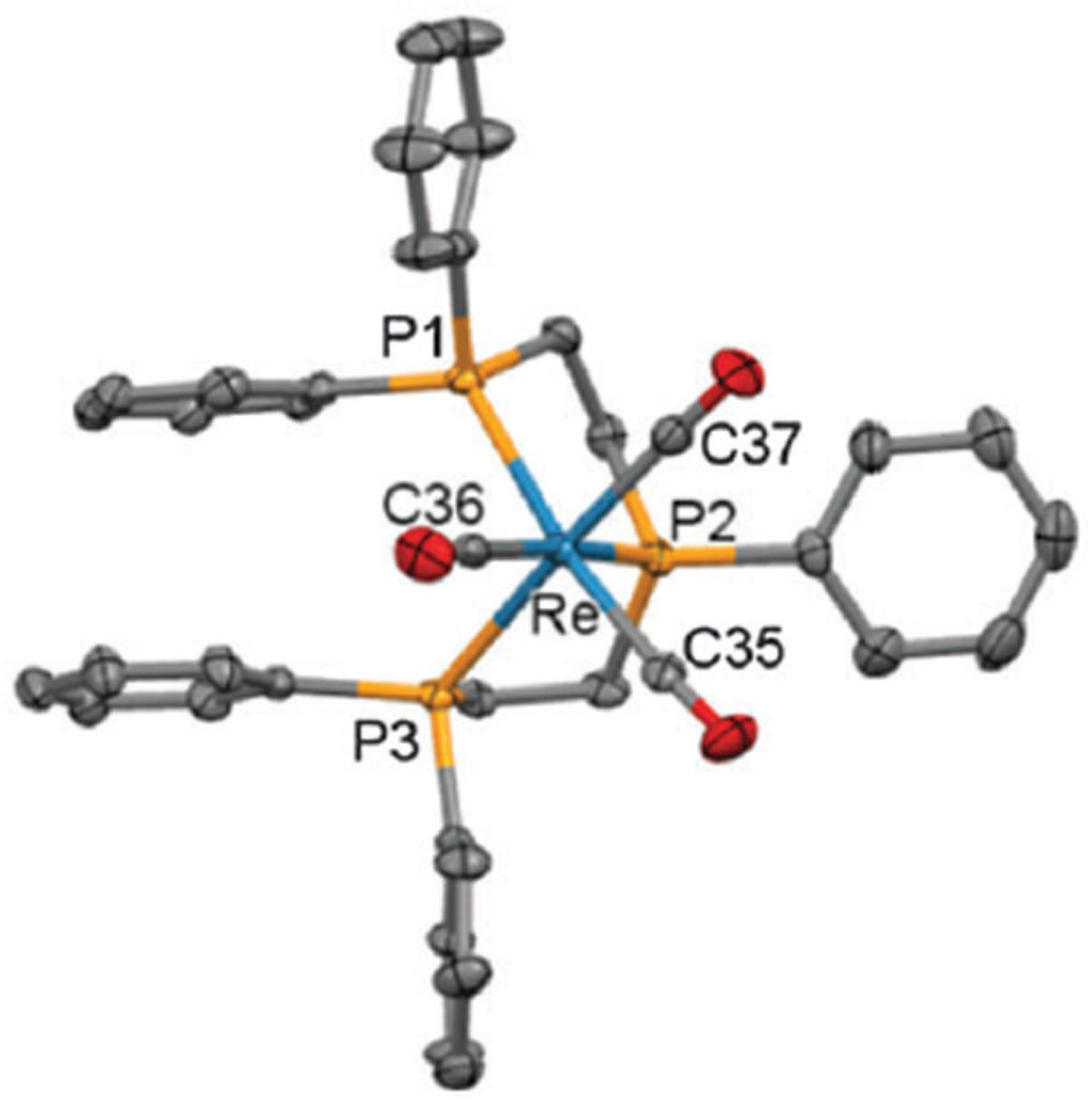
X-ray crystallographic structure of **4** with 50% probability displacement ellipsoids. Hydrogen atoms and the anion are omitted for clarity. Selected bond distances [Å] and angles [°]: Re–P1 2.4680(12), Re–P2 2.4313(13), Re–P3 2.4666(12), Re–C35 1.957(5), Re–C36 1.971(6), Re–C37 1.954(6); P1–Re–P2 81.88(4), P1–Re–P3 95.12(4), P2–Re–P3 80.92(4), P1–Re–C35 169.49(16), P2–Re–C36 173.86(14), P3–Re–C37 174.70(16).

**Fig 3:**
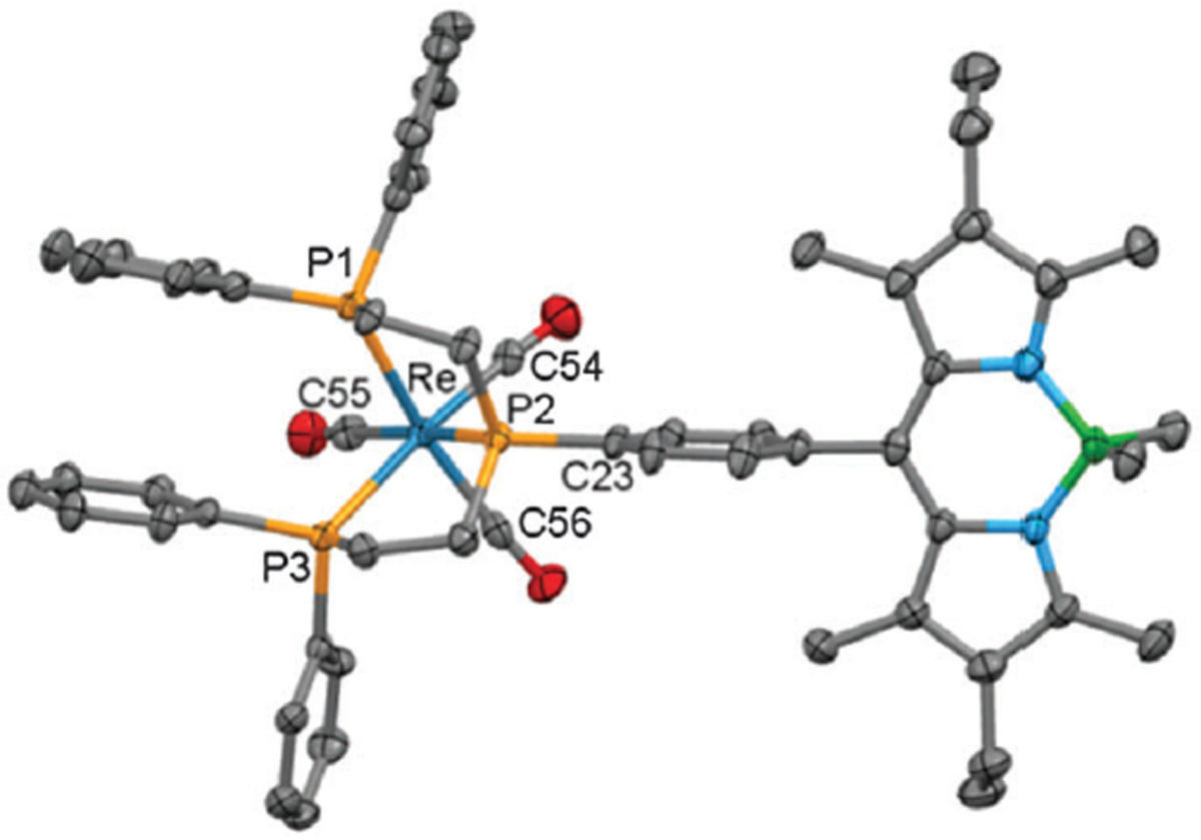
X-ray structure of **5**. Hydrogen atoms, the anion and solvent molecules are omitted for clarity. Selected bond distances [Å] and angles [°]: Re–P1 2.4544(13), Re–P2 2.4230(13), Re–P3 2.4831(13), Re–C54 1.926(6), Re–C55 1.975(6), Re–C56 1.944(6); P1–Re–P2 81.44(5), P1–Re–P3 91.64(4), P2–Re–P3 81.11(4), P1–Re–C56 167.69(18), P2–Re–C55 173.27(17), P3–Re–C54 174.80(17).

**Fig 4:**
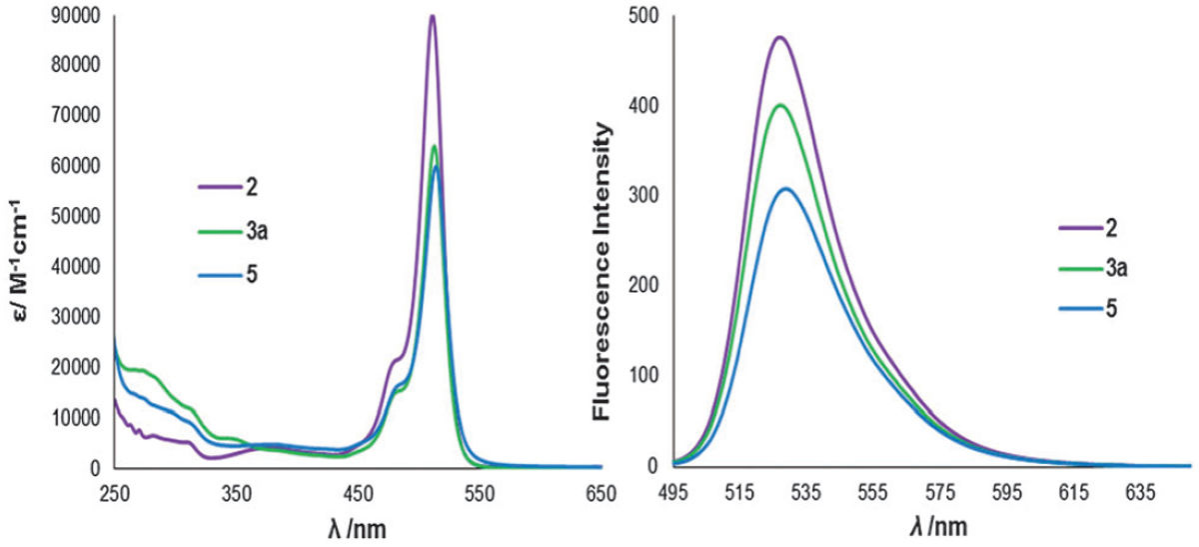
Absorption and emission spectra of **2** and its metal complexes *cis,mer*-[ReCl(CO)_2_(**2**)][OTf] **3a** and *fac*-[Re(CO)_3_(**2**)][OTf] **5** in THF. For **3b** and **3c** see ESI.^†^

**Fig 5:**
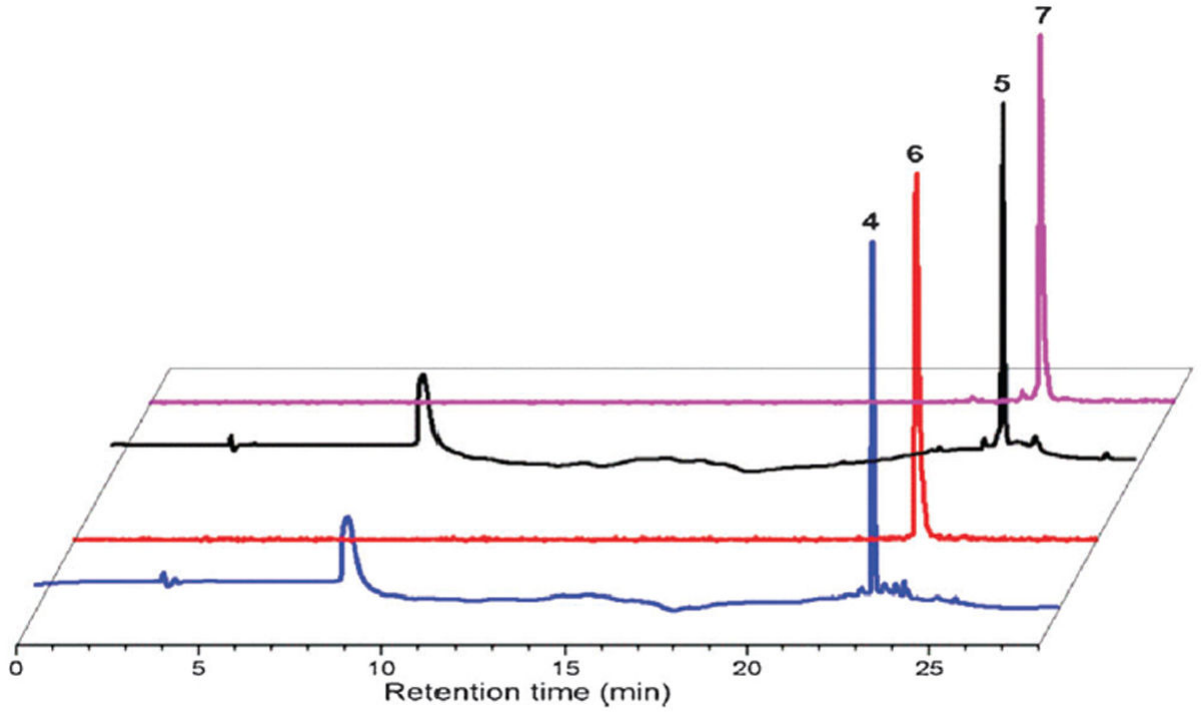
Overlay of HPLC chromatograms from the crude reactions between triphos-Ph or **2** and [Re(CO)_5_]^+^ (UV, 220 nm) or *fac*-[^99m^Tc(CO)_3_(OH_2_)_3_]^+^(NaI, *γ*-detector) to yield *fac*-[Re(CO)_3_(triphos-Ph)]^+^ (**4**) at 22.9 min, *fac*-[^99m^Tc(CO)_3_(triphos-Ph)]^+^ (**6**) at 23.0 min, *fac*-[Re(CO)_3_(**2**)]^+^ (**5**) at 24.4 min and *fac*-[^99m^Tc(CO)_3_(**2**)]^+^ (**7**) at 24.5 min.

**Fig 6:**
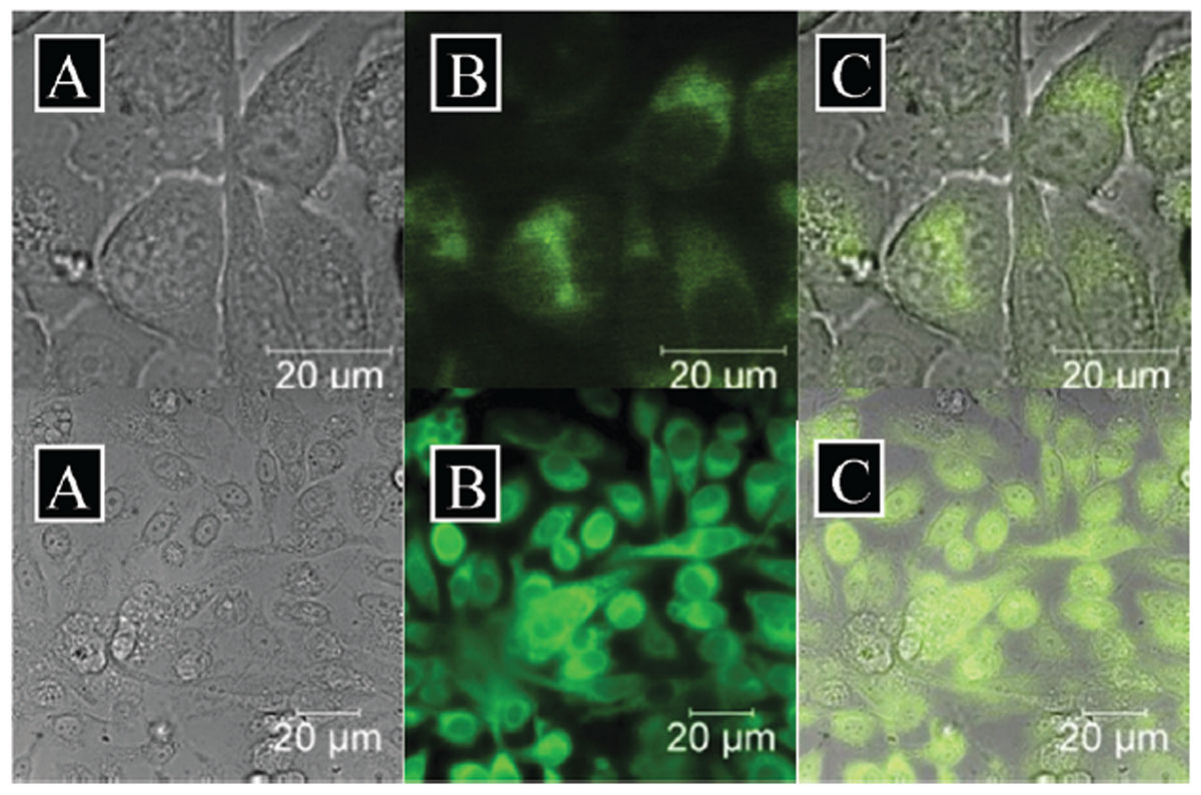
Epi-fluorescence imaging of PC-3 living cells with compounds **3a** and **5**. Top: *cis,mer*-[ReCl(CO)_2_(**2**)], **3a**, 100 μM, 1% DMSO, 15 minutes. Bottom: *fac*-[Re(CO)_3_(**2**)]^+^, **5**, 50 μM, 2% ethanol, 15 minutes. (A) Brightfield image, (B) green channel *λ*_ex_ = 460–500 nm, long pass filtered at 510 nm, (C) overlay of A and B.

**Scheme 1:**
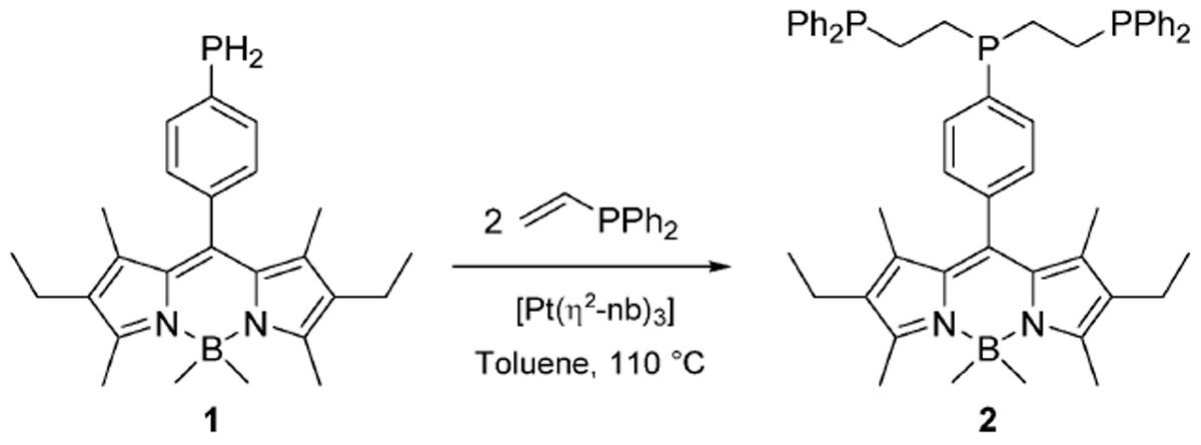
The hydrophosphination reaction between primary phosphine **1** and vinyldiphenylphosphine, to produce the tridentate derivative **2**, BodP_3_.

**Scheme 2:**
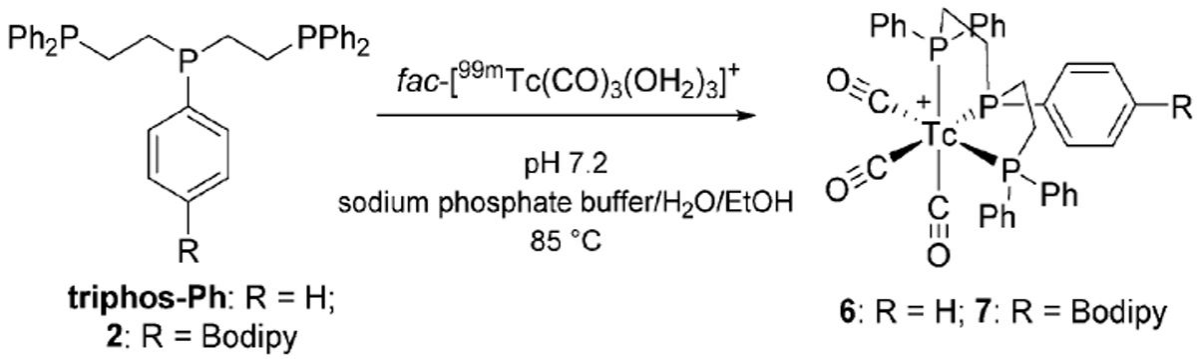
Synthesis of *fac*-[^99m^Tc(CO)_3_(triphos-Ph)]^+^ **6** and *fac*-[^99m^Tc(CO)_3_(**2**)]^+^ **7**.

**Table 1:** Photophysical data for phosphine **2** and its Re complexes

	*λ*_abs_ a/nm	*ε*^a^/M^−1^ cm^−1^	*λ*_em_ a/nm	*Φ*_F_ a,b	*λ*_em_ c/nm	*Φ*_F_ b,c	*λ*_em_ d/nm	*Φ*_F_ b,d
**2**	513	90 000	527	0.34	525	0.40	523	0.39
**3a**	513	64 000	527	0.28	529	0.26	526	0.27
**3b**	513	63 000	527	0.26	528	0.26	525	0.27
**3c**	512	—e	526	0.18	527	0.24	524	0.25
**5**	513	60 000	528	0.24	531	0.18	527	0.20

a In degassed tetrahydrofuran at room temperature.

b Measured with respect to 4,4-difluoro-8-phenyl-1,3,5,7-tetramethyl-2,6-diethyl-4-bora-3*a*,4*a*-diaza-*s*-indacene; dyes were excited at 485 nm.

c In degassed dichloromethane at room temperature.

d In degassed methanol at room temperature.

e Insufficient quantity of sample isolated for measurement.

## Footnotes

† Electronic supplementary information (ESI) available: Spectroscopic, crystallographic and photophysical data, cell imaging and cytotoxicity experiments. CCDC 849753 and 1001449–1001452. For ESI and crystallographic data in CIF or other electronic format see DOI: 10.1039/c4cc06367h
