# Supplementary material for: Re and 99mTc complexes of BodP3 – multi-modality imaging probes
Source: Chem Commun (Camb). Author manuscript; Available in PMC 2015 Dec 21. (PMC4659712; doi:10.1039/c4cc06367h)
Supplement: Supplemental [file NIHMS736898-supplement-Supplemental.pdf]

Electronic Supplementary Material (ESI) for Chemical Communications.
This journal is © The Royal Society of Chemistry 2014

Supporting Information

Table of Contents

Table of Contents 1

1 General Considerations and Chemical Analysis 2

2 Experimental Procedures 3

2.1 *cis*,*mer*(1)-[ReCl(CO)$_2$(2)](3a) 3

2.2 *cis,mer*(2)-[ReCl(CO)$_2$(2)] (3b) 4

2.3 *cis,fac*-[ReCl(CO)$_2$(2)] (3c) 5

2.4 *fac*-[Re(CO)$_3$(triphos-Ph)][OTf] (4) 5

2.5 *fac*-[Re(CO)$_3$(2)][OTf] (5) 7

3 Radiochemistry 9

3.1 General Experimental Considerations 9

3.2 *fac*-[^{99m}Tc(CO)$_3$(triphos-Ph)]$^+$ (6) 9

3.3 *fac*-[^{99m}Tc(CO)$_3$(2)]$^+$ (7) 10

3.4 Stability Analysis 11

4 Cell Imaging 13

4.1 Cell Culturing and Cell Preparation 13

4.2 MTT Assays 13

4.3 Fluorescence Microscopy 14

5 X-ray Crystallography 16

5.1 General Information 16

5.2 Crystallographic data for *cis*,*mer*(2)-[ReCl(CO)$_2$(2)](3b) 17

5.3 Crystallographic data for *cis*,*fac*-[ReCl(CO)$_2$(2)](3c) 37

5.4 Crystallographic data for *fac*-[Re(CO)$_3$(triphos-Ph)][OTf] (4) 75

5.5 Crystallographic data for *fac*-[Re(CO)$_3$(2)][OTf] (5) 91

6 Absorption and Emission Spectroscopy 116

6.1 General Considerations 116

6.2 Absorption and Emission Spectra 116

6.3 Quantum Yield Method 117

7 References 119

1 General Considerations and Chemical Analysis

All air- and/or water-sensitive reactions were performed under a nitrogen atmosphere using standard Schlenk line techniques. Tetrahydrofuran was dried over sodium/benzophenone, toluene and mesitylene dried over sodium and ethanol over magnesium ethoxide; all solvents were distilled prior to use. Pentane was purchased in an anhydrous state. All starting materials were purchased from Aldrich, Acros Organics, Alfa Aesar, Strem or Johnson Matthey and used as received. Reference 4,4-difluoro-8-phenyl-1,3,5,7-tetramethyl-2,6-diethyl-4-bora-3a,4a-diaza-s-indacene,[1] $[Pt(norbornene)_3]$,[2] *mer*-$[ReCl(CO)_3(PPh_3)_2]$,[3] $[ReBr(CO)_3$(3,4-diaminobenzoic acid)],[4] and $[Re(CO)_5][OTf]$[5] were prepared according to literature procedures. The experimental procedure and characterisation data for **2** and detailed crystallographic data for **3a** are given in our initial report.[6]

Flash chromatography was performed on silica gel from Fluorochem (silica gel, 40-63u, 60A, LC301). Thin-layer chromatography was performed on Fisher aluminium-based plates with silica gel and fluorescent indicator (254 nm). Melting points were determined in open glass capillary tubes on a Stuart SMP3 melting point apparatus. ^{1}H, $^{13}C\{^{1}H\}$, $^{31}P\{^{1}H\}$, and $^{11}B\{^{1}H\}$ NMR spectra were recorded on a JEOL Lambda 500 (^{1}H 500.16 MHz) or JEOL ECS-400 (^{1}H 399.78 MHz) spectrometer at room temperature (21°C) using the indicated solvent as internal reference; ^{1}H and ^{13}C shifts were relative to tetramethylsilane, ^{31}P relative to 80% H_3PO_4, and ^{11}B relative to $BF_3.Et_2O$. Infrared spectra were recorded on a Varian 800 FT-IR spectrometer and mass spectrometry was carried out by the EPSRC National Mass Spectrometry Service Centre, Swansea.

2 Experimental Procedures

2.1 *cis,mer*(1)-[ReCl(CO)$_2$(2)](3a)

8-(4-(Bis-2-ethyldiphenylphosphino)-phenylphosphino)-4,4-dimethyl-1,3,5,7-tetramethyl-2,6-diethyl-4-bora-3a,4a-diaza-*s*-indacene (0.050 g, 0.060 mmol) and [ReCl(CO)$_3$(PPh$_3$)$_2$] (0.050 g, 0.060 mmol) were dissolved in anhydrous mesitylene (2 mL) and stirred at reflux for four hours. After passing the reaction mixture through a pad of silica, eluting first with hexane, followed by dichloromethane, an orange solid was obtained (0.051 g, 82%). A sample suitable for X-ray crystallographic analysis was obtained from tetrahydrofuran/pentane. **^{1}H NMR** (500 MHz, CDCl$_3$) δ 7.78-7.75 (m, 4H), 7.53-7.45 (m, 6H), 7.40-7.24 (m, 14H), 3.52-3.40 (m, 2H), 2.88-2.84 (m, 2H), 2.58-2.47 (m, 4H), 2.45 (s, 6H), 2.30 (q, $^3J_{HH}$ = 7.3 Hz, 4H), 1.14 (s, 6H), 0.99 (t, $^3J_{HH}$ = 7.3 Hz, 6H), 0.26 (s, 6H) ppm; **^{13}C{^{1}H} NMR** (126 MHz, CDCl$_3$) δ 201.8 (dt, $^2J_{CP}$ = 59.6 Hz, $^2J_{CP}$ = 6.3 Hz, CO), 196.2 (m, CO), 151.1, 140.0, 138.7, 137.8 (m), 134.4 (m), 133.3-131.7 (overlapping m), 129.8-128.2 (overlapping m), 33.1 (m), 25.5 (dt, $^1J_{CP}$ = 28.8 Hz, $^2J_{CP}$ = 6.6 Hz), 17.6, 14.8, 14.4, 11.8, 10.5 ppm; **^{31}P{^{1}H} NMR** (202 MHz, CDCl$_3$) δ 79.8 (1P), 37.7 (2P) ppm; **^{11}B{^{1}H} NMR** (160 MHz, CDCl$_3$) δ −2.0 ppm; **IR** (neat): $\tilde{\nu}$ = 1933 (s, CO), 1852 (s, CO) cm^{-1}; **HRMS** (ESI$^+$) calc. for $C_{55}H_{61}B_1Cl_1N_2O_2P_3Re_1$ [M+H]$^+$ requires m/z 1104.3288, found *m/z* 1104.3284 (0.4 ppm).

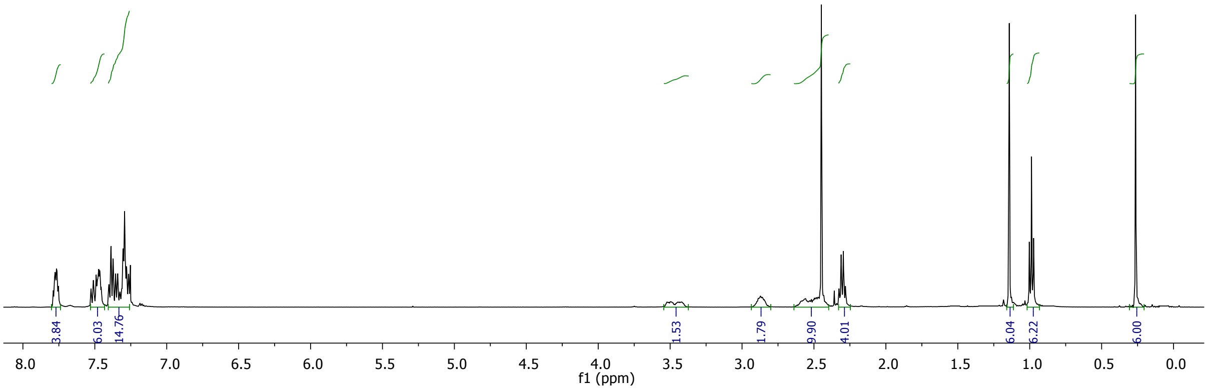

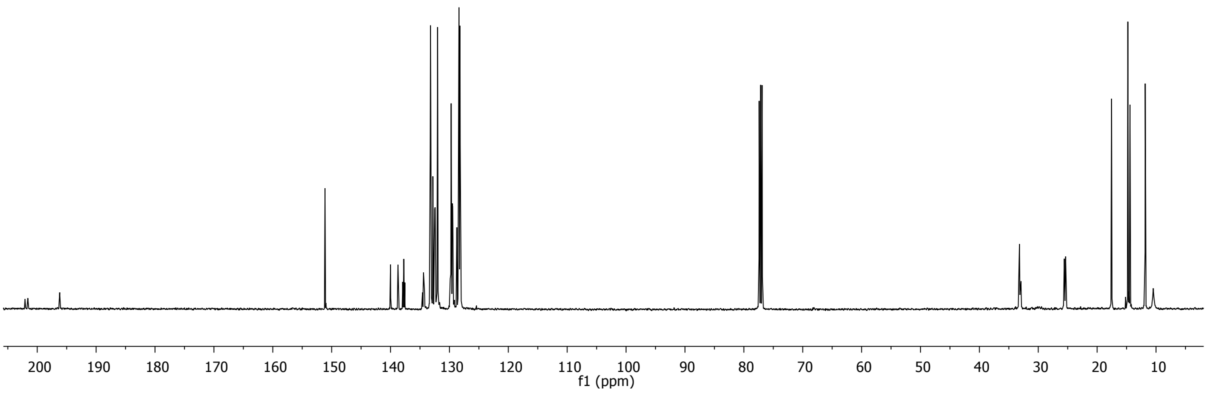

2.2 *cis,mer*(2)-[ReCl(CO)$_2$(2)] (3b)

8-(4-(Bis-2-ethyldiphenylphosphino)-phenylphosphino)-4,4-dimethyl-1,3,5,7-tetramethyl-2,6-diethyl-4-bora-3a,4a-diaza-*s*-indacene (0.050 g, 0.060 mmol) and [ReCl(CO)$_3$(PPh$_3$)$_2$] (0.050 g, 0.060 mmol) were dissolved in anhydrous toluene (5 mL) and stirred at reflux for seven hours. Purification was performed by column chromatography on silica gel (hexane/dichloromethane 1:1, R_f = 0.5) to produce an orange solid (0.044 g, 67%). A sample suitable for X-ray crystallographic analysis was obtained from tetrahydrofuran/pentane. **^{1}H NMR** (500 MHz, CDCl$_3$) δ 8.07-8.04 (m, 2H), 7.69.7.65 (m, 8H), 7.42.7.20 (m, 14H), 3.58-3.46 (m, 2H), 3.00-2.94 (m, 2H), 2.46-2.36 (br, 4H), 2.44 (s, 6H), 2.29 (q, $^3J_{HH}$ = 7.3 Hz, 4H), 1.18 (s, 6H), 0.98 (t, $^3J_{HH}$ = 7.3 Hz, 6H), 0.28 (s, 6H) ppm; **^{13}C{^{1}H} NMR** (126 MHz, CDCl$_3$) δ 202.5 (dt, $^2J_{CP}$ = 63.7 Hz), 190.0 (m, CO), 151.0, 140.2, 139.1 (m), 134.3-131.1 (overlapping m), 129.9-128.0 (overlapping m), 33.8 (m), 30.2 (m, J_{CP} = 31.9 Hz, J_{CP} = 8.0 Hz), 17.5, 14.8, 14.4, 11.9, 10.4 ppm; **^{31}P{^{1}H} NMR** (202 MHz, CDCl$_3$) δ 59.3 (1P), 29.6 (2P) ppm; **^{11}B{^{1}H} NMR** (160 MHz, CDCl$_3$) δ −2.0 ppm; **IR** (neat): $\tilde{\nu}$ = 1942 (s, CO), 1854 (s, CO) cm^{-1}; **HRMS** (ESI$^+$) calc. for $C_{55}H_{61}B_1Cl_1N_2O_2P_3Re_1$ [M+H]$^+$ requires m/z 1104.3288, found *m/z* 1104.3286 (0.2 ppm).

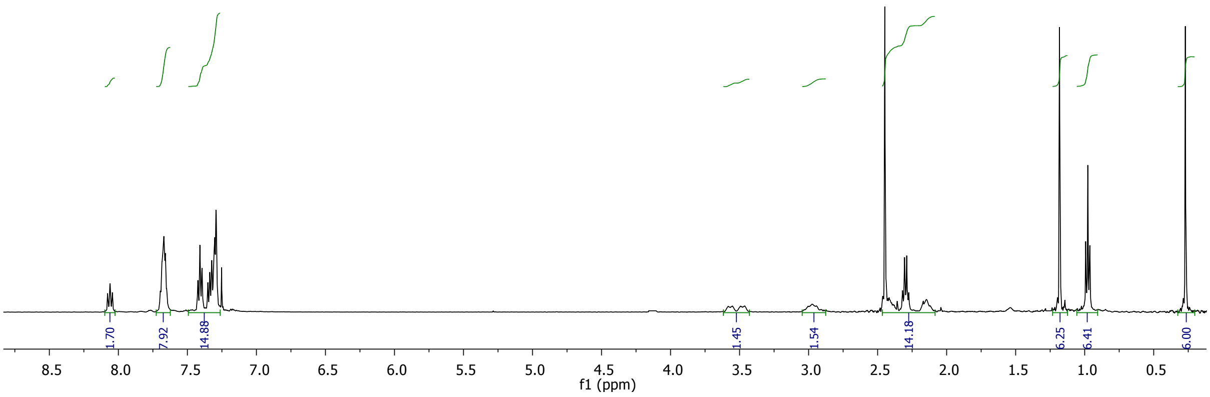

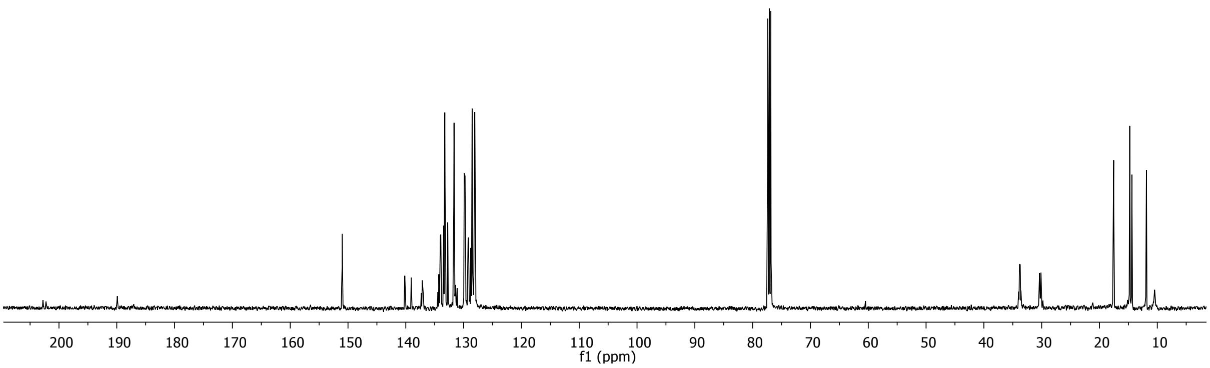

2.3 *cis,fac*-[ReCl(CO)$_2$(2)] (3c)

Prepared in the same manner as **3b**. Purification was performed by column chromatography on silica gel (dichloromethane, R_f = 0.4) to produce an orange solid (0.003 g, 4.5%). A sample suitable for X-ray crystallographic analysis was obtained from tetrahydrofuran/pentane. **^{1}H NMR** (400 MHz, $CDCl_3$) δ 7.95-7.91 (m, 4H), 7.59-7.55 (m, 2H), 7.38-7.29 (m, 12H), 6.99-6.97 (m, 2H), 6.87-6.83 (m, 4H), 2.86-2.52 (br, 8H), 2.42 (s, 6H), 2.38 (q, $^3J_{HH}$ = 7.3 Hz, 4H), 1.18 (s, 6H), 0.93 (t, $^3J_{HH}$ = 7.3 Hz, 6H), 0.25 (s, 6H) ppm; **^{31}P{^{1}H} NMR** (161 MHz, $CDCl_3$) δ 68.1 (t, $^2J_{PP}$ = 7.4 Hz, 1P), 13.3 (d, $^2J_{PP}$ = 7.4 Hz, 2P) ppm; **^{11}B{^{1}H} NMR** (128 MHz, $CDCl_3$) δ −2.0 ppm; **IR** (neat): $\tilde{\nu}$ = 1941 (s, CO), 1876 (s, CO) cm^{-1}; **HRMS** (ESI$^+$) calc. for $C_{55}H_{61}B_1Cl_1N_2O_2P_3Re_1$ [M+NH$_4$]$^+$ requires m/z 11121.3553, found *m/z* 1121.3555 (0.1 ppm).

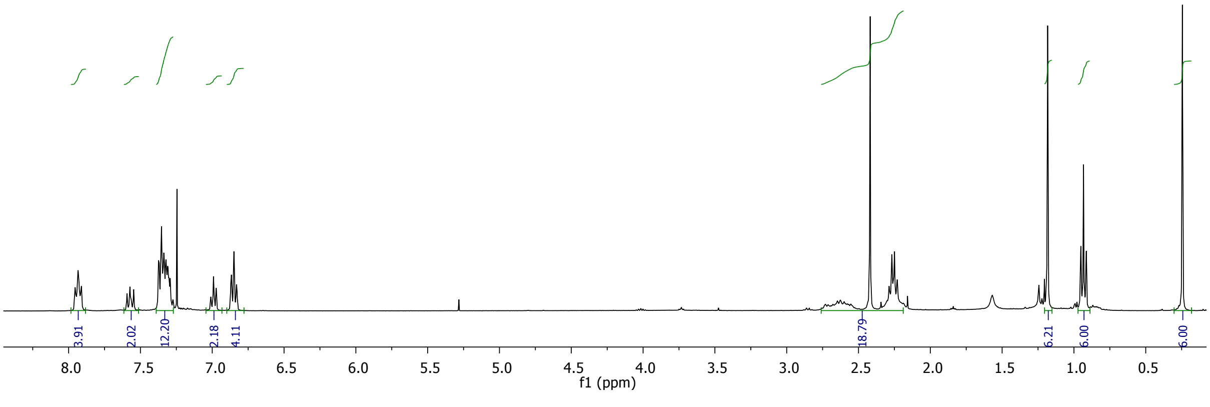

2.4 *fac*-[Re(CO)$_3$(triphos-Ph)][OTf] (4)

[Re(CO)$_5$][OTf] (0.015 g, 0.032 mmol) and triphos-Ph (0.017 g, 0.031 mmol) were added to a darkened flask and stirred at reflux in anhydrous ethanol (3 mL) for two hours, under nitrogen. The complex was purified using silica gel flash column chromatography with a

gradient from 1-5% ethanol in dichloromethane (dichloromethane/ethanol 100:3, $R_f = 0.1$) to yield a powdery white solid. This material was dissolved in ethanol/water and passed through a 3 mL PrepSep C18 cartridge, which had been equilibrated in water; the product was rinsed with 30% methanol in water to remove impurities and the product was eluted by flushing with methanol. Fractions containing pure product were combined and the solvent was removed to yield an off-white solid (0.010 g, 34%). A sample suitable for X-ray crystallographic analysis was obtained from tetrahydrofuran/pentane (slow diffusion).

^{1}H NMR (500 MHz, $CDCl_3$) δ 7.79 (m, 2H), 7.53 (s, 2H), 7.44-7.37 (m, 11H), 7.19-7.11 (m, 6H), 7.01-6.99 (m, 4H), 3.95 (m, 1H), 3.20 (m, 1H), 2.72 (br, 2H), 2.07 (br, 4H) ppm; **^{13}C{^{1}H} NMR** (126 MHz, $CDCl_3$) δ 194.1 (dt, $^{2}J_{CP}$ = 36.8 Hz), 188.6 (m, CO), 134.4 (m), 132.4, 132.0 (d, J_{CP} = 11.1 Hz), 131.1, 130.6, 129.8 (d, J_{CP} = 11.1 Hz), 129.3 (d, J_{CP} = 4.8 Hz), 127.0, 120.9 (q, $^{1}J_{CF}$ = 332.2 Hz, CF_3), 29.4 (m), 27.2 (m) ppm; **^{31}P{^{1}H} NMR** (202 MHz, $CDCl_3$) δ 66.4 (1P), 28.2 (2P) ppm; **IR** (neat): $\tilde{\nu}$ = 2957 (w), 2921 (w), 2851 (w), 2029 (s, CO), 1953 (s, CO), 1932 (s, CO), 1436 (m), 1260 (s), 1228 (w), 1147 (w), 1096 (m), 1030 (s), 999 (m), 806 (s) cm^{-1}; **HRMS** (ESI^{+}) calcd for $C_{37}H_{33}O_3P_3Re_1$ $[M]^{+}$ requires m/z 803.1167, found *m/z* 803.1167 (0.0 ppm).

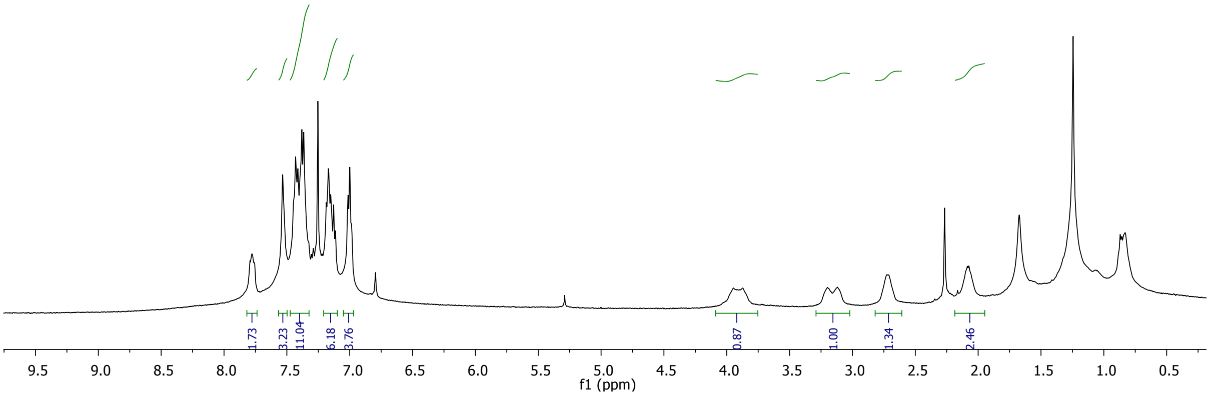

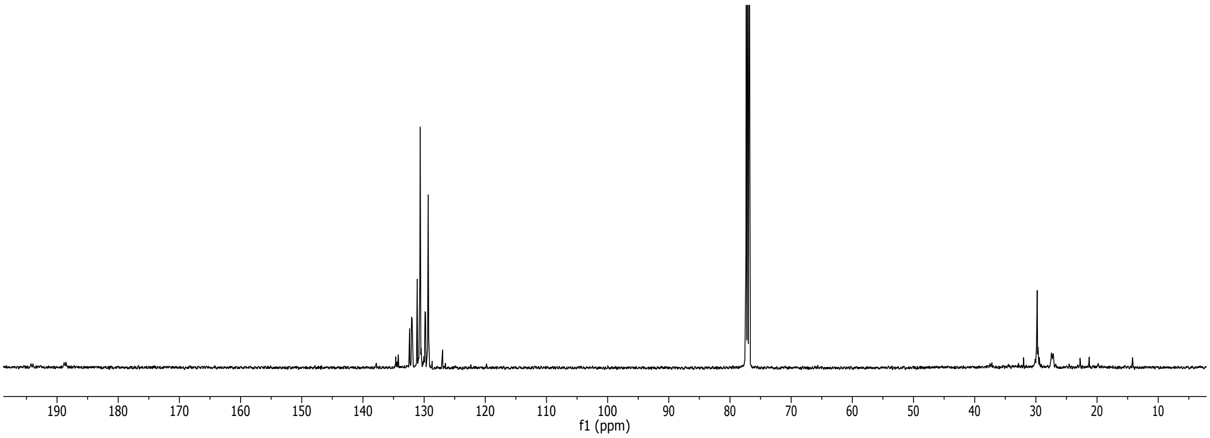

2.5 *fac*-[Re(CO)$_3$(2)][OTf] (5)

Method A:

[ReBr(CO)$_3$(3,4-diaminobenzoic acid)] (0.050 g, 0.100 mmol, 1.0 eq) and silver triflate (0.026 g, 0.100 mmol) were added to a darkened flask and stirred at room temperature in anhydrous ethanol (3 mL) for two hours, under nitrogen. A solid of silver bromide precipitated, which was filtered. To the filtrate, 8-(4-(bis-2-ethyldiphenylphosphino)-phenylphosphine)-4,4-dimethyl-1,3,5,7-tetramethyl-2,6-diethyl-4-bora-3a,4a-diaza-*s*-indacene (0.083 g, 0.100 mmol, 1.0 eq) was added and the reaction was stirred at 65 °C for 4 hours. The complex was purified using column chromatography on silica gel (chloroform/methanol 100:6, $R_f = 0.1$) to afford an orange solid (0.104 g, 84%). A sample suitable for X-ray crystallographic analysis was obtained from ethanol/pentane (slow diffusion).

Method B:

[Re(CO)$_5$][OTf] (0.018 g, 0.037 mmol) and 8-(4-(bis-2-ethyldiphenylphosphino)-phenylphosphine)-4,4-dimethyl-1,3,5,7-tetramethyl-2,6-diethyl-4-bora-3a,4a-diaza-*s*-indacene (0.030 g, 0.037 mmol) were added to a darkened flask and stirred at reflux in anhydrous ethanol (3 mL) for three hours, under nitrogen. The solvent was removed and the crude residue was purified by silica gel flash column chromatography with a gradient from 1-5% ethanol in dichloromethane (dichloromethane/ethanol 100:4, $R_f = 0.1$) to yield an amorphous deep red solid. This material was dissolved in ethanol/water and passed through a 3 mL PrepSep C18 cartridge, which had been equilibrated in water; the product was eluted by increasing the ratio of ethanol in water in 25% increments. Fractions containing pure product were combined and the solvent was removed to yield a deep red, glassy solid (0.013 g, 29%). **^{1}H NMR** (500 MHz, CDCl$_3$) δ 7.71 (m, 2H), 7.50-7.40 (m, 12H), 7.19-7.11 (m, 6H), 7.02-6.97 (m, 4H), 3.96 (m, 1H), 3.21 (m, 1H), 2.85 (m, 2H), 2.43 (s, 6H), 2.28 (br, 4H), 2.29 (q, $^3J_{HH}$ = 7.8 Hz, 4H), 1.19 (s, 6H), 0.96 (t, $^3J_{HH}$ = 7.8 Hz, 6H), 0.26 (s, 6H) ppm; **^{13}C{^{1}H} NMR** (126 MHz, CDCl$_3$) δ 194.1 (dt, $^2J_{CP}$ = 42.0 Hz, $^2J_{CP}$ = 8.6 Hz, CO), 188.7 (m, CO), 151.4, 141.5 (d, J_{CP} = 2.6 Hz), 137.9, 134.6, 134.2, 133.3 (d, J_{CP} = 40.9 Hz), 131.3, 131.0 (d, J_{CP} = 10.5 Hz), 130.9 (pseudo t, J_{CP} = 5.1 Hz), 130.7, 130.6 (pseudo t, J_{CP} = 5.1 Hz), 130.4 (d, J_{CP} = 10.7 Hz), 129.4 (overlapping m), 128.7, 121.1 (q, $^1J_{CF}$ = 320.2 Hz, CF$_3$), 29.7, 28.6 (m), 28.3 (m), 27.5 (m), 17.5, 14.7, 14.4, 12.0, 10.4 ppm; **^{31}P{^{1}H} NMR** (202 MHz, CDCl$_3$) δ 65.1 (1P), 29.5 (2P) ppm; **^{11}B NMR** (128 MHz, CDCl$_3$) δ −1.8 ppm; **IR** (neat): $\tilde{\nu}$ = 2964 (w), 2927 (w), 2854 (w), 2035 (s), 1946 (s), 1910 (s), 1558 (m), 1436 (w), 1361 (m), 1322 (m),

1259 (w), 1174 (s), 1147 (m), 1112 (m), 1030 (m), 983 (s), 946 (s) cm^{-1}; **HRMS** (ESI^{+}) calcd for $C_{56}H_{60}B_1N_2O_3P_3Re_1$ [M]$^{+}$ requires m/z 1096.3470, found *m/z* 1096.3460 (0.9 ppm).

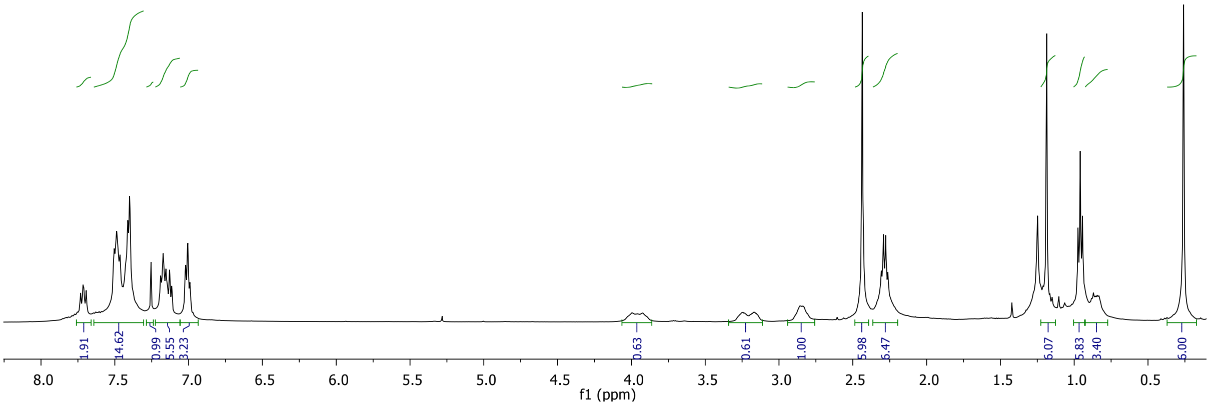

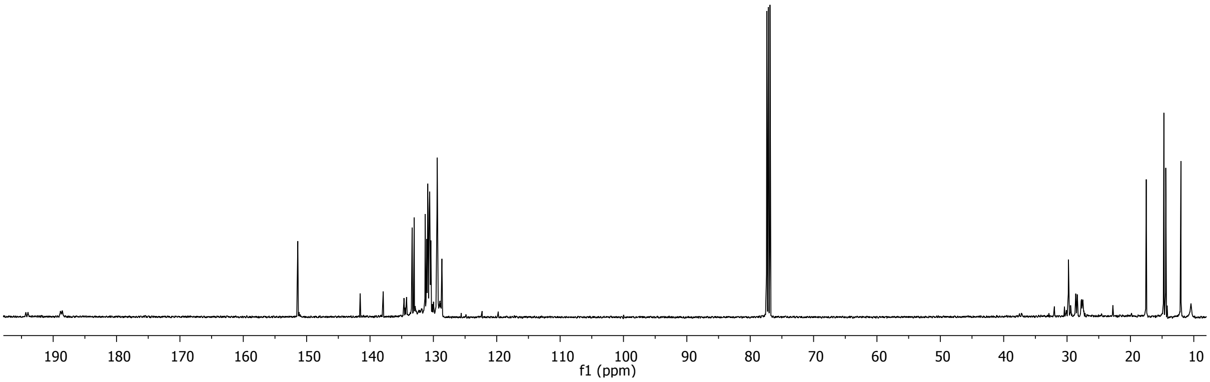

3 Radiochemistry

3.1 General Experimental Considerations

$[^{99m}TcO_4]^-$ (9.5-10 mCi) was obtained from Cardinal Health (Spokane, WA) and was used to prepare *fac*-$[^{99m}Tc(CO)_3(OH_2)_3]^+$ via commercially available Isolink® kits (Tyco, Inc.) as previously described.[7] A Perkin-Elmer Series 200 analytical chromatography system equipped with a Perkin-Elmer Radiomatic 610TR detector and a Hitachi D-7000 series analytical chromatography system (L-7100 pump, L-7400 UV detector) equipped with a Berthold FlowStar LB 513 radiodetector were used during reversed-phase HPLC (RP-HPLC) analysis of the rhenium and technetium complexes. Analytical separation and identification of the complexes were conducted using a Varian Pursuit XRs column (C18, 5 μm, 4.6 × 250 mm) and a Phenomenex security guard cartridge (C18, 4.0 × 3.0 mm) with the following gradient method and conditions: solvent A, 0.1% trifluoroacetic acid (TFA) in water; solvent B, methanol; 1 mL/min flow rate; 0-3 min 100% A, 3-9 min 75% A/25% B, 9-20 min linear gradient to 100% B, 9-25 min hold 100% B, step to 100% A at 30 min and equilibrate.

3.2 *fac*-$[^{99m}Tc(CO)_3(triphos\text{-}Ph)]^+$ (6)

Ethanol stock solutions of triphos-Ph (9.7×10^{-3} M to 9.7×10^{-5} M, 5 μL), ethanol (245 μL) and 10 mM pH 7.2 sodium phosphate buffer (200 μL) were added to 5 mL sealable labelling vials. The vials were sealed and purged with nitrogen for 5 min and a solution of *fac*-$[^{99m}Tc(CO)_3(OH_2)_3]^+$ (50 μL, 200-920 μCi) was added to the vial to bring the total volume to 0.5 mL, with final concentrations of triphos-Ph at 1 x 10^{-4} M to 1 x 10^{-6} M (constant 1:1 ratio of ethanol:aqueous solution). The vials were then heated at 85 °C with periodic analysis by RP-HPLC.

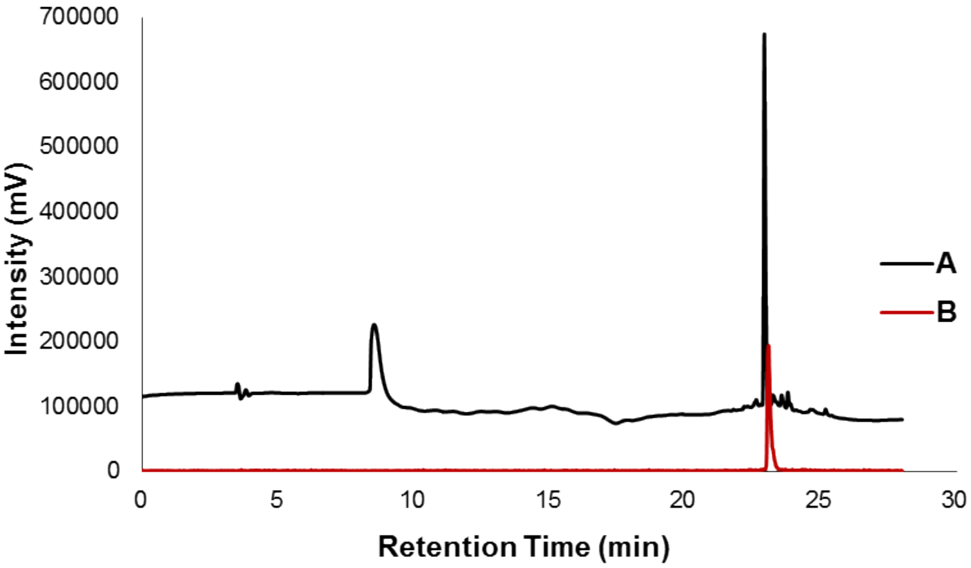

Fig. 3.1. Overlay of HPLC chromatograms from the crude reactions between triphos-Ph and $[Re(CO)_5]^+$ (black trace, UV, 220 nm) or *fac*-$[^{99m}Tc(CO)_3(OH_2)_3]^+$ (red trace, NaI γ-detector) to yield *fac*-$[Re(CO)_3(triphos\text{-}Ph)]^+$ (**4**) at 22.9 min (A) or *fac*-$[^{99m}Tc(CO)_3(triphos\text{-}Ph)]^+$ (**6**) at 23.0 min (B), respectively.

3.3 *fac*-$[^{99m}Tc(CO)_3(2)]^+$ (7)

***fac*-$[^{99m}Tc(CO)_3(2)]^+$ (7)**

Ethanol stock solutions of **2** (8.45×10^{-4} M or 8.45×10^{-5} M, 6 µL) and ethanol (444 µL) were added to 5 mL sealable labelling vials. The vials were sealed and purged with nitrogen for 5 min and a solution of *fac*-$[^{99m}Tc(CO)_3(OH_2)_3]^+$ (50 µL, 150-300 µCi) was added to the vial to bring the total volume to 0.5 mL, with final concentrations of **2** at 1 x 10^{-5} M or 1 x 10^{-6} M. The vials were then heated at 85 °C with periodic analysis by RP-HPLC.

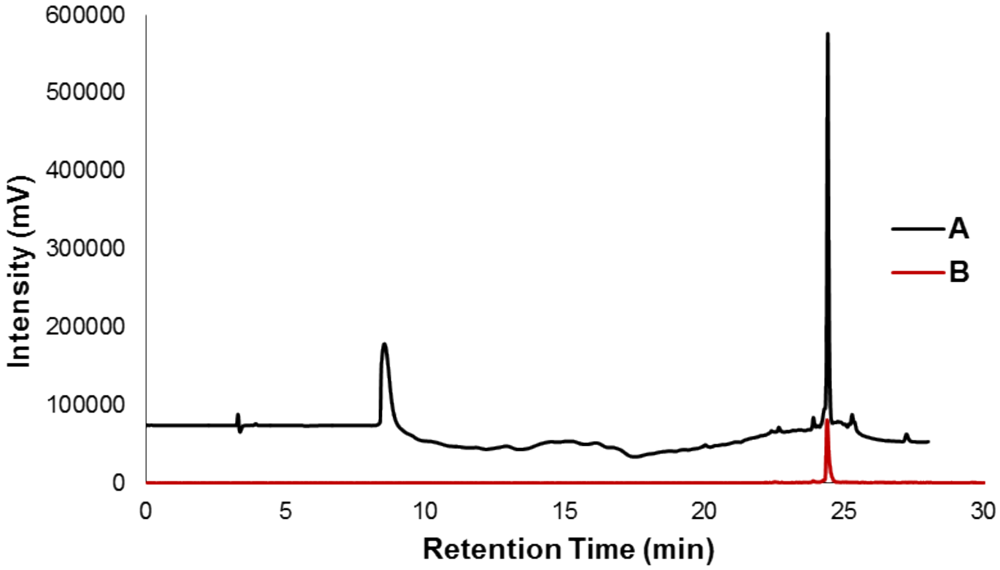

Fig. 3.2. Overlay of HPLC chromatograms from the crude reactions between **2** and $[Re(CO)_5]^+$ (black trace; UV, 220 nm) or *fac*-$[^{99m}Tc(CO)_3(OH_2)_3]^+$ (red trace, NaI γ-detector) to yield *fac*-$[Re(CO)_3(\mathbf{2})]^+$ (**5**) at 24.4 min (A) or *fac*-$[^{99m}Tc(CO)_3(\mathbf{2})]^+$ (**7**) at 24.5 min (B), respectively.

3.4 Stability Analysis

RP-HPLC purified solutions of **6** or **7** (200-250 µL) were added to an equal volume of 10 mM pH 7.2 sodium phosphate buffer or to solutions containing L-histidine or L-cysteine at 2 mM in 10 mM pH 7.2 sodium phosphate buffer in a sealable labelling vial (0.4-0.5 mL final solution volume). The vials were sealed, degassed with nitrogen, and incubated at 37 °C. Aliquots were periodically withdrawn and analysed by RP-HPLC to determine the stability of the parent peak to transchelation or degradation.

Table 3.1. Radiolabeling conversion[a] from the *fac*-$[^{99m}Tc(CO)_3(OH_2)_3]^+$ precursor based on starting concentrations of triphos-Ph or ligand **2** to form **6** or **7**, respectively. Reactions were heated at 85 °C and then allowed to cool briefly before aliquots were removed at the time points shown, for analysis by RP-HPLC.

Starting ligand	**6**		**7**	
concentration	30 min	60 min	30 min	60 min
**10^{-4} M**	>98%	>99%	--	--
**10^{-5} M**	>98%	>99%	>98%	>99%
**10^{-6} M**	40%	83%	86%	93%

[a]Radiochemical conversion (%) = ((1-(radiochemical area of the remaining *fac*-$[^{99m}Tc(CO)_3(OH_2)_3]^+$ precursor/radiochemical area of the entire chromatogram))×100).

Table 3.2. Stability analysis of RP-HPLC purified **6** and **7** following incubation at 37 °C in 10 mM pH 7.2 sodium phosphate buffer or in 10 mM pH 7.2 sodium phosphate buffer containing 1 mM histidine or 1 mM cysteine. Aliquots of the solutions were removed at the time points shown for analysis by RP-HPLC.

	**6**			**7**		
Solution	2 h	4 h	18.5 h	2 h	4 h	19.5 h
sodium phosphate buffer	>99%	>99%	>99%	>99%	99%	>99%
1 mM histidine	>99%	89%	>99%	>99%	97%	>99%
1 mM cysteine	>99%	97%	>99%	>99%	>99%	>99%

4 Cell Imaging

4.1 Cell Culturing and Cell Preparation

Cells were cultured at 37 °C in a humidified atmosphere in air and diluted once confluence had been reached. Culture occurred in RPMI (Roswell Park Memorial Institute) 1640 medium for PC3 (prostate cancer cells). The media contained 10% foetal calf serum (FCS), 0.5% penicillin/streptomycin (10,000 IU mL^{-1}/10,000 mg mL^{-1}) and 200 mM L-Glutamine (5 mL). All steps were performed in absence of phenol red. Surplus supernatant containing dead cell matter and excess protein was aspirated. The live adherent cells were then washed with 2 x 10 mL aliquots of phosphate buffer saline (PBS) solution to remove any remaining media containing FCS, which inactivates trypsin. Cells were resuspended in solution by incubation in 3 mL of trypsin–PBS solution (0.25% trypsin) for 5 min at 37 °C. After trypsinisation, 5 mL of medium containing 10% serum was added to inactivate the trypsin and the solution was centrifuged for 5 min (1000 rpm, 25 ºC) to remove any remaining dead cell matter. The supernatant liquid was aspirated and 5 mL of cell medium (10% FCS) was added to the cell matter left behind. Cells were counted using a haemocytometer and then seeded as appropriate.

4.2 MTT Assays

Cells cultured as above were plated (5 x 10^4 cells mL^{-1}) and left for 48 h to adhere fully. All steps were carried out in the absence of phenol red. (a) For MI_{50} estimations by MTT assays, cells were incubated with each compound tested for 48 h at 37 ˚C. Concentrations used were 1 nM, 50 nM, 100 nM, 1 μM, 10 μM, 50 μM, 100 μM and 250 μM (1% DMSO, 99% Eagle's Modified Essential Medium containing FCS at standard concentration of the cell line). Subsequently, cells were washed three times with PBS and 100 mL of MTT were added (0.5 mg mL^{-1}, 10% PBS:SFM) followed by a 2 h incubation. Following aspiration, 100 mL of DMSO was added and 96 well plates were read at an ELISA plate reader, Fluostar Omega BMGLabTech. Data were obtained from at least three consistent results and MI_{50} was calculated using Origin 8 as half the height of the fitted curve for each compound and for each individual experiment. Due to background absorbance, 100% cell death would not correspond to zero absorbance, therefore the height of the curve was calculated as the highest absorbance of the fit plot minus the minimum absorbance of the curve, at which point death of all cells has been achieved. This value therefore indicates the minimum MI_{50} for this

compound and is stated in the text/figure legend. The mean MI_{50} was calculated from the average of the five MI_{50} values obtained from five consistent experiments. The error reported was the standard error of the mean and shown as ± S.E.M.

4.3 Fluorescence Microscopy

For confocal fluorescence microscopy experiments, cells were cultured as above and plated in glass-bottomed dishes as 1.5 x 10^5 cells per dish (*ca.* 60% coverage) and incubated for 72 h. All steps were carried out in the absence of phenol red. Prior to compound addition, cells were washed three times with PBS, before adding serum free medium (SFM) or PBS (990 μL). 10 μL of compound in DMSO was added to give a 1% solution. The final concentration of compounds on the cell plate for example was 100 μM in medium for a 10 mM stock solution (whereby 990 μL SFM was added originally for obtaining a 1% solution). After 15 min incubation with the compound, the cells were washed three times with PBS, fresh SFM was added (1 mL) and followed by recording of images. Cell imaging was carried out on a Zeiss LSM510META, a modified Nikon TE2000-U or a Nikon Eclipse TE2000 epi-fluorescence instrument.

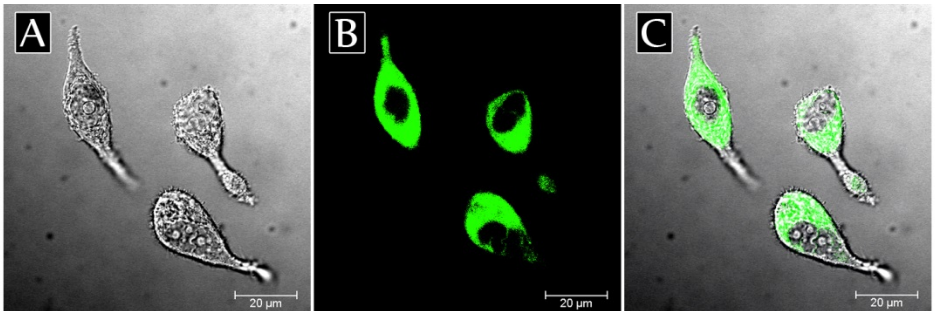

Fig. 4.1. Single-photon confocal microscopy images of a 10 μM solution of tridentate phosphine, **2** in PC-3: (A) DIC image; (B) excitation at 488 nm, long pass filtered at 510 nm, (C) an overlay of the previous three images.

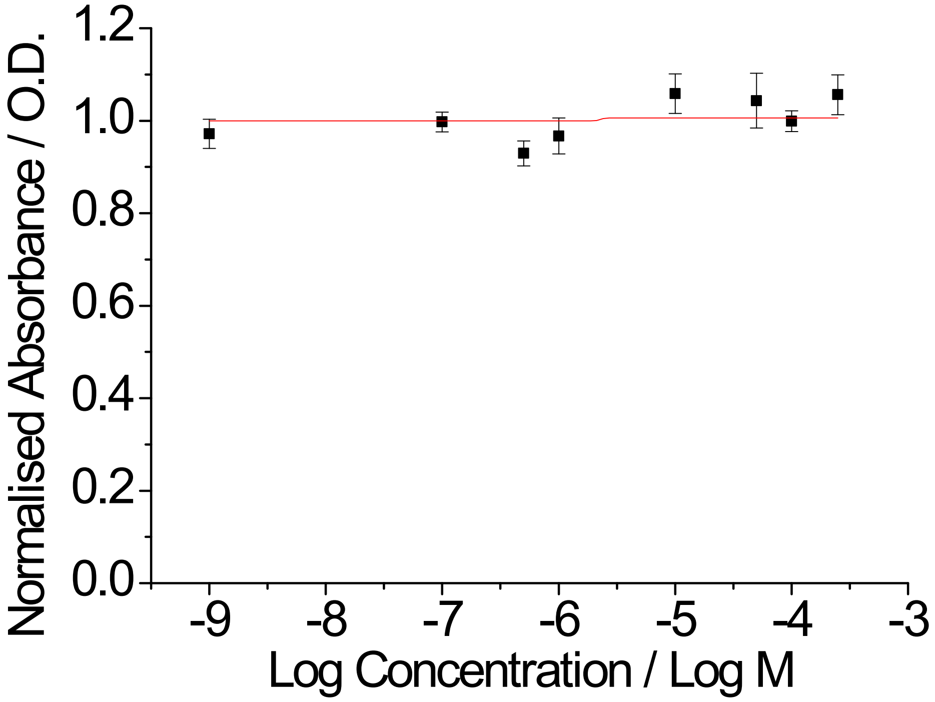

Fig. 4.2. MTT assays of *cis,mer*-[ReCl(CO)$_2$(**2**)], **3a**, in PC-3 cells, whereby the MI_{50} was determined to be > 250 μM.

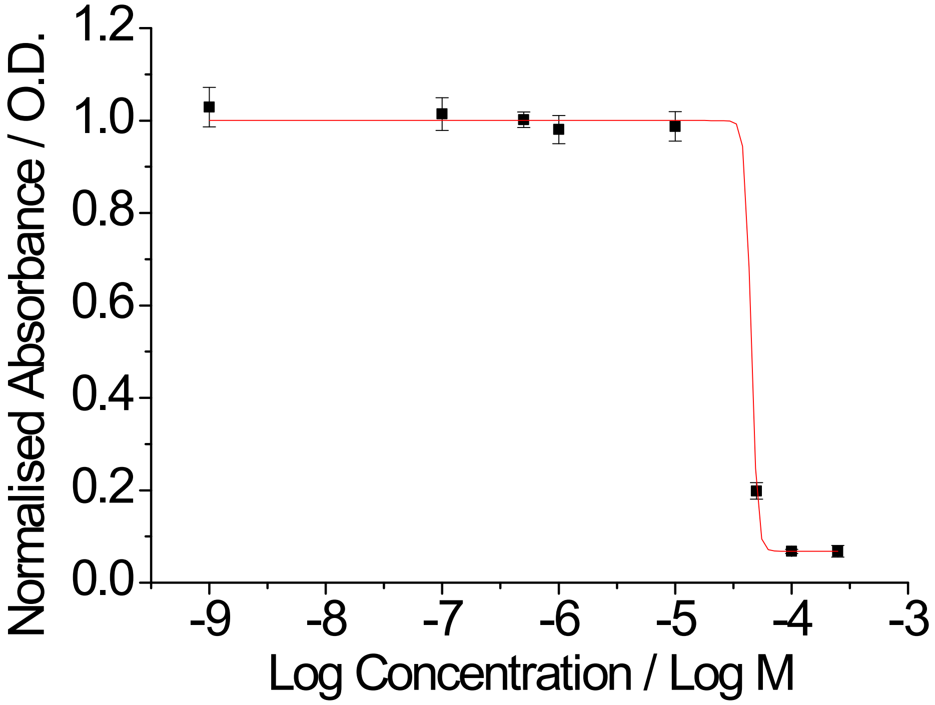

Fig. 4.3. MTT assays of *fac*-[Re(CO)$_3$(**2**)][OTf], **5**, in PC-3 cells, whereby the MI_{50} was determined to be 45 μM ± 5 μM.

5 X-ray Crystallography

5.1 General Information

Data were collected at 150(2) K on an Agilent Technologies Gemini A Ultra diffractometer with Mo*K*α (λ = 0.71073 Å for **3a**, and **4**) or Cu*K*α (λ = 1.54178 Å, for **3b**, **3c**, and **5**) radiation.[8] Selected crystallographic information is given in the following tables. Absorption corrections were based on multiple and symmetry-equivalent data; the structures were solved by direct methods, and refined on all unique F^2 values with appropriate constraints and/or restraints in each case, particularly for the treatment of disordered structural components.[9] Unidentified solvent in the structures of **3a** and **3c** was treated by the Squeeze procedure of PLATON.[10] CCDC references: 849753 (**3a**), 101450 (**3b**), 101449 (**3c**), 101451 (**4**) and 101452 (**5**).

5.2 Crystallographic data for *cis*,*mer*(2)-[ReCl(CO)$_2$(2)](3b)

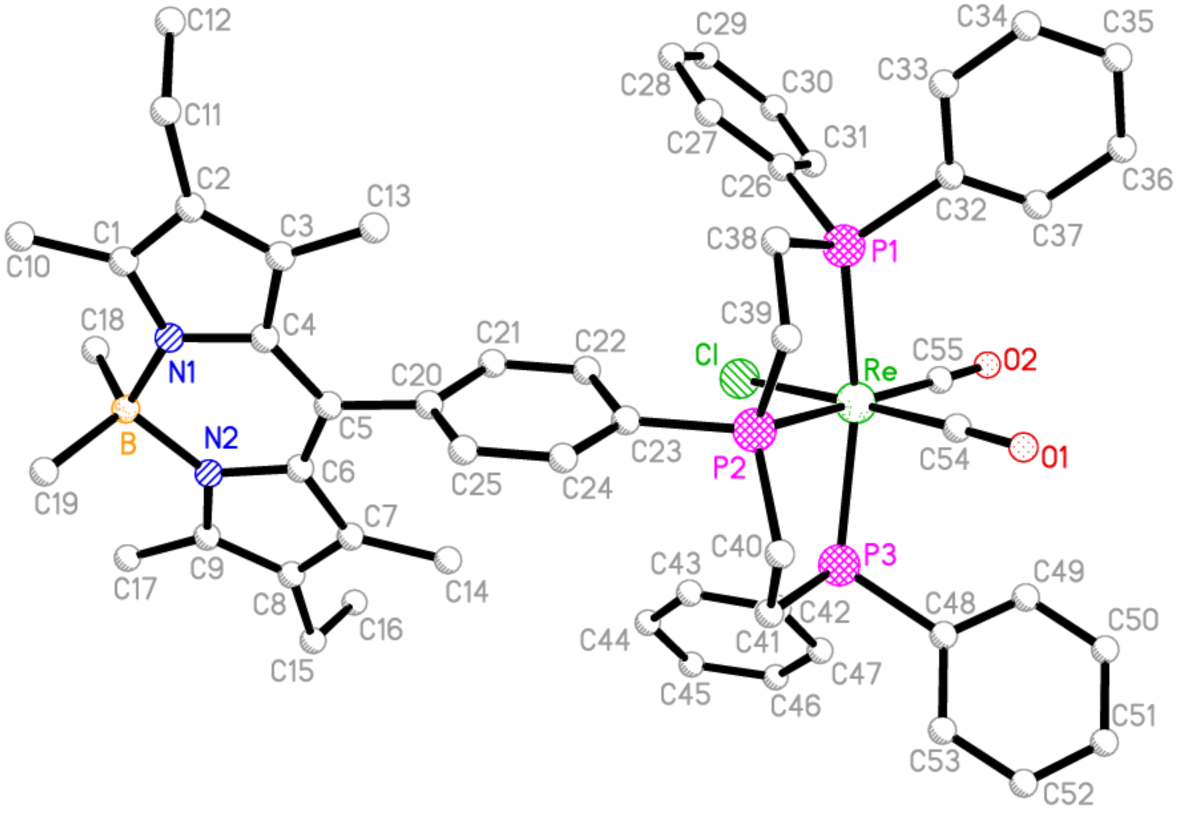

Table 3b:1. Crystal data and structure refinement for ljh117 (**3b**).

Identification code	ljh117 (**3b**)	
Chemical formula (moiety)	$C_{55}H_{60}BClN_2O_2P_3Re$	
Chemical formula (total)	$C_{55}H_{60}BClN_2O_2P_3Re$	
Formula weight	1106.42	
Temperature	150(2) K	
Radiation, wavelength	CuKα, 1.54178 Å	
Crystal system, space group	monoclinic, $P2_1/c$	
Unit cell parameters	a = 12.7603(4) Å	α = 90°
	b = 26.1035(8) Å	β = 106.521(3)°
	c = 15.6208(4) Å	γ = 90°
Cell volume	4988.3(3) $Å^3$	
Z	4	
Calculated density	1.473 g/cm^3	
Absorption coefficient μ	6.500 mm^{-1}	
F(000)	2248	
Crystal colour and size	red, 0.15 × 0.10 × 0.02 mm^3	
Reflections for cell refinement	6037 (θ range 3.0 to 62.3°)	
Data collection method	Agilent Technologies Gemini A Ultra	
	ω scans	
θ range for data collection	3.4 to 62.4°	
Index ranges	h −11 to 14, k −29 to 27, l −17 to 16	
Completeness to θ = 67.7°	85.5 %	
Reflections collected	16817	
Independent reflections	7730 (R_{int} = 0.0413)	
Reflections with F^2>2σ	6049	
Absorption correction	multi-scan	
Min. and max. transmission	0.440 and 0.881	
Structure solution	direct methods	
Refinement method	Full-matrix least-squares on F^2	
Weighting parameters a, b	0.0457,	
Data / restraints / parameters	7730 / 0 / 594	

Final R indices [$F^2 > 2\sigma$]	R1 = 0.0408, wR2 = 0.0898
R indices (all data)	R1 = 0.0589, wR2 = 0.1001
Goodness-of-fit on F^2	1.031
Largest and mean shift/su	0.002 and 0.000
Largest diff. peak and hole	2.24 and −1.31 e Å^{-3}

Table 3b:2. Atomic coordinates and equivalent isotropic displacement parameters (Å^2) for ljh117 (**3b**). U_{eq} is defined as one third of the trace of the orthogonalised U^{ij} tensor.

	x	y	z	U_{eq}
Re	0.21067(2)	0.17275(2)	0.47918(2)	0.02352(9)
B	0.2871(6)	0.5785(3)	0.5233(4)	0.0367(16)
N1	0.3779(4)	0.5444(2)	0.5920(3)	0.0364(12)
N2	0.1933(4)	0.53935(19)	0.4761(3)	0.0345(12)
C1	0.4758(5)	0.5607(3)	0.6434(4)	0.0423(16)
C2	0.5353(5)	0.5187(3)	0.6911(4)	0.0430(17)
C3	0.4706(5)	0.4754(3)	0.6695(4)	0.0400(15)
C4	0.3712(5)	0.4919(2)	0.6059(3)	0.0331(14)
C5	0.2794(5)	0.4636(2)	0.5582(3)	0.0333(14)
C6	0.1949(4)	0.4865(2)	0.4938(3)	0.0263(12)
C7	0.0953(5)	0.4646(2)	0.4380(4)	0.0330(14)
C8	0.0399(5)	0.5039(3)	0.3853(4)	0.0375(15)
C9	0.1013(5)	0.5494(2)	0.4096(4)	0.0372(15)
C10	0.5141(5)	0.6138(3)	0.6513(5)	0.0551(19)
C11	0.6475(5)	0.5218(3)	0.7540(4)	0.0522(19)
C12	0.7381(5)	0.5205(4)	0.7083(4)	0.067(2)
C13	0.4987(5)	0.4233(3)	0.7085(4)	0.0484(18)
C14	0.0559(5)	0.4105(3)	0.4310(4)	0.0433(16)
C15	−0.0655(5)	0.4998(3)	0.3116(4)	0.0439(16)
C16	−0.0520(5)	0.4787(3)	0.2252(4)	0.0527(19)
C17	0.0718(6)	0.6008(3)	0.3686(5)	0.0537(18)
C18	0.3424(5)	0.6016(3)	0.4499(4)	0.0491(17)
C19	0.2389(5)	0.6188(3)	0.5803(4)	0.0458(17)
C20	0.2743(4)	0.4081(2)	0.5731(3)	0.0318(13)
C21	0.3009(5)	0.3734(2)	0.5151(4)	0.0357(14)
C22	0.2851(5)	0.3217(2)	0.5218(3)	0.0337(14)
C23	0.2420(4)	0.3025(2)	0.5884(3)	0.0282(13)
C24	0.2236(5)	0.3370(2)	0.6509(3)	0.0337(14)

C25	0.2394(5)	0.3890(2)	0.6434(4)	0.0334(14)
P1	0.40512(11)	0.17473(6)	0.54507(8)	0.0249(3)
P2	0.22112(11)	0.23424(6)	0.59690(8)	0.0246(3)
P3	0.01767(11)	0.18928(6)	0.45130(9)	0.0276(3)
C26	0.4910(4)	0.2016(2)	0.4796(3)	0.0285(13)
C27	0.5657(5)	0.2409(3)	0.5081(4)	0.0411(16)
C28	0.6295(5)	0.2577(3)	0.4554(4)	0.0416(16)
C29	0.6192(5)	0.2362(3)	0.3731(4)	0.0407(16)
C30	0.5446(5)	0.1978(3)	0.3437(4)	0.0418(16)
C31	0.4794(5)	0.1803(2)	0.3957(4)	0.0398(15)
C32	0.4813(4)	0.1159(2)	0.5856(3)	0.0268(13)
C33	0.5893(5)	0.1188(3)	0.6393(4)	0.0371(15)
C34	0.6483(5)	0.0752(3)	0.6702(4)	0.0446(17)
C35	0.6023(5)	0.0270(3)	0.6475(4)	0.0463(17)
C36	0.4958(5)	0.0235(3)	0.5949(4)	0.0454(16)
C37	0.4361(5)	0.0686(2)	0.5639(4)	0.0360(14)
C38	0.4356(4)	0.2153(2)	0.6473(3)	0.0318(14)
C39	0.3396(4)	0.2160(2)	0.6882(3)	0.0282(13)
C40	0.0935(4)	0.2278(2)	0.6269(3)	0.0282(13)
C41	0.0006(4)	0.2335(2)	0.5401(3)	0.0323(14)
C42	−0.0608(4)	0.2227(2)	0.3510(3)	0.0302(13)
C43	−0.0383(5)	0.2736(2)	0.3373(4)	0.0359(14)
C44	−0.0963(5)	0.2995(3)	0.2608(4)	0.0453(16)
C45	−0.1764(5)	0.2746(3)	0.1966(4)	0.0437(17)
C46	−0.1973(5)	0.2237(3)	0.2070(4)	0.0454(17)
C47	−0.1405(5)	0.1979(3)	0.2838(3)	0.0379(15)
C48	−0.0706(4)	0.1347(2)	0.4571(3)	0.0321(14)
C49	−0.0413(5)	0.0854(2)	0.4406(4)	0.0371(14)
C50	−0.1079(5)	0.0441(3)	0.4428(4)	0.0462(17)
C51	−0.2051(5)	0.0518(3)	0.4628(4)	0.0488(17)
C52	−0.2368(5)	0.1000(3)	0.4784(4)	0.0493(18)
C53	−0.1714(5)	0.1417(3)	0.4744(4)	0.0406(16)
C54	0.1950(4)	0.1239(2)	0.5629(4)	0.0281(13)

O1	0.1817(3)	0.09363(17)	0.6132(3)	0.0427(11)
C55	0.2070(4)	0.1186(2)	0.3933(4)	0.0308(13)
O2	0.2077(3)	0.08616(18)	0.3442(3)	0.0447(11)
Cl	0.22012(11)	0.23309(6)	0.35675(8)	0.0329(3)

Table 3b:3. Bond lengths [Å] and angles [°] for ljh117 (**3b**).

Re–P1	2.4012(14)	Re–P2	2.4157(13)
Re–P3	2.4147(14)	Re–C54	1.878(6)
Re–C55	1.940(6)	Re–Cl	2.5068(13)
B–N1	1.605(9)	B–N2	1.586(8)
B–C18	1.624(9)	B–C19	1.609(9)
N1–C1	1.347(7)	N1–C4	1.395(8)
N2–C6	1.407(7)	N2–C9	1.354(7)
C1–C2	1.417(9)	C1–C10	1.463(9)
C2–C3	1.385(9)	C2–C11	1.491(8)
C3–C4	1.437(8)	C3–C13	1.491(9)
C4–C5	1.405(8)	C5–C6	1.384(7)
C5–C20	1.472(8)	C6–C7	1.438(7)
C7–C8	1.378(8)	C7–C14	1.493(8)
C8–C9	1.412(9)	C8–C15	1.506(8)
C9–C17	1.488(9)	C10–H10A	0.980
C10–H10B	0.980	C10–H10C	0.980
C11–H11A	0.990	C11–H11B	0.990
C11–C12	1.523(8)	C12–H12A	0.980
C12–H12B	0.980	C12–H12C	0.980
C13–H13A	0.980	C13–H13B	0.980
C13–H13C	0.980	C14–H14A	0.980
C14–H14B	0.980	C14–H14C	0.980
C15–H15A	0.990	C15–H15B	0.990
C15–C16	1.511(8)	C16–H16A	0.980
C16–H16B	0.980	C16–H16C	0.980
C17–H17A	0.980	C17–H17B	0.980
C17–H17C	0.980	C18–H18A	0.980
C18–H18B	0.980	C18–H18C	0.980
C19–H19A	0.980	C19–H19B	0.980
C19–H19C	0.980	C20–C21	1.389(8)
C20–C25	1.389(7)	C21–H21	0.950
C21–C22	1.375(8)	C22–H22	0.950

C22–C23	1.401(7)	C23–C24	1.396(7)
C23–P2	1.811(6)	C24–H24	0.950
C24–C25	1.383(8)	C25–H25	0.950
P1–C26	1.837(5)	P1–C32	1.831(6)
P1–C38	1.863(5)	P2–C39	1.822(5)
P2–C40	1.826(5)	P3–C41	1.864(5)
P3–C42	1.824(6)	P3–C48	1.833(6)
C26–C27	1.385(8)	C26–C31	1.393(8)
C27–H27	0.950	C27–C28	1.383(8)
C28–H28	0.950	C28–C29	1.374(8)
C29–H29	0.950	C29–C30	1.370(9)
C30–H30	0.950	C30–C31	1.394(8)
C31–H31	0.950	C32–C33	1.396(7)
C32–C37	1.366(8)	C33–H33	0.950
C33–C34	1.373(8)	C34–H34	0.950
C34–C35	1.390(9)	C35–H35	0.950
C35–C36	1.376(9)	C36–H36	0.950
C36–C37	1.411(8)	C37–H37	0.950
C38–H38A	0.990	C38–H38B	0.990
C38–C39	1.534(7)	C39–H39A	0.990
C39–H39B	0.990	C40–H40A	0.990
C40–H40B	0.990	C40–C41	1.534(7)
C41–H41A	0.990	C41–H41B	0.990
C42–C43	1.387(8)	C42–C47	1.396(8)
C43–H43	0.950	C43–C44	1.389(8)
C44–H44	0.950	C44–C45	1.374(9)
C45–H45	0.950	C45–C46	1.375(9)
C46–H46	0.950	C46–C47	1.386(8)
C47–H47	0.950	C48–C49	1.385(8)
C48–C53	1.400(8)	C49–H49	0.950
C49–C50	1.380(8)	C50–H50	0.950
C50–C51	1.377(9)	C51–H51	0.950
C51–C52	1.365(10)	C52–H52	0.950
C52–C53	1.384(9)	C53–H53	0.950

C54–O1	1.161(6)	C55–O2	1.144(7)

P1–Re–P2	80.37(5)	P1–Re–P3	161.81(5)
P1–Re–C54	91.02(17)	P1–Re–C55	97.51(16)
P1–Re–Cl	92.61(5)	P2–Re–P3	81.45(5)
P2–Re–C54	85.10(17)	P2–Re–C55	174.63(17)
P2–Re–Cl	99.16(5)	P3–Re–C54	86.66(17)
P3–Re–C55	100.53(16)	P3–Re–Cl	91.03(5)
C54–Re–C55	90.0(2)	C54–Re–Cl	174.82(16)
C55–Re–Cl	85.84(17)	N1–B–N2	105.1(5)
N1–B–C18	107.6(5)	N1–B–C19	108.0(5)
N2–B–C18	109.5(5)	N2–B–C19	108.8(5)
C18–B–C19	117.3(6)	B–N1–C1	126.5(6)
B–N1–C4	125.7(5)	C1–N1–C4	107.7(5)
B–N2–C6	125.0(5)	B–N2–C9	127.4(5)
C6–N2–C9	107.5(5)	N1–C1–C2	109.8(6)
N1–C1–C10	125.4(6)	C2–C1–C10	124.8(6)
C1–C2–C3	108.1(6)	C1–C2–C11	125.2(7)
C3–C2–C11	126.8(7)	C2–C3–C4	105.8(6)
C2–C3–C13	126.0(6)	C4–C3–C13	128.2(6)
N1–C4–C3	108.7(5)	N1–C4–C5	120.9(5)
C3–C4–C5	130.3(6)	C4–C5–C6	121.2(6)
C4–C5–C20	120.1(5)	C6–C5–C20	118.6(5)
N2–C6–C5	122.0(5)	N2–C6–C7	108.0(5)
C5–C6–C7	129.9(5)	C6–C7–C8	106.4(5)
C6–C7–C14	129.8(5)	C8–C7–C14	123.8(5)
C7–C8–C9	108.3(5)	C7–C8–C15	126.7(6)
C9–C8–C15	124.9(6)	N2–C9–C8	109.7(6)
N2–C9–C17	124.2(6)	C8–C9–C17	126.1(6)
C1–C10–H10A	109.5	C1–C10–H10B	109.5
C1–C10–H10C	109.5	H10A–C10–H10B	109.5
H10A–C10–H10C	109.5	H10B–C10–H10C	109.5
C2–C11–H11A	108.8	C2–C11–H11B	108.8
C2–C11–C12	113.8(5)	H11A–C11–H11B	107.7

H11A–C11–C12	108.8	H11B–C11–C12	108.8
C11–C12–H12A	109.5	C11–C12–H12B	109.5
C11–C12–H12C	109.5	H12A–C12–H12B	109.5
H12A–C12–H12C	109.5	H12B–C12–H12C	109.5
C3–C13–H13A	109.5	C3–C13–H13B	109.5
C3–C13–H13C	109.5	H13A–C13–H13B	109.5
H13A–C13–H13C	109.5	H13B–C13–H13C	109.5
C7–C14–H14A	109.5	C7–C14–H14B	109.5
C7–C14–H14C	109.5	H14A–C14–H14B	109.5
H14A–C14–H14C	109.5	H14B–C14–H14C	109.5
C8–C15–H15A	108.8	C8–C15–H15B	108.8
C8–C15–C16	114.0(5)	H15A–C15–H15B	107.7
H15A–C15–C16	108.8	H15B–C15–C16	108.8
C15–C16–H16A	109.5	C15–C16–H16B	109.5
C15–C16–H16C	109.5	H16A–C16–H16B	109.5
H16A–C16–H16C	109.5	H16B–C16–H16C	109.5
C9–C17–H17A	109.5	C9–C17–H17B	109.5
C9–C17–H17C	109.5	H17A–C17–H17B	109.5
H17A–C17–H17C	109.5	H17B–C17–H17C	109.5
B–C18–H18A	109.5	B–C18–H18B	109.5
B–C18–H18C	109.5	H18A–C18–H18B	109.5
H18A–C18–H18C	109.5	H18B–C18–H18C	109.5
B–C19–H19A	109.5	B–C19–H19B	109.5
B–C19–H19C	109.5	H19A–C19–H19B	109.5
H19A–C19–H19C	109.5	H19B–C19–H19C	109.5
C5–C20–C21	120.6(5)	C5–C20–C25	121.0(5)
C21–C20–C25	118.4(6)	C20–C21–H21	119.3
C20–C21–C22	121.5(5)	H21–C21–C22	119.3
C21–C22–H22	119.8	C21–C22–C23	120.4(5)
H22–C22–C23	119.8	C22–C23–C24	117.9(6)
C22–C23–P2	120.2(4)	C24–C23–P2	121.8(4)
C23–C24–H24	119.4	C23–C24–C25	121.2(5)
H24–C24–C25	119.4	C20–C25–C24	120.4(5)
C20–C25–H25	119.8	C24–C25–H25	119.8

Re–P1–C26	118.48(17)	Re–P1–C32	120.84(19)
Re–P1–C38	108.54(17)	C26–P1–C32	99.8(2)
C26–P1–C38	104.3(3)	C32–P1–C38	102.7(3)
Re–P2–C23	125.10(17)	Re–P2–C39	106.04(18)
Re–P2–C40	105.91(18)	C23–P2–C39	102.0(3)
C23–P2–C40	105.6(3)	C39–P2–C40	112.2(2)
Re–P3–C41	107.78(17)	Re–P3–C42	121.55(18)
Re–P3–C48	117.49(19)	C41–P3–C42	101.0(3)
C41–P3–C48	103.9(3)	C42–P3–C48	102.7(3)
P1–C26–C27	124.7(4)	P1–C26–C31	116.8(4)
C27–C26–C31	118.5(5)	C26–C27–H27	119.6
C26–C27–C28	120.8(6)	H27–C27–C28	119.6
C27–C28–H28	119.6	C27–C28–C29	120.8(6)
H28–C28–C29	119.6	C28–C29–H29	120.5
C28–C29–C30	119.1(5)	H29–C29–C30	120.5
C29–C30–H30	119.5	C29–C30–C31	121.1(6)
H30–C30–C31	119.5	C26–C31–C30	119.8(6)
C26–C31–H31	120.1	C30–C31–H31	120.1
P1–C32–C33	119.9(5)	P1–C32–C37	121.8(4)
C33–C32–C37	118.2(5)	C32–C33–H33	119.5
C32–C33–C34	121.0(6)	H33–C33–C34	119.5
C33–C34–H34	119.7	C33–C34–C35	120.6(6)
H34–C34–C35	119.7	C34–C35–H35	120.4
C34–C35–C36	119.1(6)	H35–C35–C36	120.4
C35–C36–H36	120.2	C35–C36–C37	119.6(6)
H36–C36–C37	120.2	C32–C37–C36	121.4(6)
C32–C37–H37	119.3	C36–C37–H37	119.3
P1–C38–H38A	109.3	P1–C38–H38B	109.3
P1–C38–C39	111.5(4)	H38A–C38–H38B	108.0
H38A–C38–C39	109.3	H38B–C38–C39	109.3
P2–C39–C38	105.1(3)	P2–C39–H39A	110.7
P2–C39–H39B	110.7	C38–C39–H39A	110.7
C38–C39–H39B	110.7	H39A–C39–H39B	108.8
P2–C40–H40A	110.4	P2–C40–H40B	110.4

P2–C40–C41	106.6(3)	H40A–C40–H40B	108.6
H40A–C40–C41	110.4	H40B–C40–C41	110.4
P3–C41–C40	111.9(4)	P3–C41–H41A	109.2
P3–C41–H41B	109.2	C40–C41–H41A	109.2
C40–C41–H41B	109.2	H41A–C41–H41B	107.9
P3–C42–C43	120.2(4)	P3–C42–C47	121.9(5)
C43–C42–C47	117.8(5)	C42–C43–H43	119.5
C42–C43–C44	121.0(6)	H43–C43–C44	119.5
C43–C44–H44	119.9	C43–C44–C45	120.1(7)
H44–C44–C45	119.9	C44–C45–H45	120.0
C44–C45–C46	120.0(6)	H45–C45–C46	120.0
C45–C46–H46	120.0	C45–C46–C47	120.0(6)
H46–C46–C47	120.0	C42–C47–C46	121.0(7)
C42–C47–H47	119.5	C46–C47–H47	119.5
P3–C48–C49	120.7(4)	P3–C48–C53	121.3(5)
C49–C48–C53	117.9(6)	C48–C49–H49	119.3
C48–C49–C50	121.4(6)	H49–C49–C50	119.3
C49–C50–H50	120.2	C49–C50–C51	119.5(7)
H50–C50–C51	120.2	C50–C51–H51	119.8
C50–C51–C52	120.4(7)	H51–C51–C52	119.8
C51–C52–H52	119.8	C51–C52–C53	120.3(6)
H52–C52–C53	119.8	C48–C53–C52	120.4(7)
C48–C53–H53	119.8	C52–C53–H53	119.8
Re–C54–O1	177.8(5)	Re–C55–O2	178.0(5)

Table 3b:4. Torsion angles [°] for ljh117 (**3b**).

N2–B–N1–C1	175.8(5)	N2–B–N1–C4	−0.9(7)
C18–B–N1–C1	59.3(7)	C18–B–N1–C4	−117.5(6)
C19–B–N1–C1	−68.2(7)	C19–B–N1–C4	115.0(6)
N1–B–N2–C6	−0.2(7)	N1–B–N2–C9	−176.5(5)
C18–B–N2–C6	115.1(6)	C18–B–N2–C9	−61.3(7)
C19–B–N2–C6	−115.6(6)	C19–B–N2–C9	68.0(7)
B–N1–C1–C2	−176.6(5)	B–N1–C1–C10	5.2(9)
C4–N1–C1–C2	0.6(6)	C4–N1–C1–C10	−177.5(6)
N1–C1–C2–C3	−1.2(7)	N1–C1–C2–C11	179.4(5)
C10–C1–C2–C3	177.0(6)	C10–C1–C2–C11	−2.4(10)
C1–C2–C3–C4	1.2(6)	C1–C2–C3–C13	−176.3(6)
C11–C2–C3–C4	−179.4(5)	C11–C2–C3–C13	3.1(10)
B–N1–C4–C3	177.4(5)	B–N1–C4–C5	−0.1(8)
C1–N1–C4–C3	0.1(6)	C1–N1–C4–C5	−177.3(5)
C2–C3–C4–N1	−0.9(6)	C2–C3–C4–C5	176.3(6)
C13–C3–C4–N1	176.6(5)	C13–C3–C4–C5	−6.2(10)
N1–C4–C5–C6	2.4(8)	N1–C4–C5–C20	179.3(5)
C3–C4–C5–C6	−174.5(5)	C3–C4–C5–C20	2.5(9)
C4–C5–C6–N2	−3.5(8)	C4–C5–C6–C7	179.7(5)
C20–C5–C6–N2	179.5(5)	C20–C5–C6–C7	2.7(9)
B–N2–C6–C5	2.3(8)	B–N2–C6–C7	179.7(5)
C9–N2–C6–C5	179.3(5)	C9–N2–C6–C7	−3.3(6)
N2–C6–C7–C8	3.2(6)	N2–C6–C7–C14	−179.5(5)
C5–C6–C7–C8	−179.7(5)	C5–C6–C7–C14	−2.4(10)
C6–C7–C8–C9	−1.9(6)	C6–C7–C8–C15	175.7(5)
C14–C7–C8–C9	−179.4(5)	C14–C7–C8–C15	−1.8(9)
B–N2–C9–C8	179.0(5)	B–N2–C9–C17	−0.3(9)
C6–N2–C9–C8	2.1(6)	C6–N2–C9–C17	−177.2(5)
C7–C8–C9–N2	−0.1(7)	C7–C8–C9–C17	179.2(6)
C15–C8–C9–N2	−177.8(5)	C15–C8–C9–C17	1.5(10)

C1–C2–C11–C12	−82.5(9)	C3–C2–C11–C12	98.2(8)
C7–C8–C15–C16	−78.2(8)	C9–C8–C15–C16	99.1(8)
C4–C5–C20–C21	−98.6(7)	C4–C5–C20–C25	83.5(7)
C6–C5–C20–C21	78.4(7)	C6–C5–C20–C25	−99.4(6)
C5–C20–C21–C22	−172.7(6)	C25–C20–C21–C22	5.3(9)
C20–C21–C22–C23	−0.4(9)	C21–C22–C23–C24	−4.6(8)
C21–C22–C23–P2	179.9(4)	C22–C23–C24–C25	4.9(8)
P2–C23–C24–C25	−179.8(4)	C23–C24–C25–C20	0.0(9)
C5–C20–C25–C24	172.9(5)	C21–C20–C25–C24	−5.0(9)
C22–C23–P2–Re	−19.0(5)	C22–C23–P2–C39	100.6(5)
C22–C23–P2–C40	−142.0(4)	C24–C23–P2–Re	165.8(4)
C24–C23–P2–C39	−74.6(5)	C24–C23–P2–C40	42.8(5)
Re–P1–C26–C27	125.1(5)	Re–P1–C26–C31	−55.2(5)
C32–P1–C26–C27	−101.7(5)	C32–P1–C26–C31	78.1(5)
C38–P1–C26–C27	4.3(6)	C38–P1–C26–C31	−176.0(5)
P1–C26–C27–C28	177.9(5)	C31–C26–C27–C28	−1.8(9)
C26–C27–C28–C29	0.9(10)	C27–C28–C29–C30	0.0(10)
C28–C29–C30–C31	0.1(10)	P1–C26–C31–C30	−177.9(5)
C27–C26–C31–C30	1.8(9)	C29–C30–C31–C26	−1.0(10)
Re–P1–C32–C33	−167.2(3)	Re–P1–C32–C37	13.7(5)
C26–P1–C32–C33	61.0(5)	C26–P1–C32–C37	−118.1(5)
C38–P1–C32–C33	−46.3(5)	C38–P1–C32–C37	134.6(5)
P1–C32–C33–C34	−179.7(4)	C37–C32–C33–C34	−0.6(8)
C32–C33–C34–C35	1.0(9)	C33–C34–C35–C36	−1.3(9)
C34–C35–C36–C37	1.0(9)	P1–C32–C37–C36	179.5(4)
C33–C32–C37–C36	0.4(8)	C35–C36–C37–C32	−0.6(9)
Re–P1–C38–C39	26.7(4)	C26–P1–C38–C39	153.9(4)
C32–P1–C38–C39	−102.3(4)	P1–C38–C39–P2	−52.8(5)
Re–P2–C39–C38	56.0(4)	C23–P2–C39–C38	−76.3(4)
C40–P2–C39–C38	171.1(4)	Re–P2–C40–C41	−53.5(4)
C23–P2–C40–C41	81.0(4)	C39–P2–C40–C41	−168.7(4)
P2–C40–C41–P3	52.4(5)	Re–P3–C41–C40	−27.6(4)

C42–P3–C41–C40	−156.0(4)	C48–P3–C41–C40	97.8(4)
Re–P3–C42–C43	−65.1(5)	Re–P3–C42–C47	111.4(4)
C41–P3–C42–C43	53.9(5)	C41–P3–C42–C47	−129.7(5)
C48–P3–C42–C43	161.0(5)	C48–P3–C42–C47	−22.5(5)
P3–C42–C43–C44	179.2(4)	C47–C42–C43–C44	2.6(8)
C42–C43–C44–C45	−1.0(9)	C43–C44–C45–C46	−1.6(9)
C44–C45–C46–C47	2.5(9)	C45–C46–C47–C42	−0.8(9)
P3–C42–C47–C46	−178.3(4)	C43–C42–C47–C46	−1.7(8)
Re–P3–C48–C49	−26.4(5)	Re–P3–C48–C53	156.5(4)
C41–P3–C48–C49	−145.3(5)	C41–P3–C48–C53	37.5(5)
C42–P3–C48–C49	109.8(5)	C42–P3–C48–C53	−67.3(5)
P3–C48–C49–C50	−178.6(4)	C53–C48–C49–C50	−1.4(9)
C48–C49–C50–C51	−0.7(9)	C49–C50–C51–C52	1.4(10)
C50–C51–C52–C53	−0.1(10)	C51–C52–C53–C48	−2.0(9)
P3–C48–C53–C52	179.9(4)	C49–C48–C53–C52	2.7(8)

Table 3b:5. Anisotropic displacement parameters (Å^2) for ljh117 (**3b**). The anisotropic displacement factor exponent takes the form: $-2\pi^2[h^2a^{*2}U^{11} + ... + 2hka^*b^*U^{12}]$

	U^{11}	U^{22}	U^{33}	U^{23}	U^{13}	U^{12}
Re	0.02648(14)	0.02370(15)	0.01895(13)	−0.00037(11)	0.00415(9)	0.00130(12)
B	0.041(4)	0.030(4)	0.038(4)	−0.004(3)	0.010(3)	−0.008(4)
N1	0.043(3)	0.032(3)	0.034(3)	−0.008(2)	0.011(2)	−0.006(3)
N2	0.042(3)	0.026(3)	0.036(3)	−0.003(2)	0.012(2)	0.001(2)
C1	0.045(4)	0.043(4)	0.040(3)	−0.014(3)	0.014(3)	−0.006(3)
C2	0.042(4)	0.059(5)	0.030(3)	−0.012(3)	0.013(3)	−0.008(4)
C3	0.044(4)	0.046(4)	0.029(3)	−0.004(3)	0.010(3)	0.003(3)
C4	0.037(3)	0.034(4)	0.027(3)	−0.006(3)	0.007(3)	−0.003(3)
C5	0.048(4)	0.030(4)	0.024(3)	−0.006(3)	0.013(3)	−0.003(3)
C6	0.032(3)	0.015(3)	0.026(3)	0.003(2)	0.001(2)	−0.005(3)
C7	0.041(3)	0.029(4)	0.031(3)	0.000(3)	0.012(3)	0.000(3)
C8	0.042(4)	0.035(4)	0.036(3)	0.001(3)	0.012(3)	0.000(3)
C9	0.044(4)	0.035(4)	0.034(3)	0.001(3)	0.013(3)	0.002(3)
C10	0.050(4)	0.060(5)	0.056(4)	−0.021(4)	0.015(3)	−0.018(4)
C11	0.041(4)	0.077(6)	0.037(3)	−0.010(4)	0.008(3)	−0.012(4)
C12	0.050(4)	0.103(7)	0.048(4)	−0.029(4)	0.014(3)	0.000(5)
C13	0.041(4)	0.065(5)	0.035(3)	−0.001(3)	0.003(3)	0.007(4)
C14	0.038(3)	0.042(4)	0.041(3)	0.002(3)	−0.003(3)	−0.015(3)
C15	0.034(3)	0.052(5)	0.041(3)	0.003(3)	0.004(3)	0.001(3)
C16	0.043(4)	0.067(5)	0.042(4)	−0.002(4)	0.002(3)	−0.005(4)
C17	0.059(4)	0.041(5)	0.058(4)	0.013(4)	0.011(4)	0.008(4)
C18	0.059(4)	0.038(4)	0.054(4)	0.003(3)	0.022(3)	−0.006(4)
C19	0.048(4)	0.039(4)	0.053(4)	−0.006(3)	0.020(3)	−0.005(3)
C20	0.034(3)	0.030(4)	0.027(3)	0.001(3)	0.003(2)	0.004(3)
C21	0.047(4)	0.031(4)	0.032(3)	0.001(3)	0.016(3)	−0.002(3)
C22	0.047(3)	0.032(4)	0.026(3)	−0.008(3)	0.015(3)	−0.002(3)
C23	0.030(3)	0.032(4)	0.020(3)	0.003(2)	0.004(2)	0.004(3)

C24	0.041(3)	0.038(4)	0.023(3)	0.000(3)	0.010(2)	0.009(3)
C25	0.051(4)	0.023(3)	0.028(3)	–0.004(3)	0.013(3)	0.007(3)
P1	0.0282(7)	0.0224(8)	0.0214(7)	–0.0027(6)	0.0026(5)	0.0008(6)
P2	0.0299(7)	0.0236(8)	0.0189(7)	–0.0005(6)	0.0048(5)	0.0032(6)
P3	0.0271(7)	0.0295(9)	0.0240(7)	0.0004(6)	0.0040(6)	0.0039(6)
C26	0.027(3)	0.029(3)	0.027(3)	0.005(3)	0.005(2)	0.000(3)
C27	0.041(3)	0.046(4)	0.035(3)	–0.006(3)	0.008(3)	–0.005(3)
C28	0.044(4)	0.039(4)	0.046(4)	0.000(3)	0.019(3)	–0.014(3)
C29	0.041(4)	0.046(4)	0.039(3)	0.018(3)	0.017(3)	0.006(3)
C30	0.050(4)	0.045(4)	0.035(3)	–0.004(3)	0.019(3)	–0.004(3)
C31	0.043(4)	0.039(4)	0.039(3)	–0.008(3)	0.015(3)	–0.010(3)
C32	0.027(3)	0.031(4)	0.023(3)	0.009(2)	0.009(2)	0.010(3)
C33	0.034(3)	0.034(4)	0.042(3)	0.001(3)	0.010(3)	0.006(3)
C34	0.041(4)	0.056(5)	0.033(3)	0.004(3)	0.005(3)	0.017(4)
C35	0.047(4)	0.051(5)	0.043(4)	0.015(3)	0.016(3)	0.021(4)
C36	0.053(4)	0.027(4)	0.064(4)	0.006(3)	0.029(4)	0.006(3)
C37	0.037(3)	0.033(4)	0.041(3)	0.001(3)	0.015(3)	0.004(3)
C38	0.031(3)	0.037(4)	0.023(3)	0.001(3)	0.001(2)	0.001(3)
C39	0.031(3)	0.032(4)	0.018(3)	–0.001(2)	0.002(2)	0.003(3)
C40	0.035(3)	0.028(3)	0.019(3)	0.001(2)	0.004(2)	0.000(3)
C41	0.030(3)	0.037(4)	0.027(3)	–0.001(3)	0.003(2)	0.005(3)
C42	0.030(3)	0.034(4)	0.027(3)	0.002(3)	0.008(2)	0.009(3)
C43	0.038(3)	0.030(4)	0.038(3)	–0.002(3)	0.006(3)	0.007(3)
C44	0.055(4)	0.038(4)	0.042(4)	0.007(3)	0.012(3)	0.014(3)
C45	0.046(4)	0.053(5)	0.031(3)	0.013(3)	0.010(3)	0.027(4)
C46	0.044(4)	0.062(5)	0.024(3)	–0.003(3)	–0.001(3)	0.008(4)
C47	0.039(3)	0.044(4)	0.028(3)	0.000(3)	0.006(3)	0.011(3)
C48	0.030(3)	0.041(4)	0.023(3)	0.004(3)	0.004(2)	0.000(3)
C49	0.034(3)	0.032(4)	0.044(4)	–0.002(3)	0.009(3)	–0.005(3)
C50	0.044(4)	0.040(4)	0.048(4)	0.003(3)	0.003(3)	0.002(3)
C51	0.045(4)	0.048(5)	0.048(4)	0.001(3)	0.005(3)	–0.014(4)
C52	0.036(4)	0.065(5)	0.051(4)	0.000(4)	0.019(3)	–0.013(4)
C53	0.032(3)	0.050(5)	0.038(3)	0.001(3)	0.007(3)	0.000(3)

C54	0.031(3)	0.020(3)	0.031(3)	−0.006(3)	0.005(2)	−0.005(3)
O1	0.039(2)	0.044(3)	0.044(2)	0.012(2)	0.010(2)	0.005(2)
C55	0.033(3)	0.031(4)	0.028(3)	0.002(3)	0.006(2)	0.006(3)
O2	0.057(3)	0.041(3)	0.037(2)	−0.012(2)	0.015(2)	−0.004(2)
Cl	0.0423(8)	0.0332(8)	0.0222(6)	0.0022(6)	0.0079(6)	0.0011(7)

Table 3b:6. Hydrogen coordinates and isotropic displacement parameters (Å^2) for ljh117 (**3b**).

	x	y	z	U
H10A	0.4785	0.6323	0.5962	0.083
H10B	0.5935	0.6143	0.6614	0.083
H10C	0.4960	0.6302	0.7017	0.083
H11A	0.6579	0.4928	0.7965	0.063
H11B	0.6539	0.5539	0.7889	0.063
H12A	0.8087	0.5264	0.7526	0.100
H12B	0.7250	0.5472	0.6624	0.100
H12C	0.7388	0.4869	0.6804	0.100
H13A	0.5761	0.4224	0.7426	0.073
H13B	0.4849	0.3979	0.6603	0.073
H13C	0.4536	0.4155	0.7481	0.073
H14A	–0.0223	0.4096	0.3994	0.065
H14B	0.0679	0.3962	0.4910	0.065
H14C	0.0962	0.3902	0.3981	0.065
H15A	–0.0992	0.5341	0.3000	0.053
H15B	–0.1163	0.4773	0.3319	0.053
H16A	–0.1233	0.4778	0.1801	0.079
H16B	–0.0221	0.4439	0.2352	0.079
H16C	–0.0020	0.5007	0.2044	0.079
H17A	0.1254	0.6261	0.4008	0.081
H17B	–0.0011	0.6103	0.3722	0.081
H17C	0.0718	0.5997	0.3059	0.081
H18A	0.3850	0.5748	0.4313	0.074
H18B	0.3905	0.6302	0.4762	0.074
H18C	0.2849	0.6138	0.3978	0.074
H19A	0.2077	0.6002	0.6218	0.069
H19B	0.1819	0.6395	0.5398	0.069
H19C	0.2978	0.6412	0.6141	0.069

H21	0.3307	0.3858	0.4698	0.043
H22	0.3036	0.2988	0.4811	0.040
H24	0.1998	0.3245	0.6993	0.040
H25	0.2264	0.4119	0.6866	0.040
H27	0.5733	0.2566	0.5644	0.049
H28	0.6811	0.2844	0.4764	0.050
H29	0.6633	0.2479	0.3372	0.049
H30	0.5371	0.1827	0.2868	0.050
H31	0.4272	0.1540	0.3739	0.048
H33	0.6225	0.1514	0.6546	0.045
H34	0.7213	0.0780	0.7074	0.054
H35	0.6438	−0.0030	0.6680	0.056
H36	0.4628	−0.0091	0.5797	0.054
H37	0.3629	0.0659	0.5272	0.043
H38A	0.5015	0.2020	0.6918	0.038
H38B	0.4513	0.2507	0.6319	0.038
H39A	0.3527	0.2412	0.7375	0.034
H39B	0.3289	0.1818	0.7117	0.034
H40A	0.0898	0.1939	0.6543	0.034
H40B	0.0882	0.2547	0.6701	0.034
H41A	−0.0013	0.2693	0.5186	0.039
H41B	−0.0700	0.2263	0.5522	0.039
H43	0.0177	0.2909	0.3809	0.043
H44	−0.0806	0.3344	0.2529	0.054
H45	−0.2173	0.2926	0.1451	0.052
H46	−0.2506	0.2061	0.1615	0.055
H47	−0.1561	0.1629	0.2908	0.046
H49	0.0261	0.0799	0.4274	0.045
H50	−0.0868	0.0106	0.4307	0.055
H51	−0.2504	0.0233	0.4657	0.059
H52	−0.3041	0.1049	0.4920	0.059
H53	−0.1950	0.1753	0.4835	0.049

5.3 Crystallographic data for *cis,fac*-[ReCl(CO)$_2$(2)](3c)

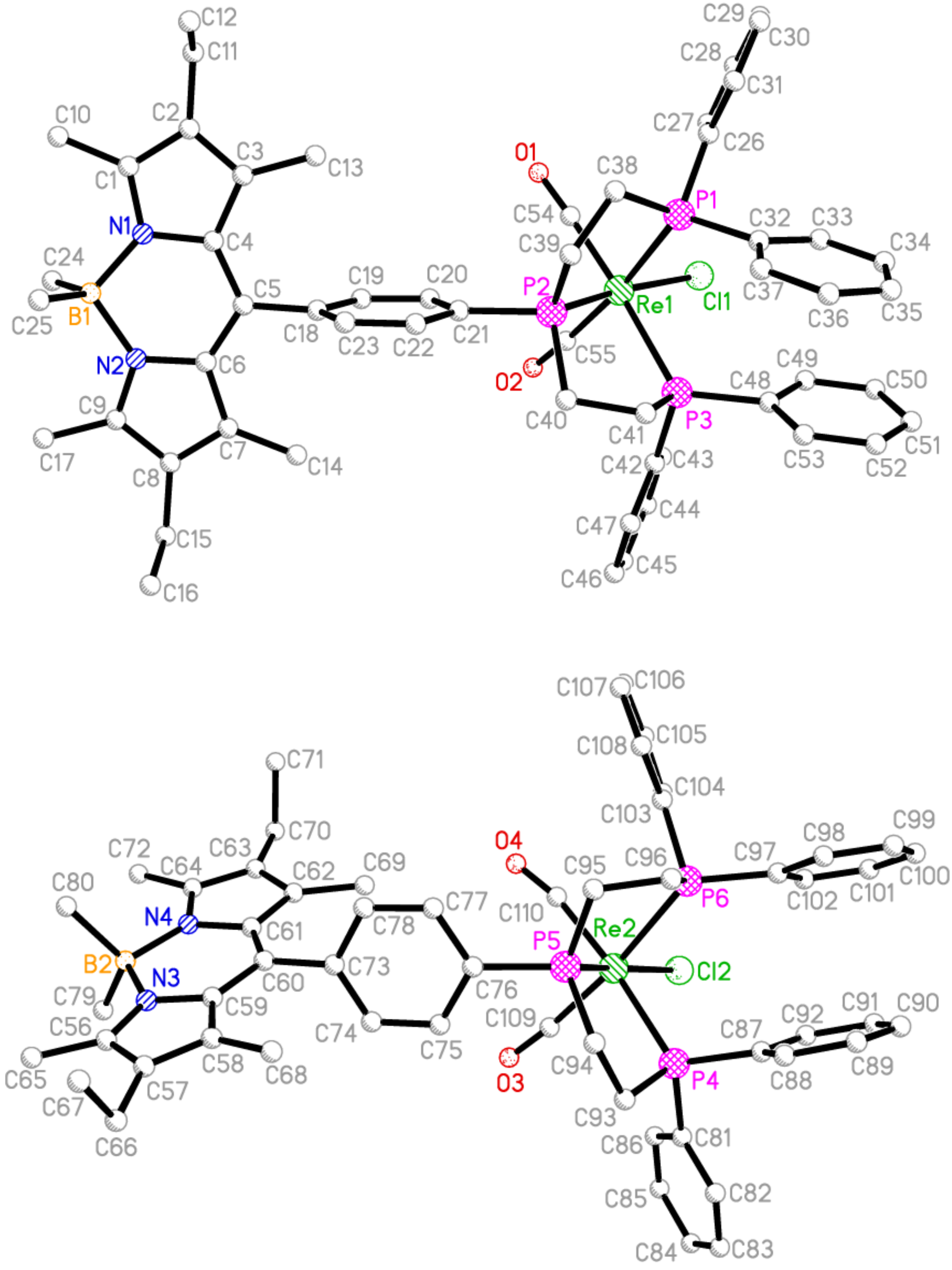

Table 3c:1. Crystal data and structure refinement for ljh102 (**3c**).

Identification code	ljh102 (**3c**)	
Chemical formula (moiety)	$C_{55}H_{60}BClN_2O_2P_3Re\cdot 2C_4H_8O$	
Chemical formula (total)	$C_{63}H_{76}BClN_2O_4P_3Re$	
Formula weight	1250.62	
Temperature	150(2) K	
Radiation, wavelength	CuKα, 1.54178 Å	
Crystal system, space group	triclinic, $P\overline{1}$	
Unit cell parameters	a = 12.0578(8) Å	α = 84.193(5)°
	b = 15.8145(12) Å	β = 89.183(5)°
	c = 32.8027(16) Å	γ = 89.699(6)°
Cell volume	6222.3(7) $Å^3$	
Z	4	
Calculated density	1.335 g/cm^3	
Absorption coefficient μ	5.297 mm^{-1}	
F(000)	2568	
Crystal colour and size	red, 0.20 × 0.02 × 0.02 mm^3	
Reflections for cell refinement	9471 (θ range 2.7 to 62.3°)	
Data collection method	Agilent Technologies Gemini A Ultra	
	ω scans	
θ range for data collection	2.7 to 62.4°	
Index ranges	h −13 to 13, k −18 to 14, l −37 to 35	
Completeness to θ = 67.7°	82.2 %	
Reflections collected	40658	
Independent reflections	18503 (R_{int} = 0.0673)	
Reflections with F^2>2σ	13764	
Absorption correction	multi-scan	
Min. and max. transmission	0.420 and 0.900	
Structure solution	direct methods	
Refinement method	Full-matrix least-squares on F^2	
Weighting parameters a, b	0.0914, 37.8105	
Data / restraints / parameters	18503 / 2512 / 1351	

Final R indices [$F^2 > 2\sigma$]	R1 = 0.0664, wR2 = 0.1725
R indices (all data)	R1 = 0.0910, wR2 = 0.1927
Goodness-of-fit on F^2	1.053
Largest and mean shift/su	0.001 and 0.000
Largest diff. peak and hole	3.22 and −2.00 e Å^{-3}

Table 3c:2. Atomic coordinates and equivalent isotropic displacement parameters (Å^2) for ljh102 (3c). U_{eq} is defined as one third of the trace of the orthogonalised U^{ij} tensor.

	x	y	z	U_{eq}
Re1	0.18741(3)	0.95307(2)	0.64733(2)	0.02319(13)
B1	0.6244(13)	0.8872(12)	0.9291(4)	0.066(3)
N1	0.4963(8)	0.8856(6)	0.9199(3)	0.049(2)
N2	0.6837(8)	0.9048(6)	0.8853(3)	0.050(2)
C1	0.4122(10)	0.8685(9)	0.9473(4)	0.057(3)
C2	0.3073(10)	0.8825(9)	0.9279(4)	0.057(3)
C3	0.3278(9)	0.9094(7)	0.8868(3)	0.045(2)
C4	0.4466(9)	0.9111(7)	0.8820(3)	0.044(2)
C5	0.5135(9)	0.9296(7)	0.8470(3)	0.042(2)
C6	0.6294(9)	0.9242(7)	0.8483(3)	0.044(2)
C7	0.7093(10)	0.9374(8)	0.8160(3)	0.050(3)
C8	0.8111(10)	0.9250(9)	0.8349(4)	0.060(3)
C9	0.7916(10)	0.9071(8)	0.8769(4)	0.054(3)
C10	0.4290(13)	0.8416(11)	0.9919(4)	0.079(4)
C11	0.1967(11)	0.8714(10)	0.9484(4)	0.068(3)
C12	0.1462(11)	0.9533(11)	0.9613(4)	0.076(4)
C13	0.2390(10)	0.9310(9)	0.8564(4)	0.057(3)
C14	0.6923(10)	0.9559(8)	0.7712(3)	0.053(3)
C15	0.9256(11)	0.9343(10)	0.8142(4)	0.073(4)
C16	0.9735(12)	1.0218(11)	0.8136(4)	0.081(4)
C17	0.8797(11)	0.8929(11)	0.9089(4)	0.073(4)
C18	0.4571(9)	0.9575(7)	0.8080(3)	0.040(2)
C19	0.4197(9)	0.8988(7)	0.7827(3)	0.042(2)
C20	0.3594(8)	0.9241(6)	0.7475(3)	0.035(2)
C21	0.3365(8)	1.0089(6)	0.7371(3)	0.0297(18)
C22	0.3759(9)	1.0681(6)	0.7623(3)	0.038(2)
C23	0.4326(9)	1.0428(7)	0.7973(3)	0.043(2)
C24	0.6630(13)	0.7964(11)	0.9505(5)	0.093(5)
C25	0.6479(14)	0.9638(12)	0.9554(5)	0.091(4)

P1	0.01922(19)	1.03949(14)	0.65358(7)	0.0270(5)
P2	0.25908(19)	1.04438(14)	0.69081(7)	0.0268(5)
P3	0.27522(18)	1.05689(13)	0.59472(7)	0.0229(5)
C26	−0.1172(8)	0.9896(6)	0.6580(3)	0.0291(18)
C27	−0.1300(9)	0.9056(7)	0.6496(4)	0.047(3)
C28	−0.2369(10)	0.8708(8)	0.6501(4)	0.059(3)
C29	−0.3278(10)	0.9169(8)	0.6597(4)	0.055(3)
C30	−0.3147(9)	0.9988(8)	0.6699(3)	0.047(2)
C31	−0.2110(8)	1.0344(7)	0.6686(3)	0.039(2)
C32	−0.0143(7)	1.1293(5)	0.6158(3)	0.0271(17)
C33	−0.0645(8)	1.1111(6)	0.5798(3)	0.033(2)
C34	−0.0875(8)	1.1753(6)	0.5492(3)	0.038(2)
C35	−0.0585(9)	1.2573(7)	0.5537(3)	0.043(2)
C36	−0.0069(9)	1.2767(7)	0.5888(3)	0.041(2)
C37	0.0159(8)	1.2130(6)	0.6203(3)	0.033(2)
C38	0.0343(8)	1.0865(6)	0.7028(3)	0.0312(19)
C39	0.1527(8)	1.1175(6)	0.7097(3)	0.0306(19)
C40	0.3592(8)	1.1172(6)	0.6637(3)	0.0288(19)
C41	0.3118(8)	1.1483(6)	0.6213(3)	0.0277(18)
C42	0.4072(7)	1.0190(5)	0.5747(3)	0.0265(17)
C43	0.4088(9)	0.9429(6)	0.5586(3)	0.035(2)
C44	0.5060(9)	0.9115(7)	0.5430(3)	0.041(2)
C45	0.6034(9)	0.9555(7)	0.5439(3)	0.041(2)
C46	0.6053(9)	1.0336(7)	0.5600(3)	0.044(2)
C47	0.5068(8)	1.0643(7)	0.5750(3)	0.039(2)
C48	0.2079(7)	1.1026(5)	0.5477(3)	0.0254(17)
C49	0.1542(7)	1.0485(6)	0.5237(3)	0.0296(19)
C50	0.1058(8)	1.0821(6)	0.4868(3)	0.0318(19)
C51	0.1132(8)	1.1679(6)	0.4738(3)	0.0312(19)
C52	0.1658(8)	1.2210(6)	0.4983(3)	0.0310(19)
C53	0.2137(8)	1.1884(6)	0.5345(3)	0.0303(19)
C54	0.1260(7)	0.8801(6)	0.6918(3)	0.0273(18)
O1	0.0925(7)	0.8343(5)	0.7197(2)	0.053(2)

C55	0.3196(7)	0.8842(5)	0.6495(3)	0.0276(18)
O2	0.3985(6)	0.8407(4)	0.6530(2)	0.0405(16)
Cl1	0.10684(18)	0.87199(13)	0.59292(7)	0.0305(5)
Re2	0.27161(3)	0.45218(2)	0.64411(2)	0.02525(13)
B2	−0.0986(16)	0.2441(12)	0.9088(5)	0.075(3)
N3	0.0016(10)	0.3062(7)	0.9147(3)	0.066(3)
N4	−0.1224(10)	0.2513(7)	0.8616(3)	0.063(3)
C56	0.0479(14)	0.3224(10)	0.9497(4)	0.075(4)
C57	0.1302(14)	0.3845(10)	0.9424(4)	0.076(4)
C58	0.1323(12)	0.4089(8)	0.9007(4)	0.061(3)
C59	0.0525(11)	0.3589(8)	0.8828(4)	0.056(3)
C60	0.0207(11)	0.3547(7)	0.8415(3)	0.052(3)
C61	−0.0613(11)	0.3014(8)	0.8313(4)	0.057(3)
C62	−0.1024(12)	0.2853(8)	0.7919(4)	0.063(3)
C63	−0.1888(14)	0.2309(9)	0.7997(5)	0.076(4)
C64	−0.1991(14)	0.2117(10)	0.8427(5)	0.076(4)
C65	0.0191(16)	0.2800(12)	0.9914(5)	0.101(6)
C66	0.2051(18)	0.4154(12)	0.9741(5)	0.103(5)
C67	0.151(3)	0.4899(19)	0.9924(9)	0.181(11)
C68	0.2074(13)	0.4775(9)	0.8807(4)	0.073(4)
C69	−0.0615(14)	0.3176(8)	0.7501(4)	0.070(4)
C70	−0.2620(16)	0.1930(12)	0.7688(5)	0.097(5)
C71	−0.3630(18)	0.2469(14)	0.7564(6)	0.111(6)
C72	−0.2822(14)	0.1492(11)	0.8639(5)	0.089(5)
C73	0.0855(10)	0.4059(7)	0.8091(3)	0.046(2)
C74	0.1907(10)	0.3827(7)	0.7986(3)	0.048(3)
C75	0.2486(9)	0.4281(6)	0.7666(3)	0.039(2)
C76	0.1982(8)	0.4984(6)	0.7438(3)	0.035(2)
C77	0.0920(9)	0.5227(7)	0.7557(3)	0.044(2)
C78	0.0372(10)	0.4784(7)	0.7883(3)	0.047(3)
C79	−0.0646(17)	0.1485(11)	0.9238(5)	0.101(5)
C80	−0.2073(15)	0.2793(12)	0.9337(5)	0.098(5)
P4	0.46850(18)	0.48548(13)	0.64429(7)	0.0250(5)

P5	0.2645(2)	0.54030(14)	0.69596(7)	0.0290(5)
P6	0.21442(19)	0.58539(14)	0.60251(7)	0.0266(5)
C81	0.5730(8)	0.4009(5)	0.6420(3)	0.0283(18)
C82	0.6849(8)	0.4207(6)	0.6435(3)	0.038(2)
C83	0.7651(9)	0.3572(6)	0.6426(4)	0.044(2)
C84	0.7330(8)	0.2747(6)	0.6406(3)	0.038(2)
C85	0.6214(8)	0.2542(6)	0.6387(3)	0.035(2)
C86	0.5417(8)	0.3172(6)	0.6393(3)	0.0305(19)
C87	0.5212(7)	0.5682(5)	0.6064(3)	0.0258(17)
C88	0.5546(8)	0.6472(6)	0.6155(3)	0.034(2)
C89	0.5891(8)	0.7075(6)	0.5838(3)	0.036(2)
C90	0.5914(8)	0.6889(6)	0.5446(3)	0.037(2)
C91	0.5586(8)	0.6090(6)	0.5350(3)	0.038(2)
C92	0.5217(7)	0.5484(6)	0.5658(3)	0.0310(19)
C93	0.4973(7)	0.5192(6)	0.6950(3)	0.0305(19)
C94	0.4065(8)	0.5763(6)	0.7100(3)	0.0296(19)
C95	0.1919(8)	0.6395(6)	0.6793(3)	0.033(2)
C96	0.2289(7)	0.6711(5)	0.6361(3)	0.0252(17)
C97	0.2686(8)	0.6292(6)	0.5525(3)	0.0311(19)
C98	0.2942(8)	0.7155(6)	0.5443(3)	0.0309(19)
C99	0.3284(8)	0.7495(6)	0.5056(3)	0.036(2)
C100	0.3386(8)	0.6956(7)	0.4744(3)	0.039(2)
C101	0.3128(8)	0.6106(7)	0.4824(3)	0.038(2)
C102	0.2794(8)	0.5778(6)	0.5204(3)	0.0323(19)
C103	0.0672(8)	0.5839(6)	0.5906(3)	0.0318(19)
C104	0.0305(8)	0.5175(6)	0.5686(3)	0.037(2)
C105	−0.0804(8)	0.5111(7)	0.5590(4)	0.043(2)
C106	−0.1573(9)	0.5688(6)	0.5718(4)	0.045(2)
C107	−0.1220(9)	0.6329(7)	0.5944(4)	0.045(2)
C108	−0.0100(8)	0.6411(6)	0.6028(3)	0.036(2)
C109	0.3017(7)	0.3530(5)	0.6819(3)	0.0288(19)
O3	0.3113(7)	0.2963(4)	0.7057(2)	0.0447(18)
C110	0.1133(7)	0.4328(6)	0.6532(2)	0.0241(18)

O4	0.0265(6)	0.4136(5)	0.6646(2)	0.0472(19)
Cl2	0.29772(19)	0.37754(13)	0.58087(7)	0.0331(5)
O5	0.7064(10)	0.6617(8)	0.7054(4)	0.105(3)
C111	0.8177(15)	0.6364(14)	0.7097(7)	0.130(6)
C112	0.8637(15)	0.6838(14)	0.7436(7)	0.125(6)
C113	0.7800(15)	0.7417(13)	0.7535(7)	0.120(6)
C114	0.6701(14)	0.7124(12)	0.7359(6)	0.108(5)
O6	0.3453(16)	1.2127(11)	0.8502(5)	0.146(5)
C115	0.268(2)	1.1729(18)	0.8788(7)	0.167(8)
C116	0.325(3)	1.168(2)	0.9200(6)	0.192(10)
C117	0.418(3)	1.221(2)	0.9153(6)	0.217(11)
C118	0.420(3)	1.2604(19)	0.8710(7)	0.187(10)
O7	−0.420(3)	0.455(3)	0.8112(16)	0.388(19)
C119	−0.317(5)	0.483(4)	0.7927(11)	0.39(2)
C120	−0.251(4)	0.514(4)	0.828(2)	0.39(2)
C121	−0.288(5)	0.463(5)	0.8633(14)	0.40(2)
C122	−0.395(5)	0.418(4)	0.8514(16)	0.40(2)
O8	0.489(4)	0.666(5)	0.858(4)	0.61(3)
C123	0.403(11)	0.657(6)	0.8880(13)	0.61(3)
C124	0.298(6)	0.693(6)	0.865(4)	0.61(3)
C125	0.316(9)	0.684(6)	0.823(3)	0.59(3)
C126	0.442(10)	0.667(6)	0.818(2)	0.59(3)

Table 3c:3. Bond lengths [Å] and angles [°] for ljh102 (**3c**).

Re1–P1	2.457(2)	Re1–P2	2.309(2)
Re1–P3	2.488(2)	Re1–C54	1.908(9)
Re1–C55	1.925(9)	Re1–Cl1	2.510(2)
B1–N1	1.578(18)	B1–N2	1.594(17)
B1–C24	1.60(2)	B1–C25	1.59(2)
N1–C1	1.356(14)	N1–C4	1.410(14)
N2–C6	1.395(14)	N2–C9	1.326(15)
C1–C2	1.431(18)	C1–C10	1.498(17)
C2–C3	1.389(16)	C2–C11	1.489(17)
C3–C4	1.439(16)	C3–C13	1.488(16)
C4–C5	1.399(15)	C5–C6	1.400(16)
C5–C18	1.484(15)	C6–C7	1.422(15)
C7–C8	1.388(17)	C7–C14	1.484(16)
C8–C9	1.394(17)	C8–C15	1.532(18)
C9–C17	1.504(17)	C10–H10A	0.980
C10–H10B	0.980	C10–H10C	0.980
C11–H11A	0.990	C11–H11B	0.990
C11–C12	1.52(2)	C12–H12A	0.980
C12–H12B	0.980	C12–H12C	0.980
C13–H13A	0.980	C13–H13B	0.980
C13–H13C	0.980	C14–H14A	0.980
C14–H14B	0.980	C14–H14C	0.980
C15–H15A	0.990	C15–H15B	0.990
C15–C16	1.50(2)	C16–H16A	0.980
C16–H16B	0.980	C16–H16C	0.980
C17–H17A	0.980	C17–H17B	0.980
C17–H17C	0.980	C18–C19	1.387(16)
C18–C23	1.390(15)	C19–H19	0.950
C19–C20	1.398(14)	C20–H20	0.950
C20–C21	1.378(13)	C21–C22	1.398(14)
C21–P2	1.832(9)	C22–H22	0.950
C22–C23	1.368(15)	C23–H23	0.950

C24–H24A	0.980	C24–H24B	0.980
C24–H24C	0.980	C25–H25A	0.980
C25–H25B	0.980	C25–H25C	0.980
P1–C26	1.826(10)	P1–C32	1.835(9)
P1–C38	1.856(10)	P2–C39	1.863(9)
P2–C40	1.831(9)	P3–C41	1.821(9)
P3–C42	1.834(9)	P3–C48	1.833(9)
C26–C27	1.393(14)	C26–C31	1.390(13)
C27–H27	0.950	C27–C28	1.404(15)
C28–H28	0.950	C28–C29	1.364(17)
C29–H29	0.950	C29–C30	1.381(18)
C30–H30	0.950	C30–C31	1.372(15)
C31–H31	0.950	C32–C33	1.393(14)
C32–C37	1.397(13)	C33–H33C	0.950
C33–C34	1.383(14)	C34–H34	0.950
C34–C35	1.367(15)	C35–H35	0.950
C35–C36	1.379(17)	C36–H36	0.950
C36–C37	1.398(14)	C37–H37	0.950
C38–H38A	0.990	C38–H38B	0.990
C38–C39	1.538(13)	C39–H39A	0.990
C39–H39B	0.990	C40–H40A	0.990
C40–H40B	0.990	C40–C41	1.545(13)
C41–H41A	0.990	C41–H41B	0.990
C42–C43	1.362(14)	C42–C47	1.403(14)
C43–H43	0.950	C43–C44	1.382(14)
C44–H44	0.950	C44–C45	1.370(16)
C45–H45	0.950	C45–C46	1.392(16)
C46–H46	0.950	C46–C47	1.384(14)
C47–H47	0.950	C48–C49	1.388(13)
C48–C53	1.384(13)	C49–H49	0.950
C49–C50	1.406(14)	C50–H50	0.950
C50–C51	1.383(13)	C51–H51	0.950
C51–C52	1.381(14)	C52–H52	0.950
C52–C53	1.380(13)	C53–H53	0.950

C54–O1	1.177(11)	C55–O2	1.170(11)
Re2–P4	2.435(2)	Re2–P5	2.306(2)
Re2–P6	2.491(2)	Re2–C109	1.933(9)
Re2–C110	1.949(8)	Re2–Cl2	2.504(2)
B2–N3	1.59(2)	B2–N4	1.57(2)
B2–C79	1.60(3)	B2–C80	1.66(3)
N3–C56	1.334(19)	N3–C59	1.407(14)
N4–C61	1.409(14)	N4–C64	1.315(19)
C56–C57	1.40(2)	C56–C65	1.495(18)
C57–C58	1.382(19)	C57–C66	1.51(2)
C58–C59	1.42(2)	C58–C68	1.509(18)
C59–C60	1.420(18)	C60–C61	1.370(18)
C60–C73	1.483(15)	C61–C62	1.435(19)
C62–C63	1.36(2)	C62–C69	1.494(17)
C63–C64	1.42(2)	C63–C70	1.52(2)
C64–C72	1.521(19)	C65–H65A	0.980
C65–H65B	0.980	C65–H65C	0.980
C66–H66A	0.990	C66–H66B	0.990
C66–C67	1.51(3)	C67–H67A	0.980
C67–H67B	0.980	C67–H67C	0.980
C68–H68A	0.980	C68–H68B	0.980
C68–H68C	0.980	C69–H69A	0.980
C69–H69B	0.980	C69–H69C	0.980
C70–H70A	0.990	C70–H70B	0.990
C70–C71	1.52(3)	C71–H71A	0.980
C71–H71B	0.980	C71–H71C	0.980
C72–H72A	0.980	C72–H72B	0.980
C72–H72C	0.980	C73–C74	1.368(16)
C73–C78	1.403(16)	C74–H74	0.950
C74–C75	1.393(14)	C75–H75	0.950
C75–C76	1.415(14)	C76–C77	1.397(15)
C76–P5	1.816(10)	C77–H77	0.950
C77–C78	1.381(14)	C78–H78	0.950
C79–H79A	0.980	C79–H79B	0.980

C79–H79C	0.980	C80–H80A	0.980
C80–H80B	0.980	C80–H80C	0.980
P4–C81	1.839(9)	P4–C87	1.822(9)
P4–C93	1.837(10)	P5–C94	1.884(9)
P5–C95	1.832(9)	P6–C96	1.843(9)
P6–C97	1.827(9)	P6–C103	1.823(10)
C81–C82	1.389(14)	C81–C86	1.390(13)
C82–H82	0.950	C82–C83	1.392(14)
C83–H83	0.950	C83–C84	1.371(15)
C84–H84	0.950	C84–C85	1.390(15)
C85–H85	0.950	C85–C86	1.381(13)
C86–H86	0.950	C87–C88	1.375(13)
C87–C92	1.399(13)	C88–H88	0.950
C88–C89	1.399(13)	C89–H89	0.950
C89–C90	1.349(15)	C90–H90	0.950
C90–C91	1.393(15)	C91–H91	0.950
C91–C92	1.390(13)	C92–H92	0.950
C93–H93A	0.990	C93–H93B	0.990
C93–C94	1.524(13)	C94–H94A	0.990
C94–H94B	0.990	C95–H95A	0.990
C95–H95B	0.990	C95–C96	1.516(13)
C96–H96A	0.990	C96–H96B	0.990
C97–C98	1.400(13)	C97–C102	1.397(13)
C98–H98	0.950	C98–C99	1.385(13)
C99–H99	0.950	C99–C100	1.400(15)
C100–H100	0.950	C100–C101	1.379(15)
C101–H101	0.950	C101–C102	1.357(14)
C102–H102	0.950	C103–C104	1.410(14)
C103–C108	1.379(13)	C104–H104	0.950
C104–C105	1.384(15)	C105–H105	0.950
C105–C106	1.387(15)	C106–H106	0.950
C106–C107	1.388(16)	C107–H107	0.950
C107–C108	1.391(15)	C108–H108	0.950
C109–O3	1.134(11)	C110–O4	1.139(10)

O5–C111	1.404(16)	O5–C114	1.409(15)
C111–H11C	0.990	C111–H11D	0.990
C111–C112	1.515(19)	C112–H11E	0.990
C112–H11F	0.990	C112–C113	1.42(2)
C113–H11G	0.990	C113–H11H	0.990
C113–C114	1.545(18)	C114–H11I	0.990
C114–H11J	0.990	O6–C115	1.421(17)
O6–C118	1.408(18)	C115–H11K	0.990
C115–H11L	0.990	C115–C116	1.52(2)
C116–H11M	0.990	C116–H11N	0.990
C116–C117	1.40(2)	C117–H11O	0.990
C117–H11P	0.990	C117–C118	1.519(19)
C118–H11Q	0.990	C118–H11R	0.990
O7–C119	1.431(19)	O7–C122	1.424(19)
C119–H11S	0.990	C119–H11T	0.990
C119–C120	1.55(2)	C120–H12D	0.990
C120–H12E	0.990	C120–C121	1.40(3)
C121–H12F	0.990	C121–H12G	0.990
C121–C122	1.55(2)	C122–H12H	0.990
C122–H12I	0.990	O8–C123	1.42(2)
O8–C126	1.42(2)	C123–H12J	0.990
C123–H12K	0.990	C123–C124	1.55(2)
C124–H12L	0.990	C124–H12M	0.990
C124–C125	1.41(3)	C125–H12N	0.990
C125–H12O	0.990	C125–C126	1.54(2)
C126–H12P	0.990	C126–H12Q	0.990

P1–Re1–P2	82.99(8)	P1–Re1–P3	93.93(7)
P1–Re1–C54	85.6(3)	P1–Re1–C55	173.1(3)
P1–Re1–Cl1	93.40(8)	P2–Re1–P3	81.74(8)
P2–Re1–C54	92.5(3)	P2–Re1–C55	92.6(3)
P2–Re1–Cl1	171.92(7)	P3–Re1–C54	174.2(3)
P3–Re1–C55	90.6(3)	P3–Re1–Cl1	91.32(7)
C54–Re1–C55	89.4(4)	C54–Re1–Cl1	94.5(3)

C55–Re1–Cl1	91.6(3)	N1–B1–N2	105.1(9)
N1–B1–C24	109.8(12)	N1–B1–C25	108.6(13)
N2–B1–C24	109.4(13)	N2–B1–C25	109.5(12)
C24–B1–C25	114.0(13)	B1–N1–C1	127.2(9)
B1–N1–C4	126.0(9)	C1–N1–C4	106.4(9)
B1–N2–C6	125.3(10)	B1–N2–C9	127.8(10)
C6–N2–C9	106.8(9)	N1–C1–C2	110.5(10)
N1–C1–C10	123.8(12)	C2–C1–C10	125.6(11)
C1–C2–C3	107.7(10)	C1–C2–C11	125.7(11)
C3–C2–C11	126.6(12)	C2–C3–C4	105.8(10)
C2–C3–C13	123.8(10)	C4–C3–C13	130.4(10)
N1–C4–C3	109.6(9)	N1–C4–C5	119.5(10)
C3–C4–C5	130.8(10)	C4–C5–C6	122.3(10)
C4–C5–C18	117.4(10)	C6–C5–C18	120.3(9)
N2–C6–C5	120.8(10)	N2–C6–C7	109.4(10)
C5–C6–C7	129.8(10)	C6–C7–C8	104.9(10)
C6–C7–C14	129.4(11)	C8–C7–C14	125.6(11)
C7–C8–C9	108.0(10)	C7–C8–C15	126.6(11)
C9–C8–C15	125.3(12)	N2–C9–C8	110.9(11)
N2–C9–C17	123.8(11)	C8–C9–C17	125.3(11)
C1–C10–H10A	109.5	C1–C10–H10B	109.5
C1–C10–H10C	109.5	H10A–C10–H10B	109.5
H10A–C10–H10C	109.5	H10B–C10–H10C	109.5
C2–C11–H11A	108.7	C2–C11–H11B	108.7
C2–C11–C12	114.2(12)	H11A–C11–H11B	107.6
H11A–C11–C12	108.7	H11B–C11–C12	108.7
C11–C12–H12A	109.5	C11–C12–H12B	109.5
C11–C12–H12C	109.5	H12A–C12–H12B	109.5
H12A–C12–H12C	109.5	H12B–C12–H12C	109.5
C3–C13–H13A	109.5	C3–C13–H13B	109.5
C3–C13–H13C	109.5	H13A–C13–H13B	109.5
H13A–C13–H13C	109.5	H13B–C13–H13C	109.5
C7–C14–H14A	109.5	C7–C14–H14B	109.5
C7–C14–H14C	109.5	H14A–C14–H14B	109.5

H14A–C14–H14C	109.5	H14B–C14–H14C	109.5
C8–C15–H15A	108.8	C8–C15–H15B	108.8
C8–C15–C16	113.6(13)	H15A–C15–H15B	107.7
H15A–C15–C16	108.8	H15B–C15–C16	108.8
C15–C16–H16A	109.5	C15–C16–H16B	109.5
C15–C16–H16C	109.5	H16A–C16–H16B	109.5
H16A–C16–H16C	109.5	H16B–C16–H16C	109.5
C9–C17–H17A	109.5	C9–C17–H17B	109.5
C9–C17–H17C	109.5	H17A–C17–H17B	109.5
H17A–C17–H17C	109.5	H17B–C17–H17C	109.5
C5–C18–C19	121.0(9)	C5–C18–C23	120.9(10)
C19–C18–C23	117.9(9)	C18–C19–H19	119.3
C18–C19–C20	121.4(10)	H19–C19–C20	119.3
C19–C20–H20	120.0	C19–C20–C21	120.0(9)
H20–C20–C21	120.0	C20–C21–C22	118.5(9)
C20–C21–P2	121.1(7)	C22–C21–P2	120.4(7)
C21–C22–H22	119.4	C21–C22–C23	121.2(9)
H22–C22–C23	119.4	C18–C23–C22	120.9(10)
C18–C23–H23	119.5	C22–C23–H23	119.5
B1–C24–H24A	109.5	B1–C24–H24B	109.5
B1–C24–H24C	109.5	H24A–C24–H24B	109.5
H24A–C24–H24C	109.5	H24B–C24–H24C	109.5
B1–C25–H25A	109.5	B1–C25–H25B	109.5
B1–C25–H25C	109.5	H25A–C25–H25B	109.5
H25A–C25–H25C	109.5	H25B–C25–H25C	109.5
Re1–P1–C26	120.5(3)	Re1–P1–C32	121.9(3)
Re1–P1–C38	104.8(3)	C26–P1–C32	98.6(4)
C26–P1–C38	103.9(4)	C32–P1–C38	105.1(4)
Re1–P2–C21	123.7(3)	Re1–P2–C39	112.9(3)
Re1–P2–C40	110.8(3)	C21–P2–C39	103.1(4)
C21–P2–C40	100.7(4)	C39–P2–C40	103.1(4)
Re1–P3–C41	105.9(3)	Re1–P3–C42	113.1(3)
Re1–P3–C48	124.7(3)	C41–P3–C42	104.9(4)
C41–P3–C48	104.6(4)	C42–P3–C48	101.8(4)

P1–C26–C27	120.2(7)	P1–C26–C31	121.4(7)
C27–C26–C31	118.3(9)	C26–C27–H27	120.4
C26–C27–C28	119.1(10)	H27–C27–C28	120.4
C27–C28–H28	119.4	C27–C28–C29	121.3(12)
H28–C28–C29	119.4	C28–C29–H29	120.2
C28–C29–C30	119.6(11)	H29–C29–C30	120.2
C29–C30–H30	120.1	C29–C30–C31	119.8(11)
H30–C30–C31	120.1	C26–C31–C30	121.8(11)
C26–C31–H31	119.1	C30–C31–H31	119.1
P1–C32–C33	117.5(7)	P1–C32–C37	123.1(8)
C33–C32–C37	119.3(9)	C32–C33–H33C	119.7
C32–C33–C34	120.6(9)	H33C–C33–C34	119.7
C33–C34–H34	119.9	C33–C34–C35	120.2(11)
H34–C34–C35	119.9	C34–C35–H35	119.9
C34–C35–C36	120.3(10)	H35–C35–C36	119.9
C35–C36–H36	119.7	C35–C36–C37	120.6(10)
H36–C36–C37	119.7	C32–C37–C36	119.1(10)
C32–C37–H37	120.4	C36–C37–H37	120.4
P1–C38–H38A	108.9	P1–C38–H38B	108.9
P1–C38–C39	113.2(6)	H38A–C38–H38B	107.7
H38A–C38–C39	108.9	H38B–C38–C39	108.9
P2–C39–C38	111.6(6)	P2–C39–H39A	109.3
P2–C39–H39B	109.3	C38–C39–H39A	109.3
C38–C39–H39B	109.3	H39A–C39–H39B	108.0
P2–C40–H40A	110.0	P2–C40–H40B	110.0
P2–C40–C41	108.4(6)	H40A–C40–H40B	108.4
H40A–C40–C41	110.0	H40B–C40–C41	110.0
P3–C41–C40	109.3(6)	P3–C41–H41A	109.8
P3–C41–H41B	109.8	C40–C41–H41A	109.8
C40–C41–H41B	109.8	H41A–C41–H41B	108.3
P3–C42–C43	118.4(7)	P3–C42–C47	123.4(7)
C43–C42–C47	118.2(9)	C42–C43–H43	119.6
C42–C43–C44	120.8(10)	H43–C43–C44	119.6
C43–C44–H44	119.6	C43–C44–C45	120.9(11)

H44–C44–C45	119.6	C44–C45–H45	119.9
C44–C45–C46	120.2(10)	H45–C45–C46	119.9
C45–C46–H46	121.0	C45–C46–C47	118.0(11)
H46–C46–C47	121.0	C42–C47–C46	122.0(10)
C42–C47–H47	119.0	C46–C47–H47	119.0
P3–C48–C49	118.7(7)	P3–C48–C53	122.1(7)
C49–C48–C53	119.2(8)	C48–C49–H49	120.3
C48–C49–C50	119.4(8)	H49–C49–C50	120.3
C49–C50–H50	119.7	C49–C50–C51	120.7(9)
H50–C50–C51	119.7	C50–C51–H51	120.4
C50–C51–C52	119.2(9)	H51–C51–C52	120.4
C51–C52–H52	119.8	C51–C52–C53	120.4(9)
H52–C52–C53	119.8	C48–C53–C52	121.1(9)
C48–C53–H53	119.5	C52–C53–H53	119.5
Re1–C54–O1	177.3(8)	Re1–C55–O2	176.4(8)
P4–Re2–P5	82.76(8)	P4–Re2–P6	95.95(7)
P4–Re2–C109	88.4(3)	P4–Re2–C110	170.1(2)
P4–Re2–Cl2	90.77(8)	P5–Re2–P6	81.76(8)
P5–Re2–C109	92.5(3)	P5–Re2–C110	87.8(3)
P5–Re2–Cl2	169.86(8)	P6–Re2–C109	172.3(3)
P6–Re2–C110	85.4(3)	P6–Re2–Cl2	91.20(7)
C109–Re2–C110	89.2(4)	C109–Re2–Cl2	95.1(3)
C110–Re2–Cl2	99.0(2)	N3–B2–N4	106.6(10)
N3–B2–C79	110.2(15)	N3–B2–C80	107.3(14)
N4–B2–C79	109.0(14)	N4–B2–C80	109.7(14)
C79–B2–C80	113.8(12)	B2–N3–C56	127.6(11)
B2–N3–C59	124.5(11)	C56–N3–C59	107.8(12)
B2–N4–C61	124.9(12)	B2–N4–C64	127.8(11)
C61–N4–C64	107.3(11)	N3–C56–C57	110.5(12)
N3–C56–C65	125.7(14)	C57–C56–C65	123.7(15)
C56–C57–C58	107.1(14)	C56–C57–C66	125.9(13)
C58–C57–C66	127.0(14)	C57–C58–C59	107.2(12)
C57–C58–C68	123.4(14)	C59–C58–C68	129.5(12)
N3–C59–C58	107.4(11)	N3–C59–C60	120.4(12)

C58–C59–C60	132.2(11)	C59–C60–C61	122.0(10)
C59–C60–C73	117.4(11)	C61–C60–C73	120.5(11)
N4–C61–C60	121.3(12)	N4–C61–C62	108.1(11)
C60–C61–C62	130.6(11)	C61–C62–C63	105.9(11)
C61–C62–C69	129.6(13)	C63–C62–C69	124.5(14)
C62–C63–C64	108.1(14)	C62–C63–C70	127.8(14)
C64–C63–C70	124.0(13)	N4–C64–C63	110.5(12)
N4–C64–C72	124.8(13)	C63–C64–C72	124.6(15)
C56–C65–H65A	109.5	C56–C65–H65B	109.5
C56–C65–H65C	109.5	H65A–C65–H65B	109.5
H65A–C65–H65C	109.5	H65B–C65–H65C	109.5
C57–C66–H66A	109.7	C57–C66–H66B	109.7
C57–C66–C67	110(2)	H66A–C66–H66B	108.2
H66A–C66–C67	109.7	H66B–C66–C67	109.7
C66–C67–H67A	109.5	C66–C67–H67B	109.5
C66–C67–H67C	109.5	H67A–C67–H67B	109.5
H67A–C67–H67C	109.5	H67B–C67–H67C	109.5
C58–C68–H68A	109.5	C58–C68–H68B	109.5
C58–C68–H68C	109.5	H68A–C68–H68B	109.5
H68A–C68–H68C	109.5	H68B–C68–H68C	109.5
C62–C69–H69A	109.5	C62–C69–H69B	109.5
C62–C69–H69C	109.5	H69A–C69–H69B	109.5
H69A–C69–H69C	109.5	H69B–C69–H69C	109.5
C63–C70–H70A	108.8	C63–C70–H70B	108.8
C63–C70–C71	113.8(16)	H70A–C70–H70B	107.7
H70A–C70–C71	108.8	H70B–C70–C71	108.8
C70–C71–H71A	109.5	C70–C71–H71B	109.5
C70–C71–H71C	109.5	H71A–C71–H71B	109.5
H71A–C71–H71C	109.5	H71B–C71–H71C	109.5
C64–C72–H72A	109.5	C64–C72–H72B	109.5
C64–C72–H72C	109.5	H72A–C72–H72B	109.5
H72A–C72–H72C	109.5	H72B–C72–H72C	109.5
C60–C73–C74	121.3(10)	C60–C73–C78	119.4(10)
C74–C73–C78	119.3(10)	C73–C74–H74	119.5

C73–C74–C75	121.0(10)	H74–C74–C75	119.5
C74–C75–H75	119.9	C74–C75–C76	120.2(10)
H75–C75–C76	119.9	C75–C76–C77	118.1(9)
C75–C76–P5	118.0(8)	C77–C76–P5	123.1(8)
C76–C77–H77	119.6	C76–C77–C78	120.9(10)
H77–C77–C78	119.6	C73–C78–C77	120.5(10)
C73–C78–H78	119.8	C77–C78–H78	119.8
B2–C79–H79A	109.5	B2–C79–H79B	109.5
B2–C79–H79C	109.5	H79A–C79–H79B	109.5
H79A–C79–H79C	109.5	H79B–C79–H79C	109.5
B2–C80–H80A	109.5	B2–C80–H80B	109.5
B2–C80–H80C	109.5	H80A–C80–H80B	109.5
H80A–C80–H80C	109.5	H80B–C80–H80C	109.5
Re2–P4–C81	120.4(3)	Re2–P4–C87	118.3(3)
Re2–P4–C93	106.5(3)	C81–P4–C87	102.3(4)
C81–P4–C93	100.2(4)	C87–P4–C93	107.1(4)
Re2–P5–C76	116.9(3)	Re2–P5–C94	112.3(3)
Re2–P5–C95	110.8(3)	C76–P5–C94	105.9(5)
C76–P5–C95	106.2(4)	C94–P5–C95	103.8(5)
Re2–P6–C96	106.0(3)	Re2–P6–C97	128.1(3)
Re2–P6–C103	111.2(3)	C96–P6–C97	105.4(4)
C96–P6–C103	105.0(4)	C97–P6–C103	99.1(4)
P4–C81–C82	119.6(7)	P4–C81–C86	121.0(7)
C82–C81–C86	119.4(8)	C81–C82–H82	119.8
C81–C82–C83	120.3(9)	H82–C82–C83	119.8
C82–C83–H83	120.2	C82–C83–C84	119.6(10)
H83–C83–C84	120.2	C83–C84–H84	119.7
C83–C84–C85	120.6(9)	H84–C84–C85	119.7
C84–C85–H85	120.1	C84–C85–C86	119.9(9)
H85–C85–C86	120.1	C81–C86–C85	120.1(9)
C81–C86–H86	119.9	C85–C86–H86	119.9
P4–C87–C88	124.1(8)	P4–C87–C92	115.5(7)
C88–C87–C92	120.3(9)	C87–C88–H88	120.3
C87–C88–C89	119.5(10)	H88–C88–C89	120.3

C88–C89–H89	119.6	C88–C89–C90	120.8(10)
H89–C89–C90	119.6	C89–C90–H90	119.9
C89–C90–C91	120.2(9)	H90–C90–C91	119.9
C90–C91–H91	119.9	C90–C91–C92	120.1(10)
H91–C91–C92	119.9	C87–C92–C91	119.0(9)
C87–C92–H92	120.5	C91–C92–H92	120.5
P4–C93–H93A	109.1	P4–C93–H93B	109.1
P4–C93–C94	112.5(6)	H93A–C93–H93B	107.8
H93A–C93–C94	109.1	H93B–C93–C94	109.1
P5–C94–C93	111.6(6)	P5–C94–H94A	109.3
P5–C94–H94B	109.3	C93–C94–H94A	109.3
C93–C94–H94B	109.3	H94A–C94–H94B	108.0
P5–C95–H95A	109.8	P5–C95–H95B	109.8
P5–C95–C96	109.6(6)	H95A–C95–H95B	108.2
H95A–C95–C96	109.8	H95B–C95–C96	109.8
P6–C96–C95	109.6(6)	P6–C96–H96A	109.7
P6–C96–H96B	109.7	C95–C96–H96A	109.7
C95–C96–H96B	109.7	H96A–C96–H96B	108.2
P6–C97–C98	121.6(7)	P6–C97–C102	120.5(7)
C98–C97–C102	117.9(8)	C97–C98–H98	119.3
C97–C98–C99	121.4(9)	H98–C98–C99	119.3
C98–C99–H99	120.6	C98–C99–C100	118.8(9)
H99–C99–C100	120.6	C99–C100–H100	120.1
C99–C100–C101	119.8(9)	H100–C100–C101	120.1
C100–C101–H101	119.5	C100–C101–C102	121.0(10)
H101–C101–C102	119.5	C97–C102–C101	121.1(9)
C97–C102–H102	119.4	C101–C102–H102	119.4
P6–C103–C104	117.0(7)	P6–C103–C108	124.6(8)
C104–C103–C108	118.4(9)	C103–C104–H104	119.8
C103–C104–C105	120.4(9)	H104–C104–C105	119.8
C104–C105–H105	119.7	C104–C105–C106	120.5(10)
H105–C105–C106	119.7	C105–C106–H106	120.4
C105–C106–C107	119.3(10)	H106–C106–C107	120.4
C106–C107–H107	119.9	C106–C107–C108	120.2(10)

H107–C107–C108	119.9	C103–C108–C107	121.1(10)
C103–C108–H108	119.4	C107–C108–H108	119.4
Re2–C109–O3	174.2(8)	Re2–C110–O4	168.1(8)
C111–O5–C114	112.9(14)	O5–C111–H11C	110.5
O5–C111–H11D	110.5	O5–C111–C112	106.3(13)
H11C–C111–H11D	108.7	H11C–C111–C112	110.5
H11D–C111–C112	110.5	C111–C112–H11E	110.5
C111–C112–H11F	110.5	C111–C112–C113	106.3(12)
H11E–C112–H11F	108.7	H11E–C112–C113	110.5
H11F–C112–C113	110.5	C112–C113–H11G	110.2
C112–C113–H11H	110.2	C112–C113–C114	107.3(12)
H11G–C113–H11H	108.5	H11G–C113–C114	110.2
H11H–C113–C114	110.2	O5–C114–C113	102.9(12)
O5–C114–H11I	111.2	O5–C114–H11J	111.2
C113–C114–H11I	111.2	C113–C114–H11J	111.2
H11I–C114–H11J	109.1	C115–O6–C118	109.2(16)
O6–C115–H11K	110.7	O6–C115–H11L	110.7
O6–C115–C116	105.0(15)	H11K–C115–H11L	108.8
H11K–C115–C116	110.7	H11L–C115–C116	110.7
C115–C116–H11M	110.2	C115–C116–H11N	110.2
C115–C116–C117	107.4(13)	H11M–C116–H11N	108.5
H11M–C116–C117	110.2	H11N–C116–C117	110.2
C116–C117–H11O	110.3	C116–C117–H11P	110.3
C116–C117–C118	107.1(14)	H11O–C117–H11P	108.6
H11O–C117–C118	110.3	H11P–C117–C118	110.3
O6–C118–C117	105.8(15)	O6–C118–H11Q	110.6
O6–C118–H11R	110.6	C117–C118–H11Q	110.6
C117–C118–H11R	110.6	H11Q–C118–H11R	108.7
C119–O7–C122	106(2)	O7–C119–H11S	111.0
O7–C119–H11T	111.0	O7–C119–C120	104(2)
H11S–C119–H11T	109.0	H11S–C119–C120	111.0
H11T–C119–C120	111.0	C119–C120–H12D	110.8
C119–C120–H12E	110.8	C119–C120–C121	104.6(18)
H12D–C120–H12E	108.9	H12D–C120–C121	110.8

H12E–C120–C121	110.8	C120–C121–H12F	110.3
C120–C121–H12G	110.3	C120–C121–C122	107.2(15)
H12F–C121–H12G	108.5	H12F–C121–C122	110.3
H12G–C121–C122	110.3	O7–C122–C121	105.6(19)
O7–C122–H12H	110.6	O7–C122–H12I	110.6
C121–C122–H12H	110.6	C121–C122–H12I	110.6
H12H–C122–H12I	108.8	C123–O8–C126	109(3)
O8–C123–H12J	110.8	O8–C123–H12K	110.8
O8–C123–C124	105(2)	H12J–C123–H12K	108.9
H12J–C123–C124	110.8	H12K–C123–C124	110.8
C123–C124–H12L	110.4	C123–C124–H12M	110.4
C123–C124–C125	106(2)	H12L–C124–H12M	108.6
H12L–C124–C125	110.4	H12M–C124–C125	110.4
C124–C125–H12N	110.5	C124–C125–H12O	110.5
C124–C125–C126	106.3(16)	H12N–C125–H12O	108.7
H12N–C125–C126	110.5	H12O–C125–C126	110.5
O8–C126–C125	106.9(19)	O8–C126–H12P	110.3
O8–C126–H12Q	110.3	C125–C126–H12P	110.3
C125–C126–H12Q	110.3	H12P–C126–H12Q	108.6

Table 3c:4. Torsion angles [°] for ljh102 (**3c**).

N2–B1–N1–C1	176.4(12)	N2–B1–N1–C4	−11.3(19)
C24–B1–N1–C1	58.8(19)	C24–B1–N1–C4	−128.9(13)
C25–B1–N1–C1	−66.5(17)	C25–B1–N1–C4	105.9(14)
N1–B1–N2–C6	6.8(18)	N1–B1–N2–C9	−175.9(12)
C24–B1–N2–C6	124.7(13)	C24–B1–N2–C9	−58.1(19)
C25–B1–N2–C6	−109.7(14)	C25–B1–N2–C9	67.5(18)
B1–N1–C1–C2	173.5(13)	B1–N1–C1–C10	−5(2)
C4–N1–C1–C2	0.0(15)	C4–N1–C1–C10	−178.2(13)
N1–C1–C2–C3	−0.1(16)	N1–C1–C2–C11	−179.1(13)
C10–C1–C2–C3	178.0(14)	C10–C1–C2–C11	−1(2)
C1–C2–C3–C4	0.2(15)	C1–C2–C3–C13	−179.6(12)
C11–C2–C3–C4	179.2(14)	C11–C2–C3–C13	−1(2)
B1–N1–C4–C3	−173.5(13)	B1–N1–C4–C5	9.0(19)
C1–N1–C4–C3	0.2(14)	C1–N1–C4–C5	−177.3(11)
C2–C3–C4–N1	−0.2(14)	C2–C3–C4–C5	176.9(13)
C13–C3–C4–N1	179.6(12)	C13–C3–C4–C5	−3(2)
N1–C4–C5–C6	−0.3(17)	N1–C4–C5–C18	−179.0(10)
C3–C4–C5–C6	−177.2(12)	C3–C4–C5–C18	4.1(19)
B1–N2–C6–C5	−0.2(19)	B1–N2–C6–C7	178.8(12)
C9–N2–C6–C5	−177.9(11)	C9–N2–C6–C7	1.0(14)
C4–C5–C6–N2	−3.9(18)	C4–C5–C6–C7	177.4(12)
C18–C5–C6–N2	174.7(10)	C18–C5–C6–C7	−4.0(19)
N2–C6–C7–C8	0.2(14)	N2–C6–C7–C14	177.2(12)
C5–C6–C7–C8	179.0(13)	C5–C6–C7–C14	−4(2)
C6–C7–C8–C9	−1.3(15)	C6–C7–C8–C15	−177.9(14)
C14–C7–C8–C9	−178.5(12)	C14–C7–C8–C15	5(2)
B1–N2–C9–C8	−179.5(13)	B1–N2–C9–C17	0(2)
C6–N2–C9–C8	−1.9(15)	C6–N2–C9–C17	177.6(13)
C7–C8–C9–N2	2.1(17)	C7–C8–C9–C17	−177.4(14)
C15–C8–C9–N2	178.7(13)	C15–C8–C9–C17	−1(2)

C1–C2–C11–C12	96.4(17)	C3–C2–C11–C12	−82.4(18)
C7–C8–C15–C16	91.1(19)	C9–C8–C15–C16	−85.0(17)
C4–C5–C18–C19	−87.3(14)	C4–C5–C18–C23	87.6(13)
C6–C5–C18–C19	94.0(14)	C6–C5–C18–C23	−91.1(14)
C5–C18–C19–C20	175.2(10)	C23–C18–C19–C20	0.2(16)
C18–C19–C20–C21	0.3(16)	C19–C20–C21–C22	0.7(15)
C19–C20–C21–P2	179.3(8)	C20–C21–C22–C23	−2.2(15)
P2–C21–C22–C23	179.1(8)	C21–C22–C23–C18	2.8(17)
C5–C18–C23–C22	−176.8(10)	C19–C18–C23–C22	−1.7(16)
C20–C21–P2–Re1	2.4(10)	C20–C21–P2–C39	131.9(8)
C20–C21–P2–C40	−121.7(8)	C22–C21–P2–Re1	−179.0(6)
C22–C21–P2–C39	−49.5(9)	C22–C21–P2–C40	56.9(9)
Re1–P1–C26–C27	−13.2(10)	Re1–P1–C26–C31	168.8(7)
C32–P1–C26–C27	122.0(9)	C32–P1–C26–C31	−56.0(9)
C38–P1–C26–C27	−130.0(9)	C38–P1–C26–C31	52.0(9)
P1–C26–C27–C28	−175.0(9)	C31–C26–C27–C28	3.1(17)
C26–C27–C28–C29	−2(2)	C27–C28–C29–C30	−1(2)
C28–C29–C30–C31	2.8(18)	C29–C30–C31–C26	−1.5(17)
P1–C26–C31–C30	176.5(8)	C27–C26–C31–C30	−1.6(15)
Re1–P1–C32–C33	81.5(8)	Re1–P1–C32–C37	−93.4(7)
C26–P1–C32–C33	−52.9(8)	C26–P1–C32–C37	132.3(8)
C38–P1–C32–C33	−159.8(7)	C38–P1–C32–C37	25.3(9)
P1–C32–C33–C34	−177.1(7)	C37–C32–C33–C34	−2.0(14)
C32–C33–C34–C35	1.7(15)	C33–C34–C35–C36	−0.5(15)
C34–C35–C36–C37	−0.3(16)	P1–C32–C37–C36	176.0(7)
C33–C32–C37–C36	1.2(13)	C35–C36–C37–C32	−0.1(14)
Re1–P1–C38–C39	41.5(7)	C26–P1–C38–C39	168.8(6)
C32–P1–C38–C39	−88.1(7)	P1–C38–C39–P2	−38.0(8)
Re1–P2–C39–C38	16.2(8)	C21–P2–C39–C38	−119.6(7)
C40–P2–C39–C38	135.8(7)	Re1–P2–C40–C41	42.6(7)
C21–P2–C40–C41	175.1(6)	C39–P2–C40–C41	−78.5(7)
P2–C40–C41–P3	−53.8(7)	Re1–P3–C41–C40	41.1(7)

C42–P3–C41–C40	−78.8(7)	C48–P3–C41–C40	174.5(6)
Re1–P3–C42–C43	55.1(8)	Re1–P3–C42–C47	−125.0(7)
C41–P3–C42–C43	170.1(7)	C41–P3–C42–C47	−10.0(9)
C48–P3–C42–C43	−81.1(8)	C48–P3–C42–C47	98.8(8)
P3–C42–C43–C44	179.9(7)	C47–C42–C43–C44	0.0(14)
C42–C43–C44–C45	0.8(15)	C43–C44–C45–C46	−0.9(15)
C44–C45–C46–C47	0.2(15)	C45–C46–C47–C42	0.6(15)
P3–C42–C47–C46	179.4(8)	C43–C42–C47–C46	−0.7(15)
Re1–P3–C48–C49	−48.1(8)	Re1–P3–C48–C53	135.1(6)
C41–P3–C48–C49	−169.8(7)	C41–P3–C48–C53	13.4(8)
C42–P3–C48–C49	81.1(8)	C42–P3–C48–C53	−95.6(8)
P3–C48–C49–C50	−177.6(7)	C53–C48–C49–C50	−0.7(13)
C48–C49–C50–C51	1.3(13)	C49–C50–C51–C52	−2.0(14)
C50–C51–C52–C53	2.2(14)	C51–C52–C53–C48	−1.6(14)
P3–C48–C53–C52	177.6(7)	C49–C48–C53–C52	0.9(13)
N4–B2–N3–C56	178.0(14)	N4–B2–N3–C59	3(2)
C79–B2–N3–C56	−64(2)	C79–B2–N3–C59	120.9(15)
C80–B2–N3–C56	61(2)	C80–B2–N3–C59	−114.7(14)
N3–B2–N4–C61	2(2)	N3–B2–N4–C64	−179.3(15)
C79–B2–N4–C61	−116.6(15)	C79–B2–N4–C64	62(2)
C80–B2–N4–C61	118.2(15)	C80–B2–N4–C64	−63(2)
B2–N3–C56–C57	−176.6(15)	B2–N3–C56–C65	5(3)
C59–N3–C56–C57	−0.6(18)	C59–N3–C56–C65	−179.5(16)
N3–C56–C57–C58	1(2)	N3–C56–C57–C66	−176.0(17)
C65–C56–C57–C58	−179.6(16)	C65–C56–C57–C66	3(3)
C56–C57–C58–C59	−1.7(18)	C56–C57–C58–C68	177.6(14)
C66–C57–C58–C59	175.8(17)	C66–C57–C58–C68	−5(3)
B2–N3–C59–C58	175.7(14)	B2–N3–C59–C60	−5(2)
C56–N3–C59–C58	−0.4(16)	C56–N3–C59–C60	179.1(13)
C57–C58–C59–N3	1.3(16)	C57–C58–C59–C60	−178.2(15)
C68–C58–C59–N3	−177.9(14)	C68–C58–C59–C60	3(3)
N3–C59–C60–C61	2(2)	N3–C59–C60–C73	−174.0(11)

C58–C59–C60–C61	−179.0(14)	C58–C59–C60–C73	5(2)
C59–C60–C61–N4	3(2)	C59–C60–C61–C62	−177.3(14)
C73–C60–C61–N4	178.9(11)	C73–C60–C61–C62	−2(2)
B2–N4–C61–C60	−5(2)	B2–N4–C61–C62	175.1(14)
C64–N4–C61–C60	175.9(14)	C64–N4–C61–C62	−3.5(16)
N4–C61–C62–C63	3.2(16)	N4–C61–C62–C69	−175.5(14)
C60–C61–C62–C63	−176.2(15)	C60–C61–C62–C69	5(3)
C61–C62–C63–C64	−1.7(18)	C61–C62–C63–C70	−179.8(17)
C69–C62–C63–C64	177.1(14)	C69–C62–C63–C70	−1(3)
B2–N4–C64–C63	−176.1(15)	B2–N4–C64–C72	0(3)
C61–N4–C64–C63	2.5(18)	C61–N4–C64–C72	178.6(15)
C62–C63–C64–N4	−1(2)	C62–C63–C64–C72	−176.6(16)
C70–C63–C64–N4	177.7(16)	C70–C63–C64–C72	2(3)
C56–C57–C66–C67	−90(2)	C58–C57–C66–C67	93(2)
C62–C63–C70–C71	−89(2)	C64–C63–C70–C71	93(2)
C59–C60–C73–C74	73.2(16)	C59–C60–C73–C78	−108.3(14)
C61–C60–C73–C74	−102.5(16)	C61–C60–C73–C78	76.0(16)
C60–C73–C74–C75	176.1(11)	C78–C73–C74–C75	−2.5(18)
C73–C74–C75–C76	−1.1(17)	C74–C75–C76–C77	3.0(16)
C74–C75–C76–P5	−166.8(9)	C75–C76–C77–C78	−1.2(17)
P5–C76–C77–C78	168.1(9)	C76–C77–C78–C73	−2.5(18)
C60–C73–C78–C77	−174.3(11)	C74–C73–C78–C77	4.3(18)
C75–C76–P5–Re2	64.9(9)	C75–C76–P5–C94	−61.0(9)
C75–C76–P5–C95	−170.9(8)	C77–C76–P5–Re2	−104.3(9)
C77–C76–P5–C94	129.8(9)	C77–C76–P5–C95	19.8(11)
Re2–P4–C81–C82	−178.0(7)	Re2–P4–C81–C86	1.3(9)
C87–P4–C81–C82	48.3(9)	C87–P4–C81–C86	−132.4(8)
C93–P4–C81–C82	−61.9(9)	C93–P4–C81–C86	117.4(8)
P4–C81–C82–C83	178.7(8)	C86–C81–C82–C83	−0.6(15)
C81–C82–C83–C84	−0.4(16)	C82–C83–C84–C85	1.1(16)
C83–C84–C85–C86	−0.7(15)	C84–C85–C86–C81	−0.3(14)
P4–C81–C86–C85	−178.3(7)	C82–C81–C86–C85	1.0(14)

Re2–P4–C87–C88	108.3(7)	Re2–P4–C87–C92	−68.9(7)
C81–P4–C87–C88	−116.7(8)	C81–P4–C87–C92	66.1(7)
C93–P4–C87–C88	−11.8(9)	C93–P4–C87–C92	170.9(6)
P4–C87–C88–C89	−177.1(7)	C92–C87–C88–C89	0.0(13)
C87–C88–C89–C90	−0.9(14)	C88–C89–C90–C91	0.4(14)
C89–C90–C91–C92	0.9(14)	C90–C91–C92–C87	−1.8(13)
P4–C87–C92–C91	178.7(7)	C88–C87–C92–C91	1.3(13)
Re2–P4–C93–C94	−41.1(7)	C81–P4–C93–C94	−167.3(6)
C87–P4–C93–C94	86.3(7)	P4–C93–C94–P5	38.7(8)
Re2–P5–C94–C93	−18.6(7)	C76–P5–C94–C93	110.1(7)
C95–P5–C94–C93	−138.3(7)	Re2–P5–C95–C96	−42.6(7)
C76–P5–C95–C96	−170.5(7)	C94–P5–C95–C96	78.1(7)
P5–C95–C96–P6	52.3(8)	Re2–P6–C96–C95	−38.8(7)
C97–P6–C96–C95	−176.9(6)	C103–P6–C96–C95	79.0(7)
Re2–P6–C97–C98	−135.0(7)	Re2–P6–C97–C102	48.8(10)
C96–P6–C97–C98	−9.7(9)	C96–P6–C97–C102	174.1(8)
C103–P6–C97–C98	98.7(9)	C103–P6–C97–C102	−77.5(8)
P6–C97–C98–C99	−175.7(7)	C102–C97–C98–C99	0.6(14)
C97–C98–C99–C100	−0.9(14)	C98–C99–C100–C101	1.2(15)
C99–C100–C101–C102	−1.2(15)	C100–C101–C102–C97	0.9(15)
P6–C97–C102–C101	175.8(8)	C98–C97–C102–C101	−0.6(14)
Re2–P6–C103–C104	−59.2(8)	Re2–P6–C103–C108	118.5(8)
C96–P6–C103–C104	−173.4(7)	C96–P6–C103–C108	4.4(9)
C97–P6–C103–C104	77.9(8)	C97–P6–C103–C108	−104.3(9)
P6–C103–C104–C105	178.8(8)	C108–C103–C104–C105	0.9(14)
C103–C104–C105–C106	−1.4(16)	C104–C105–C106–C107	−0.4(16)
C105–C106–C107–C108	2.6(16)	P6–C103–C108–C107	−176.4(8)
C104–C103–C108–C107	1.4(14)	C106–C107–C108–C103	−3.1(16)
C114–O5–C111–C112	−7(3)	O5–C111–C112–C113	−7(3)
C111–C112–C113–C114	17(3)	C111–O5–C114–C113	17(2)
C112–C113–C114–O5	−21(2)	C118–O6–C115–C116	−23(3)
O6–C115–C116–C117	14(4)	C115–C116–C117–C118	0(5)

C115–O6–C118–C117	23(3)	C116–C117–C118–O6	−14(4)
C122–O7–C119–C120	−36(6)	O7–C119–C120–C121	32(6)
C119–C120–C121–C122	−15(8)	C119–O7–C122–C121	27(7)
C120–C121–C122–O7	−7(8)	C126–O8–C123–C124	−25(9)
O8–C123–C124–C125	25(9)	C123–C124–C125–C126	−15(11)
C123–O8–C126–C125	16(12)	C124–C125–C126–O8	0(13)

Table 3c:5. Anisotropic displacement parameters (Å^2) for ljh102 (**3c**). The anisotropic displacement factor exponent takes the form: $-2\pi^2[h^2a^{*2}U^{11} + ... + 2hka^*b^*U^{12}]$

	U^{11}	U^{22}	U^{33}	U^{23}	U^{13}	U^{12}
Re1	0.0237(2)	0.0201(2)	0.0257(2)	−0.00162(15)	−0.00410(16)	−0.00263(15)
B1	0.048(5)	0.110(8)	0.034(5)	0.017(5)	−0.013(4)	−0.014(5)
N1	0.048(4)	0.069(6)	0.029(4)	0.008(3)	−0.008(3)	−0.008(3)
N2	0.042(4)	0.069(6)	0.037(4)	0.008(3)	−0.012(3)	−0.007(3)
C1	0.051(5)	0.078(8)	0.040(4)	0.011(4)	−0.004(3)	−0.012(4)
C2	0.048(4)	0.076(7)	0.043(4)	0.008(4)	−0.002(3)	−0.013(4)
C3	0.040(4)	0.054(6)	0.039(4)	0.001(4)	−0.007(3)	−0.008(4)
C4	0.041(4)	0.057(6)	0.033(4)	0.003(4)	−0.008(3)	−0.006(4)
C5	0.045(4)	0.049(6)	0.031(4)	0.000(3)	−0.010(3)	−0.004(3)
C6	0.042(4)	0.053(6)	0.035(4)	0.001(4)	−0.011(3)	−0.004(3)
C7	0.047(5)	0.065(7)	0.037(4)	0.003(4)	−0.004(3)	−0.001(4)
C8	0.045(4)	0.085(8)	0.047(5)	0.010(4)	−0.007(3)	0.001(4)
C9	0.044(4)	0.071(7)	0.045(4)	0.007(4)	−0.012(3)	−0.007(4)
C10	0.073(9)	0.115(12)	0.044(5)	0.020(6)	−0.005(5)	−0.013(8)
C11	0.045(5)	0.110(9)	0.045(6)	0.010(6)	−0.007(4)	−0.017(5)
C12	0.045(7)	0.120(9)	0.063(8)	−0.003(7)	0.002(6)	−0.017(6)
C13	0.042(6)	0.085(9)	0.041(6)	0.000(5)	−0.010(4)	−0.004(5)
C14	0.050(7)	0.072(8)	0.036(5)	−0.001(4)	−0.003(4)	0.004(6)
C15	0.045(6)	0.116(9)	0.054(7)	0.012(6)	−0.005(5)	0.003(5)
C16	0.058(8)	0.130(10)	0.053(8)	0.006(7)	0.007(7)	−0.018(7)
C17	0.040(6)	0.121(12)	0.052(6)	0.019(7)	−0.014(5)	−0.009(6)
C18	0.039(5)	0.049(4)	0.030(4)	0.000(3)	−0.005(3)	−0.007(4)
C19	0.050(6)	0.042(5)	0.035(4)	0.003(3)	−0.011(4)	−0.003(4)
C20	0.040(5)	0.035(4)	0.030(4)	−0.002(3)	−0.015(4)	−0.009(3)
C21	0.029(4)	0.034(4)	0.026(4)	−0.001(3)	−0.006(3)	−0.006(3)
C22	0.047(5)	0.037(4)	0.029(4)	0.001(3)	−0.008(4)	−0.010(4)
C23	0.051(6)	0.043(4)	0.034(5)	−0.003(3)	−0.013(4)	−0.006(4)

C24	0.057(8)	0.133(9)	0.077(9)	0.043(7)	−0.009(7)	−0.003(7)
C25	0.076(9)	0.145(10)	0.051(7)	−0.010(7)	0.004(6)	−0.036(7)
P1	0.0262(12)	0.0242(11)	0.0306(12)	−0.0030(9)	−0.0022(9)	−0.0015(9)
P2	0.0286(12)	0.0271(12)	0.0249(12)	−0.0025(9)	−0.0054(9)	−0.0018(9)
P3	0.0242(11)	0.0207(11)	0.0239(11)	−0.0021(8)	−0.0036(9)	0.0002(8)
C26	0.028(4)	0.029(4)	0.030(5)	0.002(3)	−0.004(3)	−0.002(3)
C27	0.032(4)	0.040(4)	0.070(7)	−0.012(4)	−0.004(4)	−0.006(3)
C28	0.034(5)	0.063(6)	0.082(8)	−0.019(5)	−0.003(5)	−0.021(4)
C29	0.036(5)	0.072(6)	0.054(7)	0.001(5)	−0.004(4)	−0.012(4)
C30	0.027(4)	0.067(5)	0.045(6)	0.005(4)	−0.002(4)	−0.001(4)
C31	0.030(4)	0.044(5)	0.041(5)	0.004(4)	−0.003(4)	0.004(3)
C32	0.022(4)	0.027(3)	0.032(4)	−0.003(3)	0.004(3)	0.002(3)
C33	0.032(5)	0.031(4)	0.037(4)	−0.006(3)	−0.004(3)	−0.001(3)
C34	0.034(5)	0.042(4)	0.037(5)	−0.001(3)	0.000(4)	0.004(4)
C35	0.042(5)	0.036(4)	0.049(5)	0.003(4)	0.001(4)	0.005(4)
C36	0.039(5)	0.033(4)	0.050(5)	0.003(3)	0.006(4)	0.005(4)
C37	0.031(5)	0.026(4)	0.042(5)	−0.005(3)	0.001(4)	0.001(3)
C38	0.036(4)	0.029(4)	0.030(4)	−0.009(3)	−0.007(3)	0.000(3)
C39	0.034(4)	0.031(4)	0.029(5)	−0.013(4)	−0.004(3)	−0.003(3)
C40	0.028(4)	0.032(4)	0.027(4)	−0.006(3)	−0.009(3)	−0.011(3)
C41	0.029(4)	0.029(4)	0.026(4)	−0.006(3)	−0.004(3)	0.003(3)
C42	0.028(4)	0.026(4)	0.024(4)	0.004(3)	−0.005(3)	0.003(3)
C43	0.043(5)	0.029(4)	0.033(5)	0.001(3)	0.002(4)	0.003(3)
C44	0.043(4)	0.040(5)	0.036(5)	0.004(4)	0.001(4)	0.011(3)
C45	0.040(5)	0.052(5)	0.027(5)	0.010(4)	0.003(4)	0.008(4)
C46	0.034(4)	0.051(5)	0.044(6)	0.010(4)	0.005(4)	0.001(4)
C47	0.032(4)	0.044(5)	0.042(5)	−0.003(4)	−0.001(4)	−0.002(3)
C48	0.021(4)	0.030(4)	0.026(4)	−0.003(3)	0.000(3)	−0.002(3)
C49	0.026(4)	0.030(4)	0.032(4)	−0.001(3)	0.000(3)	−0.006(3)
C50	0.032(5)	0.035(4)	0.028(4)	−0.004(3)	0.003(3)	−0.002(3)
C51	0.026(4)	0.037(4)	0.031(4)	0.001(3)	−0.006(3)	−0.002(3)
C52	0.026(4)	0.032(4)	0.034(4)	0.001(3)	−0.004(3)	0.000(3)

C53	0.031(5)	0.030(4)	0.029(4)	0.002(3)	−0.002(3)	−0.003(3)
C54	0.021(4)	0.027(4)	0.033(4)	0.001(3)	−0.012(3)	0.006(3)
O1	0.060(5)	0.048(4)	0.047(4)	0.009(3)	0.002(4)	−0.014(4)
C55	0.021(3)	0.024(4)	0.039(5)	−0.005(3)	−0.008(3)	−0.015(3)
O2	0.032(3)	0.036(4)	0.053(4)	−0.001(3)	−0.005(3)	0.004(3)
Cl1	0.0320(12)	0.0256(11)	0.0341(12)	−0.0027(9)	−0.0083(9)	−0.0038(9)
Re2	0.0239(2)	0.0197(2)	0.0321(2)	−0.00183(16)	−0.00311(17)	−0.00326(15)
B2	0.082(7)	0.090(7)	0.048(5)	0.015(5)	0.003(5)	−0.036(6)
N3	0.078(6)	0.072(6)	0.044(4)	0.014(4)	0.003(4)	−0.022(5)
N4	0.070(5)	0.064(5)	0.052(4)	0.012(4)	0.004(4)	−0.024(4)
C56	0.091(8)	0.085(8)	0.046(5)	0.012(4)	−0.001(4)	−0.024(6)
C57	0.094(8)	0.082(7)	0.050(5)	0.004(4)	0.003(4)	−0.024(6)
C58	0.073(6)	0.056(6)	0.052(5)	0.001(4)	0.005(4)	−0.006(5)
C59	0.070(6)	0.055(6)	0.042(4)	0.005(3)	0.009(4)	−0.009(5)
C60	0.065(5)	0.053(5)	0.038(4)	0.005(4)	0.008(4)	−0.011(4)
C61	0.069(6)	0.052(6)	0.047(4)	0.004(4)	0.008(4)	−0.016(5)
C62	0.080(7)	0.061(7)	0.047(5)	0.001(4)	0.002(4)	−0.014(5)
C63	0.095(7)	0.067(7)	0.063(5)	0.005(5)	−0.003(5)	−0.029(6)
C64	0.086(7)	0.077(8)	0.062(5)	0.009(5)	−0.002(5)	−0.031(6)
C65	0.117(13)	0.130(13)	0.050(6)	0.022(7)	−0.005(7)	−0.048(11)
C66	0.135(11)	0.115(11)	0.058(7)	−0.002(7)	−0.010(7)	−0.048(9)
C67	0.23(3)	0.177(16)	0.144(18)	−0.064(15)	−0.034(18)	0.010(17)
C68	0.088(9)	0.070(8)	0.060(8)	0.001(6)	0.003(7)	−0.022(7)
C69	0.103(10)	0.055(7)	0.050(6)	0.000(5)	0.004(6)	−0.017(7)
C70	0.109(9)	0.107(10)	0.074(8)	−0.006(7)	−0.006(7)	−0.049(8)
C71	0.115(11)	0.147(15)	0.071(10)	−0.009(10)	−0.007(8)	−0.027(9)
C72	0.089(9)	0.099(10)	0.073(9)	0.017(8)	−0.007(7)	−0.043(8)
C73	0.058(5)	0.041(5)	0.038(5)	−0.001(4)	0.007(4)	−0.006(4)
C74	0.058(5)	0.041(5)	0.043(5)	0.002(4)	0.004(4)	−0.003(4)
C75	0.040(5)	0.038(5)	0.039(5)	0.001(4)	0.000(4)	−0.005(4)
C76	0.037(4)	0.032(4)	0.035(4)	−0.002(3)	−0.003(3)	−0.009(3)
C77	0.043(5)	0.046(5)	0.042(5)	0.005(4)	0.003(4)	−0.002(4)

C78	0.044(5)	0.047(5)	0.047(5)	0.003(4)	0.010(4)	−0.003(4)
C79	0.119(12)	0.092(7)	0.087(10)	0.027(6)	−0.018(9)	−0.035(7)
C80	0.097(9)	0.134(12)	0.057(8)	0.017(7)	0.009(7)	−0.019(8)
P4	0.0239(11)	0.0191(11)	0.0319(12)	−0.0017(9)	−0.0032(9)	−0.0033(8)
P5	0.0294(12)	0.0268(12)	0.0309(12)	−0.0030(9)	−0.0020(10)	−0.0022(9)
P6	0.0264(12)	0.0246(11)	0.0289(12)	−0.0025(9)	−0.0012(9)	−0.0009(9)
C81	0.029(4)	0.025(3)	0.030(5)	0.001(3)	−0.002(3)	0.004(3)
C82	0.029(4)	0.030(4)	0.054(6)	−0.002(4)	−0.006(4)	0.001(3)
C83	0.034(4)	0.036(4)	0.061(7)	−0.002(4)	−0.004(4)	0.007(3)
C84	0.034(4)	0.035(4)	0.045(6)	0.001(4)	−0.004(4)	0.005(3)
C85	0.038(4)	0.030(4)	0.035(5)	−0.001(4)	−0.003(4)	0.002(3)
C86	0.036(4)	0.026(4)	0.029(5)	0.000(3)	−0.001(4)	0.001(3)
C87	0.014(4)	0.025(3)	0.038(4)	0.001(3)	−0.008(3)	−0.005(3)
C88	0.025(5)	0.025(4)	0.051(5)	−0.001(3)	−0.008(4)	−0.004(3)
C89	0.028(5)	0.026(4)	0.053(4)	0.006(3)	−0.009(4)	−0.002(3)
C90	0.023(5)	0.036(4)	0.050(5)	0.009(3)	−0.004(4)	0.006(3)
C91	0.028(5)	0.041(4)	0.044(5)	0.005(3)	0.001(4)	0.004(4)
C92	0.023(4)	0.034(4)	0.036(4)	0.002(3)	−0.008(3)	0.001(3)
C93	0.024(4)	0.032(4)	0.036(4)	−0.001(3)	−0.008(3)	−0.005(3)
C94	0.032(4)	0.030(4)	0.029(4)	−0.009(3)	−0.006(3)	−0.011(3)
C95	0.039(5)	0.030(4)	0.031(4)	−0.002(3)	0.001(3)	0.000(4)
C96	0.022(4)	0.023(4)	0.031(4)	−0.004(3)	−0.007(3)	−0.001(3)
C97	0.032(5)	0.030(4)	0.031(4)	0.000(3)	−0.003(3)	0.001(3)
C98	0.029(5)	0.031(4)	0.033(4)	−0.004(3)	−0.008(3)	−0.005(3)
C99	0.031(5)	0.039(5)	0.036(4)	0.003(3)	−0.002(4)	−0.005(4)
C100	0.030(5)	0.048(4)	0.037(5)	−0.001(3)	0.000(4)	−0.005(4)
C101	0.032(5)	0.047(4)	0.036(4)	−0.005(3)	−0.002(4)	0.000(4)
C102	0.027(5)	0.034(4)	0.036(4)	−0.007(3)	−0.008(3)	0.000(3)
C103	0.029(4)	0.030(4)	0.035(5)	0.004(3)	−0.004(3)	−0.002(3)
C104	0.030(4)	0.034(4)	0.047(5)	−0.005(4)	−0.003(4)	−0.003(3)
C105	0.032(4)	0.034(5)	0.063(6)	−0.001(4)	−0.010(4)	−0.007(3)
C106	0.032(5)	0.035(5)	0.067(6)	0.005(4)	−0.014(4)	−0.003(3)

C107	0.030(4)	0.038(5)	0.066(6)	0.003(4)	−0.004(4)	0.004(3)
C108	0.030(4)	0.031(4)	0.045(5)	−0.001(4)	−0.004(4)	−0.003(3)
C109	0.026(4)	0.017(4)	0.043(4)	−0.002(3)	−0.001(3)	−0.006(3)
O3	0.065(5)	0.023(3)	0.045(4)	0.004(3)	−0.001(3)	−0.006(3)
C110	0.021(3)	0.037(5)	0.016(4)	−0.007(3)	−0.004(3)	−0.015(3)
O4	0.024(3)	0.059(5)	0.057(5)	0.006(4)	−0.001(3)	−0.015(3)
Cl2	0.0389(13)	0.0221(11)	0.0396(13)	−0.0084(9)	−0.0073(10)	−0.0019(9)
O5	0.099(7)	0.104(8)	0.113(8)	−0.012(6)	−0.030(6)	−0.031(6)
C111	0.102(9)	0.142(13)	0.153(13)	−0.052(11)	−0.040(8)	−0.009(8)
C112	0.101(9)	0.122(12)	0.160(14)	−0.045(11)	−0.050(9)	0.007(8)
C113	0.108(10)	0.113(11)	0.147(13)	−0.044(10)	−0.053(9)	0.011(8)
C114	0.103(9)	0.115(11)	0.106(11)	−0.009(8)	−0.038(7)	−0.006(7)
O6	0.180(15)	0.140(12)	0.115(10)	0.000(8)	−0.004(9)	0.034(10)
C115	0.197(16)	0.174(17)	0.127(12)	0.003(12)	−0.004(11)	−0.001(13)
C116	0.22(2)	0.23(2)	0.117(12)	0.030(12)	−0.008(11)	−0.046(16)
C117	0.24(2)	0.29(2)	0.115(12)	0.033(12)	−0.018(12)	−0.080(17)
C118	0.222(19)	0.222(18)	0.111(12)	0.013(11)	−0.001(11)	−0.040(15)
O7	0.39(4)	0.40(3)	0.36(3)	0.04(3)	0.00(3)	0.02(3)
C119	0.39(4)	0.41(4)	0.37(3)	0.04(3)	0.00(3)	0.02(3)
C120	0.38(4)	0.40(4)	0.38(4)	0.04(3)	−0.01(3)	0.03(3)
C121	0.40(4)	0.42(4)	0.37(3)	0.05(3)	−0.01(3)	0.01(3)
C122	0.40(4)	0.42(4)	0.36(3)	0.04(3)	−0.01(3)	0.01(3)
O8	0.77(5)	0.37(3)	0.70(5)	−0.12(4)	0.04(4)	−0.06(4)
C123	0.78(5)	0.38(3)	0.70(5)	−0.11(4)	0.04(4)	−0.05(4)
C124	0.78(5)	0.36(3)	0.69(5)	−0.11(4)	0.04(4)	−0.07(4)
C125	0.77(5)	0.31(3)	0.69(5)	−0.10(4)	0.04(4)	−0.08(4)
C126	0.77(5)	0.33(3)	0.69(5)	−0.11(4)	0.04(4)	−0.07(4)

Table 3c:6. Hydrogen coordinates and isotropic displacement parameters (Å^2) for ljh102 (**3c**).

	x	y	z	U
H10A	0.3567	0.8328	1.0057	0.119
H10B	0.4716	0.7884	0.9950	0.119
H10C	0.4696	0.8858	1.0042	0.119
H11A	0.2040	0.8301	0.9730	0.081
H11B	0.1452	0.8469	0.9296	0.081
H12A	0.0738	0.9411	0.9746	0.115
H12B	0.1955	0.9774	0.9806	0.115
H12C	0.1366	0.9942	0.9371	0.115
H13A	0.2727	0.9484	0.8296	0.085
H13B	0.1921	0.8812	0.8546	0.085
H13C	0.1937	0.9778	0.8651	0.085
H14A	0.6128	0.9619	0.7658	0.079
H14B	0.7302	1.0088	0.7613	0.079
H14C	0.7226	0.9091	0.7570	0.079
H15A	0.9771	0.8930	0.8287	0.088
H15B	0.9198	0.9198	0.7856	0.088
H16A	1.0464	1.0234	0.8000	0.122
H16B	0.9240	1.0630	0.7987	0.122
H16C	0.9813	1.0362	0.8418	0.122
H17A	0.9531	0.8976	0.8956	0.109
H17B	0.8723	0.9358	0.9283	0.109
H17C	0.8713	0.8361	0.9235	0.109
H19	0.4354	0.8402	0.7895	0.051
H20	0.3342	0.8828	0.7307	0.042
H22	0.3630	1.1269	0.7550	0.045
H23	0.4555	1.0841	0.8145	0.051
H24A	0.7425	0.7977	0.9561	0.139
H24B	0.6216	0.7833	0.9762	0.139
H24C	0.6485	0.7526	0.9321	0.139

H25A	0.7273	0.9656	0.9613	0.136
H25B	0.6257	1.0173	0.9399	0.136
H25C	0.6054	0.9559	0.9812	0.136
H27	−0.0671	0.8723	0.6436	0.056
H28	−0.2461	0.8141	0.6436	0.071
H29	−0.3998	0.8927	0.6593	0.066
H30	−0.3772	1.0303	0.6779	0.057
H31	−0.2031	1.0912	0.6751	0.047
H33C	−0.0831	1.0541	0.5761	0.040
H34	−0.1236	1.1625	0.5250	0.045
H35	−0.0739	1.3011	0.5325	0.051
H36	0.0132	1.3338	0.5916	0.050
H37	0.0515	1.2264	0.6445	0.039
H38A	−0.0177	1.1351	0.7033	0.037
H38B	0.0133	1.0435	0.7256	0.037
H39A	0.1633	1.1214	0.7393	0.037
H39B	0.1626	1.1750	0.6952	0.037
H40A	0.3721	1.1662	0.6796	0.035
H40B	0.4308	1.0876	0.6604	0.035
H41A	0.3677	1.1832	0.6049	0.033
H41B	0.2453	1.1840	0.6247	0.033
H43	0.3423	0.9111	0.5580	0.043
H44	0.5054	0.8586	0.5316	0.049
H45	0.6697	0.9327	0.5334	0.049
H46	0.6722	1.0648	0.5606	0.053
H47	0.5066	1.1178	0.5859	0.047
H49	0.1503	0.9894	0.5322	0.036
H50	0.0676	1.0455	0.4706	0.038
H51	0.0824	1.1900	0.4485	0.037
H52	0.1690	1.2803	0.4901	0.037
H53	0.2513	1.2255	0.5507	0.036
H65A	0.0651	0.3035	1.0119	0.152
H65B	−0.0593	0.2901	0.9975	0.152

H65C	0.0328	0.2188	0.9920	0.152
H66A	0.2772	0.4331	0.9612	0.123
H66B	0.2190	0.3688	0.9959	0.123
H67A	0.2004	0.5098	1.0130	0.271
H67B	0.1385	0.5361	0.9707	0.271
H67C	0.0804	0.4720	1.0054	0.271
H68A	0.1951	0.4842	0.8511	0.110
H68B	0.1910	0.5313	0.8920	0.110
H68C	0.2849	0.4616	0.8860	0.110
H69A	0.0013	0.3558	0.7523	0.105
H69B	−0.0377	0.2695	0.7354	0.105
H69C	−0.1213	0.3485	0.7350	0.105
H70A	−0.2171	0.1854	0.7439	0.116
H70B	−0.2872	0.1362	0.7806	0.116
H71A	−0.4062	0.2190	0.7366	0.167
H71B	−0.4091	0.2537	0.7808	0.167
H71C	−0.3390	0.3029	0.7440	0.167
H72A	−0.3306	0.1283	0.8434	0.133
H72B	−0.2424	0.1013	0.8784	0.133
H72C	−0.3271	0.1778	0.8836	0.133
H74	0.2248	0.3350	0.8134	0.057
H75	0.3221	0.4117	0.7600	0.047
H77	0.0571	0.5704	0.7412	0.053
H78	−0.0338	0.4971	0.7967	0.056
H79A	−0.0496	0.1435	0.9533	0.152
H79B	−0.1255	0.1104	0.9186	0.152
H79C	0.0021	0.1328	0.9089	0.152
H80A	−0.1922	0.2748	0.9631	0.147
H80B	−0.2215	0.3389	0.9238	0.147
H80C	−0.2724	0.2451	0.9289	0.147
H82	0.7069	0.4779	0.6450	0.045
H83	0.8416	0.3710	0.6434	0.053
H84	0.7875	0.2312	0.6405	0.046

H85	0.5999	0.1970	0.6370	0.042
H86	0.4654	0.3033	0.6379	0.037
H88	0.5542	0.6606	0.6431	0.040
H89	0.6112	0.7623	0.5900	0.044
H90	0.6156	0.7304	0.5234	0.045
H91	0.5614	0.5959	0.5074	0.046
H92	0.4973	0.4944	0.5594	0.037
H93A	0.5051	0.4683	0.7150	0.037
H93B	0.5687	0.5501	0.6938	0.037
H94A	0.4185	0.6354	0.6977	0.036
H94B	0.4106	0.5758	0.7402	0.036
H95A	0.1108	0.6298	0.6800	0.040
H95B	0.2084	0.6829	0.6981	0.040
H96A	0.3073	0.6895	0.6362	0.030
H96B	0.1831	0.7206	0.6257	0.030
H98	0.2881	0.7515	0.5657	0.037
H99	0.3446	0.8083	0.5004	0.043
H100	0.3632	0.7175	0.4478	0.047
H101	0.3185	0.5745	0.4610	0.046
H102	0.2631	0.5189	0.5253	0.039
H104	0.0822	0.4769	0.5602	0.044
H105	−0.1040	0.4669	0.5437	0.052
H106	−0.2333	0.5645	0.5650	0.054
H107	−0.1745	0.6713	0.6042	0.054
H108	0.0136	0.6869	0.6171	0.043
H11C	0.8596	0.6512	0.6838	0.155
H11D	0.8230	0.5743	0.7170	0.155
H11E	0.8813	0.6437	0.7678	0.150
H11F	0.9321	0.7145	0.7342	0.150
H11G	0.7985	0.7999	0.7414	0.144
H11H	0.7733	0.7421	0.7836	0.144
H11I	0.6253	0.6789	0.7573	0.129
H11J	0.6257	0.7614	0.7241	0.129

H11K	0.2488	1.1153	0.8717	0.201
H11L	0.1986	1.2070	0.8794	0.201
H11M	0.2742	1.1866	0.9413	0.231
H11N	0.3487	1.1087	0.9285	0.231
H11O	0.4867	1.1883	0.9215	0.261
H11P	0.4121	1.2663	0.9341	0.261
H11Q	0.3971	1.3208	0.8692	0.225
H11R	0.4959	1.2571	0.8591	0.225
H11S	−0.3278	0.5290	0.7705	0.473
H11T	−0.2774	0.4349	0.7813	0.473
H12D	−0.1705	0.5071	0.8242	0.472
H12E	−0.2675	0.5749	0.8310	0.472
H12F	−0.3041	0.4984	0.8860	0.486
H12G	−0.2310	0.4205	0.8723	0.486
H12H	−0.3827	0.3564	0.8517	0.480
H12I	−0.4565	0.4283	0.8708	0.480
H12J	0.4195	0.6905	0.9112	0.737
H12K	0.3926	0.5970	0.8985	0.737
H12L	0.2313	0.6600	0.8755	0.727
H12M	0.2857	0.7531	0.8698	0.727
H12N	0.2948	0.7374	0.8065	0.705
H12O	0.2724	0.6369	0.8147	0.705
H12P	0.4536	0.6116	0.8074	0.713
H12Q	0.4760	0.7120	0.7992	0.713

5.4 Crystallographic data for *fac*-[Re(CO)$_3$(triphos-Ph)][OTf] (4)

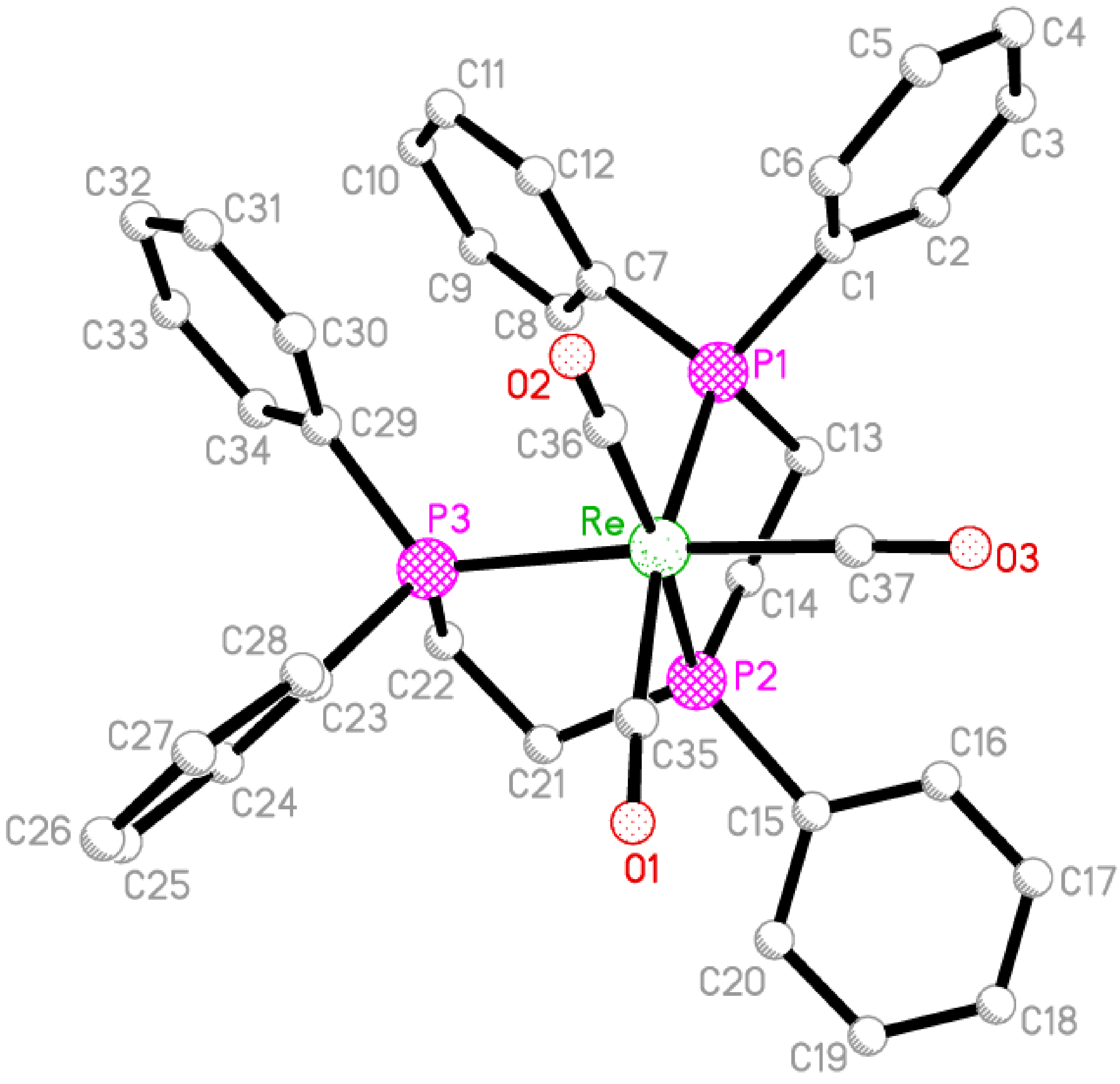

Table 4:1. Crystal data and structure refinement for ljh120152 (**4**).

Identification code	ljh120152 (**4**)	
Chemical formula (moiety)	$C_{37}H_{33}O_3P_3Re^{+}\cdot CF_3O_3S^{-}$	
Chemical formula (total)	$C_{38}H_{33}F_3O_6P_3ReS$	
Formula weight	953.81	
Temperature	150(2) K	
Radiation, wavelength	MoKα, 0.71073 Å	
Crystal system, space group	triclinic, $P\overline{1}$	
Unit cell parameters	a = 10.1353(3) Å	α = 97.774(4)°
	b = 11.9377(5) Å	β = 96.016(3)°
	c = 16.5611(8) Å	γ = 112.238(4)°
Cell volume	1810.90(14) Å^3	
Z	2	
Calculated density	1.749 g/cm^3	
Absorption coefficient μ	3.608 mm^{-1}	
F(000)	944	
Crystal colour and size	colourless, 0.42 × 0.12 × 0.10 mm^3	
Reflections for cell refinement	6521 (θ range 3.4 to 28.5°)	
Data collection method	Agilent Technologies Gemini A Ultra	
	ω scans	
θ range for data collection	3.4 to 28.5°	
Index ranges	h −11 to 12, k −14 to 12, l −15 to 20	
Completeness to θ = 25.2°	99.6 %	
Reflections collected	15057	
Independent reflections	7542 (R_{int} = 0.0438)	
Reflections with F^2>2σ	6893	
Absorption correction	multi-scan	
Min. and max. transmission	0.310 and 0.715	
Structure solution	direct methods	
Refinement method	Full-matrix least-squares on F^2	
Weighting parameters a, b	0.0341, 5.7731	
Data / restraints / parameters	7542 / 339 / 525	

Final R indices [$F^2 > 2\sigma$]	R1 = 0.0376, wR2 = 0.0874
R indices (all data)	R1 = 0.0428, wR2 = 0.0924
Goodness-of-fit on F^2	1.052
Extinction coefficient	0.0008(3)
Largest and mean shift/su	0.002 and 0.000
Largest diff. peak and hole	1.79 and −1.88 e $Å^{-3}$

Table 4:2. Atomic coordinates and equivalent isotropic displacement parameters (Å^2) for ljh120152 (**4**). U_{eq} is defined as one third of the trace of the orthogonalised U^{ij} tensor.

	x	y	z	U_{eq}
Re	0.71965(2)	0.52009(2)	0.31539(2)	0.01709(8)
P1	0.50618(13)	0.54680(11)	0.24962(8)	0.0200(3)
P2	0.75272(13)	0.48298(11)	0.17240(8)	0.0204(3)
P3	0.60582(13)	0.29283(11)	0.28611(8)	0.0176(2)
C1	0.4730(5)	0.6788(5)	0.2976(4)	0.0261(11)
C2	0.4322(7)	0.7487(6)	0.2470(4)	0.0430(16)
C3	0.3978(8)	0.8442(6)	0.2822(5)	0.0537(19)
C4	0.4070(7)	0.8722(5)	0.3670(4)	0.0382(14)
C5	0.4535(8)	0.8070(6)	0.4156(4)	0.0465(16)
C6	0.4865(7)	0.7103(6)	0.3804(4)	0.0376(14)
C7	0.3255(5)	0.4223(4)	0.2291(3)	0.0217(10)
C8	0.2640(5)	0.3498(5)	0.1509(4)	0.0274(11)
C9	0.1279(6)	0.2556(5)	0.1379(4)	0.0315(12)
C10	0.0518(6)	0.2323(5)	0.2030(4)	0.0302(12)
C11	0.1113(6)	0.3058(5)	0.2798(4)	0.0310(12)
C12	0.2478(6)	0.3997(5)	0.2933(3)	0.0279(11)
C13	0.5456(5)	0.5816(5)	0.1480(3)	0.0246(11)
C14	0.6102(5)	0.5001(5)	0.1031(3)	0.0245(11)
C15	0.9180(5)	0.5735(5)	0.1384(3)	0.0245(11)
C16	0.9402(6)	0.6917(5)	0.1278(4)	0.0339(13)
C17	1.0619(7)	0.7628(6)	0.0983(4)	0.0400(15)
C18	1.1626(6)	0.7163(6)	0.0806(4)	0.0399(15)
C19	1.1418(7)	0.5987(7)	0.0909(5)	0.0491(17)
C20	1.0195(6)	0.5265(6)	0.1205(4)	0.0382(14)
C21	0.7223(6)	0.3203(4)	0.1427(3)	0.0250(11)
C22	0.5892(5)	0.2423(4)	0.1746(3)	0.0230(10)
C23	0.7107(5)	0.2184(4)	0.3369(3)	0.0208(10)
C24	0.7175(5)	0.1116(5)	0.2973(4)	0.0266(11)
C25	0.7865(6)	0.0502(5)	0.3400(4)	0.0340(13)

C26	0.8456(6)	0.0948(6)	0.4224(4)	0.0354(13)
C27	0.8390(6)	0.2007(6)	0.4622(4)	0.0347(13)
C28	0.7740(6)	0.2637(5)	0.4192(4)	0.0316(12)
C29	0.4277(5)	0.2079(4)	0.3110(3)	0.0213(10)
C30	0.4027(6)	0.2343(5)	0.3905(4)	0.0289(12)
C31	0.2770(6)	0.1649(5)	0.4149(4)	0.0335(13)
C32	0.1719(6)	0.0671(5)	0.3595(4)	0.0325(13)
C33	0.1931(6)	0.0405(5)	0.2806(4)	0.0343(13)
C34	0.3201(5)	0.1097(5)	0.2552(4)	0.0280(12)
C35	0.9072(5)	0.5160(4)	0.3529(3)	0.0251(11)
O1	1.0206(4)	0.5223(4)	0.3758(3)	0.0429(11)
C36	0.6787(5)	0.5314(4)	0.4292(4)	0.0233(11)
O2	0.6610(5)	0.5385(4)	0.4949(3)	0.0368(9)
C37	0.8185(5)	0.6990(5)	0.3297(3)	0.0276(12)
O3	0.8787(4)	0.8019(3)	0.3388(3)	0.0463(12)
C38	0.2658(7)	0.0235(7)	−0.0240(4)	0.0603(17)
S	0.3575(3)	0.1357(2)	−0.07082(15)	0.0810(7)
F1	0.2956(15)	0.0424(14)	0.0554(5)	0.089(5)
F2	0.1204(9)	0.0082(13)	−0.0358(10)	0.110(5)
F3	0.2557(19)	−0.0856(9)	−0.0576(10)	0.076(5)
O4	0.4990(10)	0.1480(17)	−0.0615(10)	0.105(5)
O5	0.3408(15)	0.2456(7)	−0.0164(6)	0.065(3)
O6	0.2651(12)	0.1089(10)	−0.1504(6)	0.045(3)
F1'	0.3473(14)	0.0178(11)	0.0472(6)	0.076(4)
F2'	0.1521(14)	0.0288(12)	0.0044(10)	0.098(4)
F3'	0.2226(15)	−0.0902(7)	−0.0668(8)	0.050(3)
O4'	0.4624(10)	0.0732(11)	−0.1054(8)	0.071(3)
O5'	0.4462(17)	0.2404(8)	−0.0171(6)	0.099(5)
O6'	0.2469(17)	0.1223(16)	−0.1382(8)	0.121(7)

Table 4:3. Bond lengths [Å] and angles [°] for ljh120152 (**4**).

Re–P1	2.4680(12)	Re–P2	2.4313(13)
Re–P3	2.4666(12)	Re–C35	1.957(5)
Re–C36	1.971(6)	Re–C37	1.954(6)
P1–C1	1.829(5)	P1–C7	1.831(5)
P1–C13	1.835(5)	P2–C14	1.840(5)
P2–C15	1.812(5)	P2–C21	1.834(5)
P3–C22	1.833(5)	P3–C23	1.831(5)
P3–C29	1.828(5)	C1–C2	1.395(8)
C1–C6	1.351(8)	C2–H2	0.950
C2–C3	1.387(9)	C3–H3	0.950
C3–C4	1.384(10)	C4–H4	0.950
C4–C5	1.358(9)	C5–H5	0.950
C5–C6	1.391(8)	C6–H6	0.950
C7–C8	1.390(7)	C7–C12	1.384(7)
C8–H8	0.950	C8–C9	1.382(7)
C9–H9	0.950	C9–C10	1.389(8)
C10–H10	0.950	C10–C11	1.374(8)
C11–H11	0.950	C11–C12	1.383(7)
C12–H12	0.950	C13–H13A	0.990
C13–H13B	0.990	C13–C14	1.522(7)
C14–H14A	0.990	C14–H14B	0.990
C15–C16	1.381(8)	C15–C20	1.384(8)
C16–H16	0.950	C16–C17	1.387(8)
C17–H17	0.950	C17–C18	1.372(9)
C18–H18	0.950	C18–C19	1.375(10)
C19–H19	0.950	C19–C20	1.397(9)
C20–H20	0.950	C21–H21A	0.990
C21–H21B	0.990	C21–C22	1.515(7)
C22–H22A	0.990	C22–H22B	0.990
C23–C24	1.383(7)	C23–C28	1.385(8)
C24–H24	0.950	C24–C25	1.397(7)
C25–H25	0.950	C25–C26	1.377(9)

C26–H26	0.950	C26–C27	1.372(8)
C27–H27	0.950	C27–C28	1.389(8)
C28–H28	0.950	C29–C30	1.385(8)
C29–C34	1.394(7)	C30–H30	0.950
C30–C31	1.368(7)	C31–H31	0.950
C31–C32	1.380(8)	C32–H32	0.950
C32–C33	1.361(9)	C33–H33	0.950
C33–C34	1.385(8)	C34–H34	0.950
C35–O1	1.143(6)	C36–O2	1.117(7)
C37–O3	1.126(6)	C38–S	1.659(9)
C38–F1	1.287(10)	C38–F2	1.404(10)
C38–F3	1.307(10)	C38–F1'	1.391(10)
C38–F2'	1.309(10)	C38–F3'	1.327(9)
S–O4	1.375(9)	S–O5	1.562(9)
S–O6	1.455(8)	S–O4'	1.622(9)
S–O5'	1.358(8)	S–O6'	1.442(9)

P1–Re–P2	81.88(4)	P1–Re–P3	95.12(4)
P1–Re–C35	169.49(16)	P1–Re–C36	97.48(14)
P1–Re–C37	85.76(15)	P2–Re–P3	80.92(4)
P2–Re–C35	90.22(16)	P2–Re–C36	173.86(14)
P2–Re–C37	94.06(17)	P3–Re–C35	90.43(15)
P3–Re–C36	93.08(14)	P3–Re–C37	174.70(16)
C35–Re–C36	91.1(2)	C35–Re–C37	87.9(2)
C36–Re–C37	92.0(2)	Re–P1–C1	116.81(17)
Re–P1–C7	121.69(16)	Re–P1–C13	105.25(17)
C1–P1–C7	102.4(2)	C1–P1–C13	103.4(2)
C7–P1–C13	105.4(2)	Re–P2–C14	110.32(17)
Re–P2–C15	121.59(17)	Re–P2–C21	108.37(17)
C14–P2–C15	103.8(2)	C14–P2–C21	104.1(2)
C15–P2–C21	107.3(2)	Re–P3–C22	107.24(16)
Re–P3–C23	114.67(16)	Re–P3–C29	121.87(16)
C22–P3–C23	106.3(2)	C22–P3–C29	105.3(2)
C23–P3–C29	100.2(2)	P1–C1–C2	119.0(4)

P1–C1–C6	121.9(4)	C2–C1–C6	119.1(5)
C1–C2–H2	120.2	C1–C2–C3	119.5(6)
H2–C2–C3	120.2	C2–C3–H3	119.8
C2–C3–C4	120.5(6)	H3–C3–C4	119.8
C3–C4–H4	120.4	C3–C4–C5	119.2(6)
H4–C4–C5	120.4	C4–C5–H5	119.8
C4–C5–C6	120.4(6)	H5–C5–C6	119.8
C1–C6–C5	121.2(6)	C1–C6–H6	119.4
C5–C6–H6	119.4	P1–C7–C8	122.0(4)
P1–C7–C12	119.0(4)	C8–C7–C12	119.0(5)
C7–C8–H8	119.8	C7–C8–C9	120.4(5)
H8–C8–C9	119.8	C8–C9–H9	119.9
C8–C9–C10	120.2(5)	H9–C9–C10	119.9
C9–C10–H10	120.3	C9–C10–C11	119.3(5)
H10–C10–C11	120.3	C10–C11–H11	119.7
C10–C11–C12	120.7(5)	H11–C11–C12	119.7
C7–C12–C11	120.4(5)	C7–C12–H12	119.8
C11–C12–H12	119.8	P1–C13–H13A	108.8
P1–C13–H13B	108.8	P1–C13–C14	113.7(3)
H13A–C13–H13B	107.7	H13A–C13–C14	108.8
H13B–C13–C14	108.8	P2–C14–C13	111.7(4)
P2–C14–H14A	109.3	P2–C14–H14B	109.3
C13–C14–H14A	109.3	C13–C14–H14B	109.3
H14A–C14–H14B	107.9	P2–C15–C16	118.4(4)
P2–C15–C20	122.1(4)	C16–C15–C20	119.5(5)
C15–C16–H16	119.7	C15–C16–C17	120.6(6)
H16–C16–C17	119.7	C16–C17–H17	120.0
C16–C17–C18	119.9(6)	H17–C17–C18	120.0
C17–C18–H18	120.0	C17–C18–C19	119.9(6)
H18–C18–C19	120.0	C18–C19–H19	119.7
C18–C19–C20	120.5(6)	H19–C19–C20	119.7
C15–C20–C19	119.5(6)	C15–C20–H20	120.3
C19–C20–H20	120.3	P2–C21–H21A	110.0
P2–C21–H21B	110.0	P2–C21–C22	108.6(3)

H21A–C21–H21B	108.3	H21A–C21–C22	110.0
H21B–C21–C22	110.0	P3–C22–C21	111.0(3)
P3–C22–H22A	109.4	P3–C22–H22B	109.4
C21–C22–H22A	109.4	C21–C22–H22B	109.4
H22A–C22–H22B	108.0	P3–C23–C24	121.6(4)
P3–C23–C28	119.3(4)	C24–C23–C28	118.9(5)
C23–C24–H24	119.8	C23–C24–C25	120.4(5)
H24–C24–C25	119.8	C24–C25–H25	120.1
C24–C25–C26	119.9(5)	H25–C25–C26	120.1
C25–C26–H26	119.9	C25–C26–C27	120.1(5)
H26–C26–C27	119.9	C26–C27–H27	120.0
C26–C27–C28	120.0(6)	H27–C27–C28	120.0
C23–C28–C27	120.7(5)	C23–C28–H28	119.7
C27–C28–H28	119.7	P3–C29–C30	118.9(4)
P3–C29–C34	122.5(4)	C30–C29–C34	118.4(5)
C29–C30–H30	119.4	C29–C30–C31	121.3(5)
H30–C30–C31	119.4	C30–C31–H31	120.1
C30–C31–C32	119.8(5)	H31–C31–C32	120.1
C31–C32–H32	120.0	C31–C32–C33	119.9(5)
H32–C32–C33	120.0	C32–C33–H33	119.6
C32–C33–C34	120.8(5)	H33–C33–C34	119.6
C29–C34–C33	119.8(5)	C29–C34–H34	120.1
C33–C34–H34	120.1	Re–C35–O1	175.3(4)
Re–C36–O2	177.2(4)	Re–C37–O3	178.2(5)
S–C38–F1	117.6(7)	S–C38–F2	108.4(8)
S–C38–F3	114.2(8)	S–C38–F1'	113.0(7)
S–C38–F2'	117.6(7)	S–C38–F3'	115.7(7)
F1–C38–F2	101.1(8)	F1–C38–F3	111.6(10)
F2–C38–F3	101.7(9)	F1'–C38–F2'	101.3(8)
F1'–C38–F3'	101.5(8)	F2'–C38–F3'	105.6(9)
C38–S–O4	108.4(6)	C38–S–O5	98.8(4)
C38–S–O6	103.7(5)	C38–S–O4'	96.9(4)
C38–S–O5'	113.0(5)	C38–S–O6'	100.5(7)
O4–S–O5	111.6(7)	O4–S–O6	124.0(8)

O5–S–O6	107.1(6)	O4'–S–O5'	105.6(7)
O4'–S–O6'	108.8(7)	O5'–S–O6'	127.8(8)

Table 4:4. Torsion angles [°] for ljh120152 (**4**).

Re–P1–C1–C2	137.3(4)	Re–P1–C1–C6	–42.8(5)
C7–P1–C1–C2	–87.2(5)	C7–P1–C1–C6	92.7(5)
C13–P1–C1–C2	22.2(5)	C13–P1–C1–C6	–157.9(5)
P1–C1–C2–C3	175.9(5)	C6–C1–C2–C3	–4.0(9)
C1–C2–C3–C4	1.6(11)	C2–C3–C4–C5	1.5(11)
C3–C4–C5–C6	–2.3(11)	P1–C1–C6–C5	–176.6(5)
C2–C1–C6–C5	3.3(9)	C4–C5–C6–C1	–0.2(10)
Re–P1–C7–C8	–100.7(4)	Re–P1–C7–C12	79.3(4)
C1–P1–C7–C8	126.6(4)	C1–P1–C7–C12	–53.5(5)
C13–P1–C7–C8	18.7(5)	C13–P1–C7–C12	–161.4(4)
P1–C7–C8–C9	179.2(4)	C12–C7–C8–C9	–0.7(8)
C7–C8–C9–C10	–0.3(8)	C8–C9–C10–C11	1.8(8)
C9–C10–C11–C12	–2.2(8)	C10–C11–C12–C7	1.1(8)
P1–C7–C12–C11	–179.6(4)	C8–C7–C12–C11	0.3(8)
Re–P1–C13–C14	43.9(4)	C1–P1–C13–C14	167.0(4)
C7–P1–C13–C14	–85.9(4)	P1–C13–C14–P2	–44.7(4)
Re–P2–C14–C13	23.6(4)	C15–P2–C14–C13	–108.2(4)
C21–P2–C14–C13	139.7(4)	Re–P2–C15–C16	–76.0(5)
Re–P2–C15–C20	106.0(5)	C14–P2–C15–C16	48.8(5)
C14–P2–C15–C20	–129.2(5)	C21–P2–C15–C16	158.6(5)
C21–P2–C15–C20	–19.4(6)	P2–C15–C16–C17	–177.1(5)
C20–C15–C16–C17	1.0(9)	C15–C16–C17–C18	–1.0(9)
C16–C17–C18–C19	0.9(10)	C17–C18–C19–C20	–0.8(10)
P2–C15–C20–C19	177.1(5)	C16–C15–C20–C19	–0.9(9)
C18–C19–C20–C15	0.8(10)	Re–P2–C21–C22	43.9(4)
C14–P2–C21–C22	–73.6(4)	C15–P2–C21–C22	176.8(3)
P2–C21–C22–P3	–54.5(4)	Re–P3–C22–C21	39.8(4)
C23–P3–C22–C21	–83.3(4)	C29–P3–C22–C21	171.0(3)
Re–P3–C23–C24	–141.5(4)	Re–P3–C23–C28	44.3(5)
C22–P3–C23–C24	–23.2(5)	C22–P3–C23–C28	162.6(4)

C29–P3–C23–C24	86.2(4)	C29–P3–C23–C28	−88.0(4)
P3–C23–C24–C25	−173.8(4)	C28–C23–C24–C25	0.4(8)
C23–C24–C25–C26	1.3(8)	C24–C25–C26–C27	−1.2(9)
C25–C26–C27–C28	−0.6(9)	P3–C23–C28–C27	172.1(4)
C24–C23–C28–C27	−2.2(8)	C26–C27–C28–C23	2.3(9)
Re–P3–C29–C30	−52.6(5)	Re–P3–C29–C34	133.5(4)
C22–P3–C29–C30	−174.7(4)	C22–P3–C29–C34	11.4(5)
C23–P3–C29–C30	75.1(4)	C23–P3–C29–C34	−98.8(4)
P3–C29–C30–C31	−173.0(4)	C34–C29–C30–C31	1.2(8)
C29–C30–C31–C32	−0.5(9)	C30–C31–C32–C33	−0.5(9)
C31–C32–C33–C34	0.8(9)	C32–C33–C34–C29	−0.1(8)
P3–C29–C34–C33	173.0(4)	C30–C29–C34–C33	−0.9(8)
F1–C38–S–O4	68.1(11)	F1–C38–S–O5	−48.3(10)
F1–C38–S–O6	−158.4(10)	F2–C38–S–O4	−178.1(10)
F2–C38–S–O5	65.5(8)	F2–C38–S–O6	−44.6(9)
F3–C38–S–O4	−65.5(12)	F3–C38–S–O5	178.1(10)
F3–C38–S–O6	68.0(11)	F1'–C38–S–O4'	71.6(8)
F1'–C38–S–O5'	−38.7(11)	F1'–C38–S–O6'	−177.8(10)
F2'–C38–S–O4'	−170.9(9)	F2'–C38–S–O5'	78.8(11)
F2'–C38–S–O6'	−60.3(11)	F3'–C38–S–O4'	−44.8(9)
F3'–C38–S–O5'	−155.1(11)	F3'–C38–S–O6'	65.8(11)

Table 4:5. Anisotropic displacement parameters (Å^2) for ljh120152 (**4**). The anisotropic displacement factor exponent takes the form: $-2\pi^2[h^2a^{*2}U^{11} + ...+ 2hka^*b^*U^{12}]$

	U^{11}	U^{22}	U^{33}	U^{23}	U^{13}	U^{12}
Re	0.01755(12)	0.01204(11)	0.02008(12)	−0.00119(7)	0.00199(7)	0.00585(8)
P1	0.0196(6)	0.0144(6)	0.0245(7)	−0.0003(5)	0.0010(5)	0.0073(5)
P2	0.0214(6)	0.0162(6)	0.0225(7)	0.0012(5)	0.0049(5)	0.0067(5)
P3	0.0205(6)	0.0128(6)	0.0189(7)	−0.0007(5)	0.0031(5)	0.0073(5)
C1	0.022(3)	0.019(3)	0.036(3)	−0.002(2)	0.001(2)	0.010(2)
C2	0.059(4)	0.039(4)	0.036(4)	−0.005(3)	−0.006(3)	0.034(3)
C3	0.076(5)	0.043(4)	0.052(5)	−0.002(3)	−0.008(4)	0.043(4)
C4	0.046(4)	0.027(3)	0.046(4)	−0.001(3)	0.006(3)	0.023(3)
C5	0.075(5)	0.048(4)	0.031(4)	0.000(3)	0.014(3)	0.043(4)
C6	0.055(4)	0.043(4)	0.033(3)	0.009(3)	0.012(3)	0.037(3)
C7	0.019(2)	0.017(2)	0.029(3)	0.000(2)	0.0024(19)	0.0087(19)
C8	0.023(3)	0.023(3)	0.036(3)	0.000(2)	0.006(2)	0.010(2)
C9	0.025(3)	0.026(3)	0.035(3)	−0.009(2)	−0.002(2)	0.007(2)
C10	0.022(3)	0.028(3)	0.039(3)	0.003(2)	0.002(2)	0.009(2)
C11	0.025(3)	0.028(3)	0.041(4)	0.006(2)	0.010(2)	0.011(2)
C12	0.029(3)	0.027(3)	0.025(3)	−0.003(2)	0.001(2)	0.013(2)
C13	0.025(3)	0.019(2)	0.029(3)	0.008(2)	0.003(2)	0.008(2)
C14	0.023(3)	0.024(3)	0.022(3)	0.002(2)	0.0021(19)	0.006(2)
C15	0.026(3)	0.023(3)	0.021(3)	0.004(2)	0.0059(19)	0.006(2)
C16	0.028(3)	0.029(3)	0.042(4)	0.006(3)	0.016(2)	0.006(2)
C17	0.041(3)	0.036(3)	0.034(4)	0.006(3)	0.014(3)	0.004(3)
C18	0.025(3)	0.054(4)	0.028(3)	0.007(3)	0.006(2)	0.002(3)
C19	0.027(3)	0.065(5)	0.060(5)	0.015(4)	0.011(3)	0.023(3)
C20	0.028(3)	0.040(3)	0.050(4)	0.012(3)	0.009(3)	0.015(3)
C21	0.034(3)	0.021(3)	0.022(3)	−0.001(2)	0.007(2)	0.014(2)
C22	0.027(3)	0.015(2)	0.023(3)	−0.003(2)	0.0016(19)	0.008(2)
C23	0.021(2)	0.017(2)	0.026(3)	0.004(2)	0.0070(19)	0.0086(19)
C24	0.030(3)	0.020(3)	0.032(3)	0.001(2)	0.007(2)	0.012(2)

C25	0.044(3)	0.028(3)	0.040(4)	0.005(3)	0.007(3)	0.027(3)
C26	0.037(3)	0.041(3)	0.043(4)	0.019(3)	0.013(3)	0.026(3)
C27	0.040(3)	0.043(3)	0.028(3)	0.007(3)	0.003(2)	0.025(3)
C28	0.040(3)	0.027(3)	0.031(3)	0.000(2)	0.004(2)	0.019(2)
C29	0.019(2)	0.016(2)	0.029(3)	0.003(2)	0.0027(19)	0.0083(19)
C30	0.030(3)	0.018(3)	0.034(3)	−0.001(2)	0.009(2)	0.005(2)
C31	0.035(3)	0.025(3)	0.037(3)	0.001(2)	0.016(2)	0.007(2)
C32	0.028(3)	0.021(3)	0.050(4)	0.008(3)	0.015(2)	0.009(2)
C33	0.022(3)	0.023(3)	0.048(4)	−0.003(3)	0.000(2)	0.004(2)
C34	0.028(3)	0.020(3)	0.032(3)	−0.002(2)	0.002(2)	0.007(2)
C35	0.025(3)	0.016(2)	0.031(3)	−0.005(2)	0.005(2)	0.008(2)
O1	0.028(2)	0.039(2)	0.058(3)	−0.004(2)	−0.0040(19)	0.0178(19)
C36	0.019(2)	0.014(2)	0.036(3)	0.005(2)	0.001(2)	0.0071(19)
O2	0.056(3)	0.036(2)	0.022(2)	0.0023(18)	0.0129(18)	0.022(2)
C37	0.022(3)	0.030(3)	0.031(3)	0.005(2)	0.004(2)	0.012(2)
O3	0.036(2)	0.012(2)	0.078(4)	0.001(2)	0.002(2)	0.0008(17)
C38	0.064(4)	0.059(3)	0.047(3)	−0.015(3)	0.005(3)	0.021(3)
S	0.0854(16)	0.0723(15)	0.0567(14)	0.0174(11)	−0.0014(11)	0.0018(12)
F1	0.078(8)	0.090(9)	0.044(4)	−0.015(4)	0.020(4)	−0.022(6)
F2	0.069(5)	0.105(9)	0.117(10)	−0.050(8)	−0.015(5)	0.025(5)
F3	0.111(12)	0.066(6)	0.046(7)	−0.013(5)	−0.018(7)	0.046(6)
O4	0.054(5)	0.161(14)	0.087(11)	0.061(10)	0.001(5)	0.020(6)
O5	0.085(8)	0.035(5)	0.037(6)	−0.001(4)	−0.004(5)	−0.010(5)
O6	0.080(6)	0.027(5)	0.027(4)	0.004(4)	0.007(4)	0.022(5)
F1'	0.098(8)	0.054(6)	0.047(5)	0.009(4)	−0.002(5)	0.001(5)
F2'	0.126(8)	0.108(9)	0.100(9)	0.040(7)	0.072(7)	0.070(7)
F3'	0.052(5)	0.046(4)	0.040(5)	−0.005(4)	−0.015(4)	0.018(4)
O4'	0.048(5)	0.074(7)	0.062(7)	0.020(5)	0.007(5)	−0.007(5)
O5'	0.149(11)	0.041(5)	0.041(6)	0.015(4)	−0.035(7)	−0.021(6)
O6'	0.155(11)	0.114(12)	0.082(8)	−0.039(7)	−0.048(8)	0.076(9)

Table 4:6. Hydrogen coordinates and isotropic displacement parameters (Å^2) for ljh120152 (**4**).

	x	y	z	U
H2	0.4281	0.7311	0.1889	0.052
H3	0.3676	0.8906	0.2478	0.064
H4	0.3812	0.9363	0.3909	0.046
H5	0.4636	0.8275	0.4741	0.056
H6	0.5192	0.6657	0.4153	0.045
H8	0.3158	0.3650	0.1062	0.033
H9	0.0864	0.2067	0.0843	0.038
H10	−0.0406	0.1662	0.1945	0.036
H11	0.0583	0.2920	0.3241	0.037
H12	0.2884	0.4488	0.3469	0.033
H13A	0.4549	0.5717	0.1131	0.030
H13B	0.6138	0.6688	0.1553	0.030
H14A	0.6512	0.5366	0.0565	0.029
H14B	0.5327	0.4178	0.0800	0.029
H16	0.8715	0.7246	0.1409	0.041
H17	1.0754	0.8435	0.0905	0.048
H18	1.2466	0.7653	0.0611	0.048
H19	1.2110	0.5665	0.0779	0.059
H20	1.0062	0.4458	0.1283	0.046
H21A	0.7079	0.2969	0.0818	0.030
H21B	0.8074	0.3067	0.1667	0.030
H22A	0.5763	0.1549	0.1630	0.028
H22B	0.5028	0.2486	0.1453	0.028
H24	0.6751	0.0797	0.2407	0.032
H25	0.7927	−0.0222	0.3122	0.041
H26	0.8909	0.0522	0.4518	0.042
H27	0.8790	0.2309	0.5192	0.042
H28	0.7730	0.3386	0.4465	0.038

H30	0.4742	0.3019	0.4289	0.035
H31	0.2621	0.1841	0.4699	0.040
H32	0.0849	0.0184	0.3764	0.039
H33	0.1200	−0.0263	0.2425	0.041
H34	0.3339	0.0903	0.2000	0.034

5.5 Crystallographic data for *fac*-[Re(CO)$_3$(2)][OTf] (5)

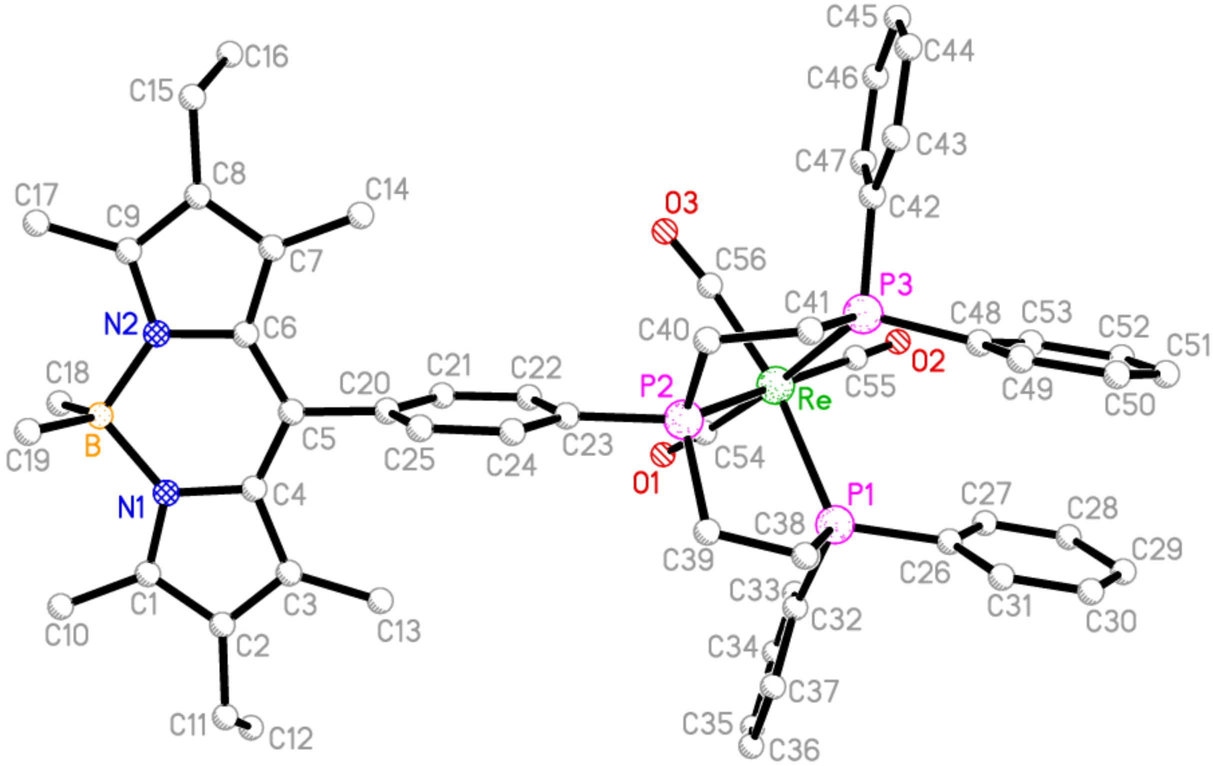

Table 5:1. Crystal data and structure refinement for ljh120041 (**5**).

Identification code	ljh120041 (**5**)	
Chemical formula (moiety)	$C_{56}H_{60}BN_2O_3P_3Re^{+}\cdot CF_3O_3S^{-}\cdot C_7H_8N_2O_2\cdot C_5H_{12}$	
Chemical formula (total)	$C_{69}H_{80}BF_3N_4O_8P_3ReS$	
Formula weight	1472.35	
Temperature	150(2) K	
Radiation, wavelength	CuKα, 1.54178 Å	
Crystal system, space group	triclinic, $P\overline{1}$	
Unit cell parameters	a = 11.7574(5) Å	α = 92.712(2)°
	b = 16.2273(5) Å	β = 97.724(3)°
	c = 18.3187(6) Å	γ = 98.344(3)°
Cell volume	3418.7(2) $Å^3$	
Z	2	
Calculated density	1.430 g/cm^3	
Absorption coefficient μ	4.957 mm^{-1}	
F(000)	1508	
Crystal colour and size	red, 0.30 × 0.20 × 0.20 mm^3	
Reflections for cell refinement	15771 (θ range 2.4 to 66.7°)	
Data collection method	Agilent Technologies Gemini A Ultra	
	ω scans	
θ range for data collection	2.4 to 66.8°	
Index ranges	h −13 to 13, k −19 to 17, l −15 to 21	
Completeness to θ = 67.7°	95.5 %	
Reflections collected	23448	
Independent reflections	11816 (R_{int} = 0.0791)	
Reflections with F^2>2σ	11250	
Absorption correction	multi-scan	
Min. and max. transmission	0.320 and 1.440	
Structure solution	direct methods	
Refinement method	Full-matrix least-squares on F^2	
Weighting parameters a, b	0.1725, 3.1808	
Data / restraints / parameters	11816 / 151 / 839	

Final R indices [$F^2 > 2\sigma$]	R1 = 0.0732, wR2 = 0.1994
R indices (all data)	R1 = 0.0748, wR2 = 0.2032
Goodness-of-fit on F^2	1.027
Largest and mean shift/su	0.001 and 0.000
Largest diff. peak and hole	5.94 and −3.21 e Å^{-3}

Table 5:2. Atomic coordinates and equivalent isotropic displacement parameters (Å^2) for ljh120041 (**5**). U_{eq} is defined as one third of the trace of the orthogonalised U^{ij} tensor.

	x	y	z	U_{eq}
Re	0.19020(2)	0.12846(2)	0.35199(2)	0.01900(14)
B	0.7285(6)	0.1677(4)	0.8763(4)	0.0264(13)
N1	0.7687(4)	0.1856(3)	0.7985(3)	0.0285(10)
N2	0.5911(4)	0.1546(3)	0.8617(2)	0.0263(10)
C1	0.8783(5)	0.2002(4)	0.7838(3)	0.0323(13)
C2	0.8801(5)	0.2186(4)	0.7088(3)	0.0327(13)
C3	0.7664(5)	0.2159(4)	0.6772(3)	0.0330(13)
C4	0.6952(5)	0.1952(4)	0.7338(3)	0.0266(12)
C5	0.5768(5)	0.1873(3)	0.7333(3)	0.0259(11)
C6	0.5229(5)	0.1672(4)	0.7950(3)	0.0255(11)
C7	0.4046(5)	0.1564(4)	0.8057(3)	0.0291(12)
C8	0.4020(6)	0.1376(4)	0.8788(3)	0.0344(13)
C9	0.5175(5)	0.1372(4)	0.9114(3)	0.0309(13)
C10	0.9843(6)	0.2004(5)	0.8391(4)	0.0406(16)
C11	0.9874(6)	0.2369(5)	0.6730(4)	0.0411(16)
C12	1.0175(7)	0.1588(6)	0.6333(4)	0.0542(19)
C13	0.7280(6)	0.2302(5)	0.5979(3)	0.0439(17)
C14	0.3006(5)	0.1657(5)	0.7524(4)	0.0375(15)
C15	0.2958(6)	0.1197(5)	0.9162(4)	0.0418(15)
C16	0.2290(6)	0.0312(5)	0.8968(4)	0.0480(17)
C17	0.5562(7)	0.1210(5)	0.9901(3)	0.0438(16)
C18	0.7721(6)	0.0817(4)	0.9054(4)	0.0355(14)
C19	0.7715(6)	0.2506(4)	0.9312(3)	0.0349(14)
C20	0.5021(5)	0.1985(4)	0.6625(3)	0.0258(12)
C21	0.4575(5)	0.1303(3)	0.6129(3)	0.0269(12)
C22	0.3914(5)	0.1428(4)	0.5465(3)	0.0259(11)
C23	0.3708(5)	0.2221(4)	0.5287(3)	0.0253(11)
C24	0.4157(5)	0.2899(4)	0.5791(3)	0.0314(13)
C25	0.4799(6)	0.2784(4)	0.6465(3)	0.0329(13)

P1	0.29894(12)	0.21455(9)	0.27022(7)	0.0221(3)
P2	0.28642(12)	0.23933(8)	0.44216(7)	0.0205(3)
P3	0.03682(12)	0.21926(8)	0.34447(7)	0.0193(3)
C26	0.2301(5)	0.2292(4)	0.1773(3)	0.0288(12)
C27	0.1881(5)	0.1593(4)	0.1294(3)	0.0346(13)
C28	0.1386(6)	0.1672(5)	0.0576(4)	0.0419(16)
C29	0.1314(6)	0.2474(5)	0.0329(4)	0.0457(17)
C30	0.1726(7)	0.3161(6)	0.0807(4)	0.0467(18)
C31	0.2226(6)	0.3087(4)	0.1532(3)	0.0342(13)
C32	0.4348(5)	0.1823(4)	0.2516(3)	0.0259(11)
C33	0.4342(5)	0.0991(4)	0.2290(3)	0.0330(13)
C34	0.5325(6)	0.0725(4)	0.2071(4)	0.0387(14)
C35	0.6332(6)	0.1287(5)	0.2091(4)	0.0405(15)
C36	0.6348(6)	0.2115(5)	0.2320(4)	0.0383(15)
C37	0.5363(5)	0.2386(4)	0.2528(3)	0.0307(13)
C38	0.3403(5)	0.3196(3)	0.3169(3)	0.0262(11)
C39	0.3846(5)	0.3141(3)	0.3992(3)	0.0267(12)
C40	0.1744(5)	0.2952(4)	0.4728(3)	0.0266(11)
C41	0.0895(5)	0.3141(3)	0.4071(3)	0.0255(11)
C42	−0.0998(5)	0.1736(3)	0.3742(3)	0.0231(11)
C43	−0.1764(5)	0.2244(4)	0.3966(3)	0.0277(12)
C44	−0.2827(5)	0.1892(4)	0.4144(3)	0.0331(13)
C45	−0.3145(5)	0.1038(4)	0.4101(3)	0.0370(14)
C46	−0.2398(6)	0.0525(4)	0.3873(4)	0.0363(15)
C47	−0.1331(5)	0.0876(4)	0.3694(3)	0.0289(12)
C48	−0.0176(4)	0.2587(3)	0.2572(3)	0.0219(10)
C49	−0.0304(5)	0.3428(4)	0.2515(3)	0.0276(12)
C50	−0.0816(5)	0.3690(4)	0.1851(3)	0.0317(13)
C51	−0.1191(5)	0.3128(4)	0.1247(3)	0.0335(13)
C52	−0.1069(5)	0.2301(4)	0.1303(3)	0.0304(12)
C53	−0.0558(5)	0.2028(4)	0.1963(3)	0.0278(12)
C54	0.3109(5)	0.0600(3)	0.3674(3)	0.0239(11)
O1	0.3788(4)	0.0164(3)	0.3792(3)	0.0441(11)

C55	0.1003(5)	0.0476(4)	0.2739(3)	0.0281(13)
O2	0.0487(4)	0.0014(3)	0.2305(3)	0.0402(11)
C56	0.1275(5)	0.0742(4)	0.4335(3)	0.0286(12)
O3	0.1007(4)	0.0443(3)	0.4856(3)	0.0455(12)
C57	−0.1903(8)	0.5098(8)	0.3882(7)	0.085(4)
F1	−0.1004(5)	0.5305(8)	0.4440(6)	0.155(4)
F2	−0.2040(7)	0.5792(5)	0.3610(4)	0.116(3)
F3	−0.1573(8)	0.4642(7)	0.3376(6)	0.165(5)
S	−0.31634(16)	0.45943(10)	0.42436(11)	0.0444(4)
O4	−0.3382(5)	0.5215(3)	0.4754(3)	0.0569(15)
O5	−0.3977(6)	0.4402(5)	0.3606(4)	0.087(2)
O6	−0.2819(11)	0.3880(6)	0.4605(6)	0.125(4)
C58	0.1007(7)	0.5321(5)	0.0928(4)	0.0484(18)
C59	0.1745(8)	0.5402(4)	0.1652(4)	0.0505(19)
C60	0.1292(7)	0.5486(4)	0.2305(4)	0.0470(17)
C61	0.1931(7)	0.5450(4)	0.2990(4)	0.0479(18)
C62	0.3108(7)	0.5334(4)	0.3024(4)	0.0467(17)
C63	0.3586(7)	0.5284(4)	0.2370(4)	0.0467(17)
C64	0.2900(7)	0.5315(5)	0.1692(4)	0.0494(18)
O7	−0.0065(6)	0.5405(4)	0.0913(3)	0.0613(16)
O8	0.1418(6)	0.5151(4)	0.0348(3)	0.0588(15)
N3	0.1462(8)	0.5432(5)	0.3635(4)	0.071(2)
N4	0.3779(7)	0.5282(4)	0.3694(4)	0.0527(17)
C65	0.522(2)	0.3456(15)	1.0745(16)	0.201(9)
C66	0.545(3)	0.386(3)	1.010(2)	0.197(10)
C67	0.411(3)	0.377(5)	0.9599(17)	0.198(10)
C68	0.384(3)	0.366(4)	0.868(2)	0.207(11)
C69	0.275(2)	0.3931(18)	0.8474(17)	0.224(10)
C66'	0.512(5)	0.332(4)	0.9959(19)	0.211(11)
C67'	0.400(4)	0.378(5)	0.961(2)	0.208(10)
C68'	0.391(3)	0.427(3)	0.881(3)	0.209(11)

Table 5:3. Bond lengths [Å] and angles [°] for ljh120041 (**5**).

Re–P1	2.4544(13)	Re–P2	2.4230(14)
Re–P3	2.4831(14)	Re–C54	1.926(6)
Re–C55	1.975(6)	Re–C56	1.944(6)
B–N1	1.588(8)	B–N2	1.583(8)
B–C18	1.641(8)	B–C19	1.616(9)
N1–C1	1.340(8)	N1–C4	1.398(7)
N2–C6	1.409(7)	N2–C9	1.350(8)
C1–C2	1.423(9)	C1–C10	1.496(8)
C2–C3	1.378(9)	C2–C11	1.497(9)
C3–C4	1.441(8)	C3–C13	1.501(8)
C4–C5	1.379(8)	C5–C6	1.397(8)
C5–C20	1.499(7)	C6–C7	1.417(8)
C7–C8	1.390(9)	C7–C14	1.490(8)
C8–C9	1.409(9)	C8–C15	1.502(9)
C9–C17	1.499(8)	C10–H10A	0.980
C10–H10B	0.980	C10–H10C	0.980
C11–H11A	0.990	C11–H11B	0.990
C11–C12	1.540(11)	C12–H12A	0.980
C12–H12B	0.980	C12–H12C	0.980
C13–H13A	0.980	C13–H13B	0.980
C13–H13C	0.980	C14–H14A	0.980
C14–H14B	0.980	C14–H14C	0.980
C15–H15A	0.990	C15–H15B	0.990
C15–C16	1.534(10)	C16–H16A	0.980
C16–H16B	0.980	C16–H16C	0.980
C17–H17A	0.980	C17–H17B	0.980
C17–H17C	0.980	C18–H18A	0.980
C18–H18B	0.980	C18–H18C	0.980
C19–H19A	0.980	C19–H19B	0.980
C19–H19C	0.980	C20–C21	1.391(8)
C20–C25	1.396(9)	C21–H21	0.950
C21–C22	1.394(8)	C22–H22	0.950

C22–C23	1.389(8)	C23–C24	1.395(8)
C23–P2	1.809(5)	C24–H24	0.950
C24–C25	1.394(8)	C25–H25	0.950
P1–C26	1.825(6)	P1–C32	1.821(6)
P1–C38	1.840(6)	P2–C39	1.831(5)
P2–C40	1.836(6)	P3–C41	1.852(6)
P3–C42	1.831(5)	P3–C48	1.820(5)
C26–C27	1.388(9)	C26–C31	1.395(9)
C27–H27	0.950	C27–C28	1.385(9)
C28–H28	0.950	C28–C29	1.407(11)
C29–H29	0.950	C29–C30	1.372(12)
C30–H30	0.950	C30–C31	1.396(9)
C31–H31	0.950	C32–C33	1.392(9)
C32–C37	1.390(8)	C33–H33	0.950
C33–C34	1.391(9)	C34–H34	0.950
C34–C35	1.381(10)	C35–H35	0.950
C35–C36	1.385(11)	C36–H36	0.950
C36–C37	1.389(10)	C37–H37	0.950
C38–H38A	0.990	C38–H38B	0.990
C38–C39	1.538(7)	C39–H39A	0.990
C39–H39B	0.990	C40–H40A	0.990
C40–H40B	0.990	C40–C41	1.530(7)
C41–H41A	0.990	C41–H41B	0.990
C42–C43	1.394(8)	C42–C47	1.390(8)
C43–H43	0.950	C43–C44	1.384(8)
C44–H44	0.950	C44–C45	1.378(10)
C45–H45	0.950	C45–C46	1.384(10)
C46–H46	0.950	C46–C47	1.387(9)
C47–H47	0.950	C48–C49	1.402(8)
C48–C53	1.387(8)	C49–H49	0.950
C49–C50	1.395(8)	C50–H50	0.950
C50–C51	1.381(9)	C51–H51	0.950
C51–C52	1.376(9)	C52–H52	0.950
C52–C53	1.397(8)	C53–H53	0.950

C54–O1	1.147(7)	C55–O2	1.115(7)
C56–O3	1.152(8)	C57–F1	1.359(15)
C57–F2	1.275(16)	C57–F3	1.293(11)
C57–S	1.808(10)	S–O4	1.417(6)
S–O5	1.399(7)	S–O6	1.443(8)
C58–C59	1.474(11)	C58–O7	1.284(10)
C58–O8	1.260(10)	C59–C60	1.382(11)
C59–C64	1.378(12)	C60–H60	0.950
C60–C61	1.383(11)	C61–C62	1.417(12)
C61–N3	1.368(11)	C62–C63	1.394(11)
C62–N4	1.379(10)	C63–H63	0.950
C63–C64	1.395(11)	C64–H64	0.950
O8–H8	0.840	N3–H3A	0.880
N3–H3B	0.880	N4–H4A	0.880
N4–H4B	0.880	C65–H65A	0.980
C65–H65B	0.980	C65–H65C	0.980
C65–H65D	0.980	C65–H65E	0.980
C65–H65F	0.980	C65–C66	1.42(3)
C65–C66'	1.43(3)	C66–H66A	0.990
C66–H66B	0.990	C66–C67	1.69(3)
C67–H67A	0.990	C67–H67B	0.990
C67–C68	1.67(3)	C68–H68A	0.990
C68–H68B	0.990	C68–C69	1.42(3)
C69–H69A	0.980	C69–H69B	0.980
C69–H69C	0.980	C69–H69D	0.980
C69–H69E	0.980	C69–H69F	0.980
C69–C68'	1.43(3)	C66'–H66C	0.990
C66'–H66D	0.990	C66'–C67'	1.68(3)
C67'–H67C	0.990	C67'–H67D	0.990
C67'–C68'	1.69(3)	C68'–H68C	0.990
C68'–H68D	0.990		

P1–Re–P2	81.44(5)	P1–Re–P3	91.64(4)
P1–Re–C54	91.25(17)	P1–Re–C55	96.49(17)

P1–Re–C56	167.69(18)	P2–Re–P3	81.11(4)
P2–Re–C54	95.05(17)	P2–Re–C55	173.27(17)
P2–Re–C56	86.79(18)	P3–Re–C54	174.80(17)
P3–Re–C55	92.58(18)	P3–Re–C56	90.10(17)
C54–Re–C55	91.4(2)	C54–Re–C56	86.2(2)
C55–Re–C56	95.6(2)	N1–B–N2	105.3(4)
N1–B–C18	109.9(5)	N1–B–C19	108.3(5)
N2–B–C18	109.4(5)	N2–B–C19	107.6(5)
C18–B–C19	115.8(5)	B–N1–C1	126.7(5)
B–N1–C4	125.5(5)	C1–N1–C4	107.7(5)
B–N2–C6	125.6(5)	B–N2–C9	127.2(5)
C6–N2–C9	106.9(5)	N1–C1–C2	110.5(5)
N1–C1–C10	125.0(6)	C2–C1–C10	124.5(6)
C1–C2–C3	107.0(5)	C1–C2–C11	125.1(6)
C3–C2–C11	127.9(6)	C2–C3–C4	106.8(5)
C2–C3–C13	124.9(6)	C4–C3–C13	128.2(6)
N1–C4–C3	108.0(5)	N1–C4–C5	120.6(5)
C3–C4–C5	131.4(5)	C4–C5–C6	123.2(5)
C4–C5–C20	118.3(5)	C6–C5–C20	118.5(5)
N2–C6–C5	119.5(5)	N2–C6–C7	108.7(5)
C5–C6–C7	131.8(5)	C6–C7–C8	106.6(5)
C6–C7–C14	128.7(5)	C8–C7–C14	124.6(5)
C7–C8–C9	107.4(5)	C7–C8–C15	126.5(6)
C9–C8–C15	126.0(6)	N2–C9–C8	110.4(5)
N2–C9–C17	123.6(6)	C8–C9–C17	126.0(6)
C1–C10–H10A	109.5	C1–C10–H10B	109.5
C1–C10–H10C	109.5	H10A–C10–H10B	109.5
H10A–C10–H10C	109.5	H10B–C10–H10C	109.5
C2–C11–H11A	109.0	C2–C11–H11B	109.0
C2–C11–C12	112.7(6)	H11A–C11–H11B	107.8
H11A–C11–C12	109.0	H11B–C11–C12	109.0
C11–C12–H12A	109.5	C11–C12–H12B	109.5
C11–C12–H12C	109.5	H12A–C12–H12B	109.5
H12A–C12–H12C	109.5	H12B–C12–H12C	109.5

C3–C13–H13A	109.5	C3–C13–H13B	109.5
C3–C13–H13C	109.5	H13A–C13–H13B	109.5
H13A–C13–H13C	109.5	H13B–C13–H13C	109.5
C7–C14–H14A	109.5	C7–C14–H14B	109.5
C7–C14–H14C	109.5	H14A–C14–H14B	109.5
H14A–C14–H14C	109.5	H14B–C14–H14C	109.5
C8–C15–H15A	108.9	C8–C15–H15B	108.9
C8–C15–C16	113.4(6)	H15A–C15–H15B	107.7
H15A–C15–C16	108.9	H15B–C15–C16	108.9
C15–C16–H16A	109.5	C15–C16–H16B	109.5
C15–C16–H16C	109.5	H16A–C16–H16B	109.5
H16A–C16–H16C	109.5	H16B–C16–H16C	109.5
C9–C17–H17A	109.5	C9–C17–H17B	109.5
C9–C17–H17C	109.5	H17A–C17–H17B	109.5
H17A–C17–H17C	109.5	H17B–C17–H17C	109.5
B–C18–H18A	109.5	B–C18–H18B	109.5
B–C18–H18C	109.5	H18A–C18–H18B	109.5
H18A–C18–H18C	109.5	H18B–C18–H18C	109.5
B–C19–H19A	109.5	B–C19–H19B	109.5
B–C19–H19C	109.5	H19A–C19–H19B	109.5
H19A–C19–H19C	109.5	H19B–C19–H19C	109.5
C5–C20–C21	120.3(5)	C5–C20–C25	119.2(5)
C21–C20–C25	120.6(5)	C20–C21–H21	120.4
C20–C21–C22	119.1(5)	H21–C21–C22	120.4
C21–C22–H22	119.4	C21–C22–C23	121.2(5)
H22–C22–C23	119.4	C22–C23–C24	119.0(5)
C22–C23–P2	121.6(4)	C24–C23–P2	119.3(4)
C23–C24–H24	119.7	C23–C24–C25	120.6(5)
H24–C24–C25	119.7	C20–C25–C24	119.4(5)
C20–C25–H25	120.3	C24–C25–H25	120.3
Re–P1–C26	119.76(19)	Re–P1–C32	116.06(19)
Re–P1–C38	107.03(18)	C26–P1–C32	101.5(3)
C26–P1–C38	106.1(3)	C32–P1–C38	105.2(3)
Re–P2–C23	123.97(19)	Re–P2–C39	110.10(19)

Re–P2–C40	107.71(18)	C23–P2–C39	104.2(3)
C23–P2–C40	102.3(3)	C39–P2–C40	107.4(3)
Re–P3–C41	108.78(18)	Re–P3–C42	115.23(18)
Re–P3–C48	121.15(18)	C41–P3–C42	105.1(3)
C41–P3–C48	104.5(3)	C42–P3–C48	100.5(2)
P1–C26–C27	118.8(5)	P1–C26–C31	121.4(5)
C27–C26–C31	119.8(6)	C26–C27–H27	119.5
C26–C27–C28	121.0(6)	H27–C27–C28	119.5
C27–C28–H28	120.3	C27–C28–C29	119.4(6)
H28–C28–C29	120.3	C28–C29–H29	120.3
C28–C29–C30	119.3(6)	H29–C29–C30	120.4
C29–C30–H30	119.1	C29–C30–C31	121.7(7)
H30–C30–C31	119.1	C26–C31–C30	118.8(7)
C26–C31–H31	120.6	C30–C31–H31	120.6
P1–C32–C33	118.4(4)	P1–C32–C37	122.6(5)
C33–C32–C37	118.8(6)	C32–C33–H33	119.5
C32–C33–C34	120.9(6)	H33–C33–C34	119.5
C33–C34–H34	120.0	C33–C34–C35	119.9(6)
H34–C34–C35	120.0	C34–C35–H35	120.3
C34–C35–C36	119.5(6)	H35–C35–C36	120.3
C35–C36–H36	119.6	C35–C36–C37	120.8(6)
H36–C36–C37	119.6	C32–C37–C36	120.1(6)
C32–C37–H37	120.0	C36–C37–H37	120.0
P1–C38–H38A	109.5	P1–C38–H38B	109.5
P1–C38–C39	110.5(4)	H38A–C38–H38B	108.1
H38A–C38–C39	109.5	H38B–C38–C39	109.5
P2–C39–C38	111.6(4)	P2–C39–H39A	109.3
P2–C39–H39B	109.3	C38–C39–H39A	109.3
C38–C39–H39B	109.3	H39A–C39–H39B	108.0
P2–C40–H40A	109.4	P2–C40–H40B	109.4
P2–C40–C41	111.1(4)	H40A–C40–H40B	108.0
H40A–C40–C41	109.4	H40B–C40–C41	109.4
P3–C41–C40	110.8(4)	P3–C41–H41A	109.5
P3–C41–H41B	109.5	C40–C41–H41A	109.5

C40–C41–H41B	109.5	H41A–C41–H41B	108.1
P3–C42–C43	120.7(4)	P3–C42–C47	120.5(4)
C43–C42–C47	118.7(5)	C42–C43–H43	119.9
C42–C43–C44	120.2(6)	H43–C43–C44	119.9
C43–C44–H44	119.6	C43–C44–C45	120.7(6)
H44–C44–C45	119.6	C44–C45–H45	120.1
C44–C45–C46	119.8(6)	H45–C45–C46	120.1
C45–C46–H46	120.2	C45–C46–C47	119.7(6)
H46–C46–C47	120.2	C42–C47–C46	121.0(6)
C42–C47–H47	119.5	C46–C47–H47	119.5
P3–C48–C49	121.8(4)	P3–C48–C53	119.0(4)
C49–C48–C53	119.1(5)	C48–C49–H49	120.1
C48–C49–C50	119.9(5)	H49–C49–C50	120.1
C49–C50–H50	119.7	C49–C50–C51	120.5(6)
H50–C50–C51	119.7	C50–C51–H51	120.2
C50–C51–C52	119.7(5)	H51–C51–C52	120.2
C51–C52–H52	119.7	C51–C52–C53	120.6(5)
H52–C52–C53	119.7	C48–C53–C52	120.3(5)
C48–C53–H53	119.9	C52–C53–H53	119.9
Re–C54–O1	175.9(5)	Re–C55–O2	179.0(6)
Re–C56–O3	173.5(5)	F1–C57–F2	104.1(10)
F1–C57–F3	108.6(10)	F1–C57–S	109.6(10)
F2–C57–F3	106.8(13)	F2–C57–S	113.7(8)
F3–C57–S	113.4(8)	C57–S–O4	103.0(4)
C57–S–O5	102.2(5)	C57–S–O6	106.5(7)
O4–S–O5	116.5(5)	O4–S–O6	111.9(5)
O5–S–O6	114.8(6)	C59–C58–O7	117.9(7)
C59–C58–O8	120.4(8)	O7–C58–O8	121.7(7)
C58–C59–C60	121.8(8)	C58–C59–C64	119.7(7)
C60–C59–C64	118.2(7)	C59–C60–H60	118.5
C59–C60–C61	122.9(8)	H60–C60–C61	118.5
C60–C61–C62	118.4(7)	C60–C61–N3	123.6(8)
C62–C61–N3	117.6(7)	C61–C62–C63	119.0(7)
C61–C62–N4	120.7(8)	C63–C62–N4	120.3(8)

C62–C63–H63	119.8	C62–C63–C64	120.3(8)
H63–C63–C64	119.9	C59–C64–C63	121.1(8)
C59–C64–H64	119.5	C63–C64–H64	119.5
C58–O8–H8	109.5	C61–N3–H3A	120.0
C61–N3–H3B	120.0	H3A–N3–H3B	120.0
C62–N4–H4A	120.0	C62–N4–H4B	120.0
H4A–N4–H4B	120.0	H65A–C65–H65B	109.5
H65A–C65–H65C	109.5	H65A–C65–C66	109.5
H65B–C65–H65C	109.5	H65B–C65–C66	109.5
H65C–C65–C66	109.5	H65D–C65–H65E	109.5
H65D–C65–H65F	109.5	H65D–C65–C66'	109.5
H65E–C65–H65F	109.5	H65E–C65–C66'	109.5
H65F–C65–C66'	109.5	C65–C66–H66A	111.3
C65–C66–H66B	111.3	C65–C66–C67	102(2)
H66A–C66–H66B	109.2	H66A–C66–C67	111.3
H66B–C66–C67	111.3	C66–C67–H67A	106.2
C66–C67–H67B	106.2	C66–C67–C68	125(3)
H67A–C67–H67B	106.4	H67A–C67–C68	106.2
H67B–C67–C68	106.2	C67–C68–H68A	110.5
C67–C68–H68B	110.5	C67–C68–C69	106(2)
H68A–C68–H68B	108.7	H68A–C68–C69	110.5
H68B–C68–C69	110.5	C68–C69–H69A	109.5
C68–C69–H69B	109.5	C68–C69–H69C	109.5
H69A–C69–H69B	109.5	H69A–C69–H69C	109.5
H69B–C69–H69C	109.5	H69D–C69–H69E	109.5
H69D–C69–H69F	109.5	H69D–C69–C68'	109.5
H69E–C69–H69F	109.5	H69E–C69–C68'	109.5
H69F–C69–C68'	109.5	C65–C66'–H66C	110.6
C65–C66'–H66D	110.6	C65–C66'–C67'	106(2)
H66C–C66'–H66D	108.7	H66C–C66'–C67'	110.6
H66D–C66'–C67'	110.6	C66'–C67'–H67C	106.0
C66'–C67'–H67D	106.0	C66'–C67'–C68'	125(3)
H67C–C67'–H67D	106.3	H67C–C67'–C68'	106.0
H67D–C67'–C68'	106.0	C69–C68'–C67'	102(2)

C69–C68'–H68C	111.4	C69–C68'–H68D	111.4
C67'–C68'–H68C	111.4	C67'–C68'–H68D	111.4
H68C–C68'–H68D	109.3		

Table 5:4. Torsion angles [°] for ljh120041 (**5**).

N2–B–N1–C1	−179.7(5)	N2–B–N1–C4	5.4(8)
C18–B–N1–C1	−61.9(8)	C18–B–N1–C4	123.1(6)
C19–B–N1–C1	65.4(8)	C19–B–N1–C4	−109.5(6)
N1–B–N2–C6	−7.1(7)	N1–B–N2–C9	179.6(5)
C18–B–N2–C6	−125.1(6)	C18–B–N2–C9	61.5(8)
C19–B–N2–C6	108.3(6)	C19–B–N2–C9	−65.1(7)
B–N1–C1–C2	−176.5(6)	B–N1–C1–C10	1.3(10)
C4–N1–C1–C2	−0.8(7)	C4–N1–C1–C10	177.0(6)
N1–C1–C2–C3	0.7(8)	N1–C1–C2–C11	−179.1(6)
C10–C1–C2–C3	−177.1(6)	C10–C1–C2–C11	3.1(11)
C1–C2–C3–C4	−0.3(7)	C1–C2–C3–C13	−178.9(7)
C11–C2–C3–C4	179.5(6)	C11–C2–C3–C13	0.9(11)
B–N1–C4–C3	176.3(5)	B–N1–C4–C5	−1.5(9)
C1–N1–C4–C3	0.6(7)	C1–N1–C4–C5	−177.2(5)
C2–C3–C4–N1	−0.2(7)	C2–C3–C4–C5	177.3(6)
C13–C3–C4–N1	178.4(7)	C13–C3–C4–C5	−4.2(12)
N1–C4–C5–C6	−2.1(9)	N1–C4–C5–C20	179.8(5)
C3–C4–C5–C6	−179.3(6)	C3–C4–C5–C20	2.5(10)
C4–C5–C6–N2	0.5(9)	C4–C5–C6–C7	−180.0(6)
C20–C5–C6–N2	178.6(5)	C20–C5–C6–C7	−1.8(10)
B–N2–C6–C5	4.8(8)	B–N2–C6–C7	−174.8(5)
C9–N2–C6–C5	179.3(5)	C9–N2–C6–C7	−0.3(6)
N2–C6–C7–C8	0.2(7)	N2–C6–C7–C14	178.0(6)
C5–C6–C7–C8	−179.4(6)	C5–C6–C7–C14	−1.6(11)
C6–C7–C8–C9	0.0(7)	C6–C7–C8–C15	−179.2(6)
C14–C7–C8–C9	−177.9(6)	C14–C7–C8–C15	2.8(11)
B–N2–C9–C8	174.7(5)	B–N2–C9–C17	−4.7(10)
C6–N2–C9–C8	0.3(7)	C6–N2–C9–C17	−179.1(6)
C7–C8–C9–N2	−0.2(7)	C7–C8–C9–C17	179.2(6)
C15–C8–C9–N2	179.0(6)	C15–C8–C9–C17	−1.6(11)

C1–C2–C11–C12	94.4(8)	C3–C2–C11–C12	−85.4(9)
C7–C8–C15–C16	77.8(9)	C9–C8–C15–C16	−101.3(8)
C4–C5–C20–C21	90.4(7)	C4–C5–C20–C25	−88.4(7)
C6–C5–C20–C21	−87.8(7)	C6–C5–C20–C25	93.4(7)
C5–C20–C21–C22	−178.0(5)	C25–C20–C21–C22	0.7(9)
C20–C21–C22–C23	1.0(9)	C21–C22–C23–C24	−1.2(9)
C21–C22–C23–P2	179.3(4)	C22–C23–C24–C25	−0.2(9)
P2–C23–C24–C25	179.3(5)	C23–C24–C25–C20	1.8(10)
C5–C20–C25–C24	176.6(6)	C21–C20–C25–C24	−2.1(10)
C22–C23–P2–Re	4.1(6)	C22–C23–P2–C39	−122.6(5)
C22–C23–P2–C40	125.6(5)	C24–C23–P2–Re	−175.4(4)
C24–C23–P2–C39	57.9(5)	C24–C23–P2–C40	−53.9(5)
Re–P1–C26–C27	−58.0(5)	Re–P1–C26–C31	124.2(5)
C32–P1–C26–C27	71.2(5)	C32–P1–C26–C31	−106.6(5)
C38–P1–C26–C27	−179.1(5)	C38–P1–C26–C31	3.1(6)
P1–C26–C27–C28	−177.8(5)	C31–C26–C27–C28	0.0(9)
C26–C27–C28–C29	0.3(10)	C27–C28–C29–C30	−0.5(11)
C28–C29–C30–C31	0.5(11)	P1–C26–C31–C30	177.8(5)
C27–C26–C31–C30	0.0(9)	C29–C30–C31–C26	−0.3(11)
Re–P1–C32–C33	50.0(5)	Re–P1–C32–C37	−135.6(4)
C26–P1–C32–C33	−81.6(5)	C26–P1–C32–C37	92.8(5)
C38–P1–C32–C33	168.1(4)	C38–P1–C32–C37	−17.6(5)
P1–C32–C33–C34	173.8(5)	C37–C32–C33–C34	−0.8(9)
C32–C33–C34–C35	1.3(10)	C33–C34–C35–C36	−0.8(10)
C34–C35–C36–C37	−0.1(10)	C35–C36–C37–C32	0.6(10)
P1–C32–C37–C36	−174.5(5)	C33–C32–C37–C36	−0.1(9)
Re–P1–C38–C39	43.5(4)	C26–P1–C38–C39	172.4(4)
C32–P1–C38–C39	−80.5(4)	P1–C38–C39–P2	−48.2(5)
Re–P2–C39–C38	30.5(5)	C23–P2–C39–C38	165.4(4)
C40–P2–C39–C38	−86.5(5)	Re–P2–C40–C41	−46.2(4)
C23–P2–C40–C41	−178.3(4)	C39–P2–C40–C41	72.4(4)
P2–C40–C41–P3	49.2(5)	Re–P3–C41–C40	−29.7(4)

C42–P3–C41–C40	94.2(4)	C48–P3–C41–C40	−160.4(4)
Re–P3–C42–C43	158.1(4)	Re–P3–C42–C47	−27.0(5)
C41–P3–C42–C43	38.4(5)	C41–P3–C42–C47	−146.7(4)
C48–P3–C42–C43	−70.0(5)	C48–P3–C42–C47	105.0(5)
P3–C42–C43–C44	175.8(4)	C47–C42–C43–C44	0.7(8)
C42–C43–C44–C45	−0.2(9)	C43–C44–C45–C46	−0.4(9)
C44–C45–C46–C47	0.3(10)	C45–C46–C47–C42	0.2(9)
P3–C42–C47–C46	−175.8(5)	C43–C42–C47–C46	−0.8(8)
Re–P3–C48–C49	−133.0(4)	Re–P3–C48–C53	52.1(5)
C41–P3–C48–C49	−9.9(5)	C41–P3–C48–C53	175.2(4)
C42–P3–C48–C49	98.8(5)	C42–P3–C48–C53	−76.1(5)
P3–C48–C49–C50	−174.6(5)	C53–C48–C49–C50	0.3(9)
C48–C49–C50–C51	−0.4(9)	C49–C50–C51–C52	0.6(10)
C50–C51–C52–C53	−0.7(9)	P3–C48–C53–C52	174.7(4)
C49–C48–C53–C52	−0.4(8)	C51–C52–C53–C48	0.6(9)
F1–C57–S–O4	60.7(10)	F1–C57–S–O5	−178.1(9)
F1–C57–S–O6	−57.2(10)	F2–C57–S–O4	−55.3(9)
F2–C57–S–O5	65.9(9)	F2–C57–S–O6	−173.3(9)
F3–C57–S–O4	−177.7(12)	F3–C57–S–O5	−56.5(14)
F3–C57–S–O6	64.4(14)	O7–C58–C59–C60	6.2(10)
O7–C58–C59–C64	179.9(7)	O8–C58–C59–C60	−171.9(7)
O8–C58–C59–C64	1.9(10)	C58–C59–C60–C61	171.0(7)
C64–C59–C60–C61	−2.8(11)	C59–C60–C61–C62	0.9(11)
C59–C60–C61–N3	−171.9(7)	C60–C61–C62–C63	1.7(10)
C60–C61–C62–N4	−179.1(6)	N3–C61–C62–C63	175.1(7)
N3–C61–C62–N4	−5.8(10)	C61–C62–C63–C64	−2.5(10)
N4–C62–C63–C64	178.3(6)	C58–C59–C64–C63	−171.9(7)
C60–C59–C64–C63	2.0(11)	C62–C63–C64–C59	0.6(11)
C66'–C65–C66–C67	60(4)	C65–C66–C67–C68	−147(5)
C66–C67–C68–C69	−157(5)	C67–C68–C69–C68'	65(4)
C66–C65–C66'–C67'	−75(4)	C65–C66'–C67'–C68'	145(5)
C68–C69–C68'–C67'	−67(4)	C66'–C67'–C68'–C69	133(6)

Table 5:5. Anisotropic displacement parameters (Å^2) for ljh120041 (**5**). The anisotropic displacement factor exponent takes the form: $-2\pi^2[h^2a^{*2}U^{11} + ... + 2hka^*b^*U^{12}]$

	U^{11}	U^{22}	U^{33}	U^{23}	U^{13}	U^{12}
Re	0.02057(19)	0.01549(19)	0.01942(19)	0.00224(11)	0.00150(12)	−0.00142(11)
B	0.028(3)	0.028(3)	0.022(3)	0.006(2)	−0.004(2)	0.004(2)
N1	0.029(3)	0.033(3)	0.020(2)	0.0013(19)	−0.0029(19)	−0.0017(19)
N2	0.029(3)	0.028(3)	0.020(2)	0.0080(18)	−0.0014(18)	0.0018(19)
C1	0.030(3)	0.036(3)	0.027(3)	0.000(2)	−0.002(2)	−0.002(2)
C2	0.030(3)	0.036(3)	0.029(3)	−0.001(2)	0.006(2)	−0.007(2)
C3	0.036(3)	0.036(3)	0.023(3)	0.005(2)	0.003(2)	−0.008(2)
C4	0.032(3)	0.028(3)	0.017(2)	0.003(2)	0.000(2)	−0.002(2)
C5	0.032(3)	0.021(3)	0.024(3)	0.003(2)	0.000(2)	0.002(2)
C6	0.027(3)	0.026(3)	0.020(3)	0.002(2)	0.000(2)	0.000(2)
C7	0.029(3)	0.032(3)	0.027(3)	0.004(2)	0.006(2)	0.005(2)
C8	0.035(3)	0.033(3)	0.035(3)	0.005(3)	0.011(3)	0.001(2)
C9	0.037(3)	0.033(3)	0.023(3)	0.007(2)	0.008(2)	0.005(2)
C10	0.030(3)	0.054(5)	0.033(3)	0.000(3)	−0.006(3)	−0.002(3)
C11	0.038(4)	0.046(4)	0.037(3)	0.005(3)	0.008(3)	−0.006(3)
C12	0.048(4)	0.066(5)	0.050(4)	0.009(4)	0.016(3)	0.002(4)
C13	0.036(4)	0.067(5)	0.024(3)	0.013(3)	0.002(3)	−0.009(3)
C14	0.026(3)	0.055(4)	0.032(3)	0.006(3)	0.004(2)	0.007(3)
C15	0.040(4)	0.050(4)	0.037(3)	0.008(3)	0.016(3)	0.004(3)
C16	0.041(4)	0.055(5)	0.048(4)	0.006(3)	0.019(3)	−0.002(3)
C17	0.055(4)	0.052(4)	0.026(3)	0.012(3)	0.010(3)	0.005(3)
C18	0.042(4)	0.031(3)	0.035(3)	0.011(3)	0.006(3)	0.008(3)
C19	0.044(4)	0.032(3)	0.024(3)	0.000(2)	−0.002(3)	−0.002(3)
C20	0.027(3)	0.029(3)	0.019(3)	0.004(2)	−0.002(2)	−0.001(2)
C21	0.026(3)	0.021(3)	0.032(3)	0.007(2)	−0.001(2)	0.002(2)
C22	0.030(3)	0.024(3)	0.021(3)	0.001(2)	0.001(2)	−0.001(2)
C23	0.029(3)	0.026(3)	0.019(3)	0.008(2)	0.001(2)	−0.003(2)

C24	0.042(3)	0.022(3)	0.025(3)	0.007(2)	−0.008(2)	−0.002(2)
C25	0.043(4)	0.022(3)	0.026(3)	0.002(2)	−0.008(2)	−0.009(2)
P1	0.0239(7)	0.0204(7)	0.0206(7)	0.0023(5)	0.0038(5)	−0.0023(5)
P2	0.0236(7)	0.0179(7)	0.0181(6)	0.0033(5)	0.0002(5)	−0.0018(5)
P3	0.0202(7)	0.0180(7)	0.0182(6)	0.0015(5)	0.0018(5)	−0.0017(5)
C26	0.025(3)	0.037(3)	0.022(3)	0.001(2)	0.004(2)	−0.003(2)
C27	0.035(3)	0.041(4)	0.028(3)	0.000(3)	0.007(2)	0.003(3)
C28	0.040(4)	0.055(4)	0.027(3)	−0.009(3)	0.005(3)	−0.001(3)
C29	0.046(4)	0.069(5)	0.022(3)	0.005(3)	0.001(3)	0.010(3)
C30	0.055(5)	0.057(5)	0.030(3)	0.011(3)	0.007(3)	0.011(4)
C31	0.038(3)	0.042(4)	0.023(3)	0.008(2)	0.004(2)	0.005(3)
C32	0.026(3)	0.031(3)	0.019(3)	0.004(2)	0.002(2)	−0.001(2)
C33	0.025(3)	0.034(3)	0.035(3)	−0.003(2)	0.003(2)	−0.006(2)
C34	0.036(3)	0.038(4)	0.042(4)	−0.010(3)	0.008(3)	0.006(3)
C35	0.034(3)	0.052(4)	0.040(4)	0.007(3)	0.016(3)	0.009(3)
C36	0.027(3)	0.049(4)	0.036(3)	0.010(3)	0.005(3)	−0.004(3)
C37	0.028(3)	0.030(3)	0.031(3)	0.004(2)	0.005(2)	−0.006(2)
C38	0.035(3)	0.021(3)	0.020(3)	0.003(2)	0.003(2)	−0.005(2)
C39	0.032(3)	0.021(3)	0.023(3)	0.005(2)	0.000(2)	−0.009(2)
C40	0.026(3)	0.028(3)	0.024(3)	0.000(2)	0.001(2)	−0.001(2)
C41	0.026(3)	0.023(3)	0.026(3)	−0.001(2)	0.000(2)	0.003(2)
C42	0.024(3)	0.026(3)	0.017(2)	0.005(2)	0.000(2)	−0.002(2)
C43	0.029(3)	0.032(3)	0.023(3)	0.005(2)	0.006(2)	0.004(2)
C44	0.031(3)	0.043(4)	0.027(3)	0.005(2)	0.007(2)	0.006(3)
C45	0.028(3)	0.051(4)	0.030(3)	0.010(3)	0.008(2)	−0.004(3)
C46	0.035(3)	0.031(3)	0.041(4)	0.008(3)	0.006(3)	−0.008(3)
C47	0.024(3)	0.029(3)	0.033(3)	0.004(2)	0.006(2)	−0.001(2)
C48	0.021(3)	0.023(3)	0.023(3)	0.010(2)	0.003(2)	0.003(2)
C49	0.036(3)	0.023(3)	0.022(3)	0.002(2)	−0.002(2)	0.002(2)
C50	0.040(3)	0.027(3)	0.029(3)	0.010(2)	0.001(2)	0.006(2)
C51	0.038(3)	0.036(3)	0.025(3)	0.009(2)	0.000(2)	0.002(3)
C52	0.031(3)	0.035(3)	0.024(3)	0.001(2)	0.000(2)	0.002(2)

C53	0.031(3)	0.028(3)	0.024(3)	0.001(2)	0.003(2)	0.001(2)
C54	0.031(3)	0.013(2)	0.027(3)	0.003(2)	0.001(2)	0.001(2)
O1	0.043(3)	0.037(3)	0.053(3)	0.006(2)	0.003(2)	0.010(2)
C55	0.034(3)	0.015(3)	0.035(3)	0.005(2)	0.008(3)	−0.001(2)
O2	0.046(3)	0.031(2)	0.037(2)	−0.011(2)	−0.003(2)	−0.002(2)
C56	0.024(3)	0.022(3)	0.037(3)	0.003(2)	−0.005(2)	0.004(2)
O3	0.039(3)	0.056(3)	0.040(3)	0.027(2)	0.009(2)	−0.008(2)
C57	0.047(5)	0.091(8)	0.112(9)	−0.046(7)	0.046(6)	−0.022(5)
F1	0.036(3)	0.239(12)	0.172(9)	−0.065(8)	0.005(4)	−0.005(5)
F2	0.146(7)	0.095(5)	0.102(5)	−0.012(4)	0.083(5)	−0.056(5)
F3	0.116(7)	0.164(9)	0.216(11)	−0.104(8)	0.104(7)	−0.007(6)
S	0.0451(10)	0.0257(8)	0.0596(11)	−0.0036(7)	0.0064(8)	−0.0004(7)
O4	0.071(4)	0.037(3)	0.065(4)	0.002(2)	0.032(3)	−0.005(3)
O5	0.066(4)	0.107(6)	0.072(4)	−0.011(4)	0.004(3)	−0.028(4)
O6	0.188(11)	0.061(5)	0.138(8)	0.026(5)	0.013(7)	0.059(6)
C58	0.064(5)	0.031(4)	0.047(4)	0.014(3)	0.007(4)	−0.006(3)
C59	0.071(5)	0.028(4)	0.046(4)	0.013(3)	0.003(4)	−0.013(3)
C60	0.050(4)	0.034(4)	0.049(4)	0.004(3)	0.001(3)	−0.012(3)
C61	0.062(5)	0.031(4)	0.043(4)	0.001(3)	0.008(3)	−0.017(3)
C62	0.057(4)	0.021(3)	0.052(4)	0.005(3)	−0.006(3)	−0.015(3)
C63	0.057(4)	0.031(4)	0.047(4)	0.009(3)	0.001(3)	−0.007(3)
C64	0.064(5)	0.036(4)	0.044(4)	0.011(3)	0.005(3)	−0.006(3)
O7	0.061(4)	0.065(4)	0.055(3)	0.023(3)	0.001(3)	0.001(3)
O8	0.073(4)	0.058(4)	0.042(3)	0.016(3)	−0.003(3)	0.006(3)
N3	0.073(5)	0.069(5)	0.064(5)	0.005(4)	0.007(4)	−0.004(4)
N4	0.069(4)	0.039(4)	0.042(3)	0.007(3)	−0.003(3)	−0.013(3)
C65	0.220(17)	0.150(17)	0.28(2)	0.010(14)	0.127(16)	0.090(14)
C66	0.199(17)	0.16(2)	0.28(2)	0.010(16)	0.129(17)	0.089(15)
C67	0.192(16)	0.161(19)	0.28(2)	0.014(15)	0.130(16)	0.071(15)
C68	0.194(17)	0.18(2)	0.28(2)	0.019(16)	0.127(17)	0.034(17)
C69	0.189(16)	0.20(2)	0.30(2)	0.012(16)	0.111(16)	0.036(15)
C66'	0.211(19)	0.18(2)	0.28(2)	0.009(16)	0.125(17)	0.051(17)

C67'	0.199(17)	0.17(2)	0.29(2)	0.015(15)	0.126(17)	0.045(15)
C68'	0.189(17)	0.19(2)	0.28(2)	0.015(17)	0.126(17)	0.043(17)

Table 5:6. Hydrogen coordinates and isotropic displacement parameters (Å^2) for ljh120041 (**5**).

	x	y	z	U
H10A	1.0537	0.2125	0.8149	0.061
H10B	0.9864	0.2432	0.8790	0.061
H10C	0.9821	0.1455	0.8595	0.061
H11A	1.0534	0.2605	0.7111	0.049
H11B	0.9761	0.2795	0.6368	0.049
H12A	1.0881	0.1741	0.6108	0.081
H12B	1.0305	0.1168	0.6690	0.081
H12C	0.9531	0.1358	0.5947	0.081
H13A	0.6432	0.2250	0.5888	0.066
H13B	0.7622	0.2863	0.5872	0.066
H13C	0.7537	0.1886	0.5658	0.066
H14A	0.3245	0.1789	0.7045	0.056
H14B	0.2459	0.1134	0.7465	0.056
H14C	0.2630	0.2109	0.7710	0.056
H15A	0.2433	0.1606	0.9020	0.050
H15B	0.3193	0.1275	0.9703	0.050
H16A	0.1610	0.0234	0.9227	0.072
H16B	0.2037	0.0234	0.8434	0.072
H16C	0.2797	−0.0097	0.9118	0.072
H17A	0.4883	0.1098	1.0158	0.066
H17B	0.5973	0.0725	0.9913	0.066
H17C	0.6084	0.1700	1.0145	0.066
H18A	0.8571	0.0898	0.9144	0.053
H18B	0.7413	0.0684	0.9513	0.053
H18C	0.7437	0.0357	0.8677	0.053
H19A	0.8565	0.2602	0.9412	0.052
H19B	0.7443	0.2987	0.9079	0.052
H19C	0.7397	0.2430	0.9776	0.052

H21	0.4720	0.0758	0.6242	0.032
H22	0.3597	0.0962	0.5128	0.031
H24	0.4023	0.3444	0.5673	0.038
H25	0.5084	0.3246	0.6812	0.039
H27	0.1934	0.1053	0.1461	0.042
H28	0.1099	0.1190	0.0252	0.050
H29	0.0982	0.2538	−0.0163	0.055
H30	0.1670	0.3701	0.0641	0.056
H31	0.2510	0.3570	0.1855	0.041
H33	0.3656	0.0599	0.2284	0.040
H34	0.5304	0.0158	0.1909	0.046
H35	0.7009	0.1108	0.1948	0.049
H36	0.7040	0.2503	0.2335	0.046
H37	0.5384	0.2957	0.2680	0.037
H38A	0.4019	0.3516	0.2932	0.031
H38B	0.2723	0.3495	0.3120	0.031
H39A	0.3920	0.3699	0.4252	0.032
H39B	0.4626	0.2970	0.4043	0.032
H40A	0.1313	0.2609	0.5064	0.032
H40B	0.2119	0.3482	0.5006	0.032
H41A	0.1287	0.3582	0.3795	0.031
H41B	0.0227	0.3350	0.4251	0.031
H43	−0.1555	0.2834	0.3997	0.033
H44	−0.3343	0.2243	0.4298	0.040
H45	−0.3875	0.0801	0.4227	0.044
H46	−0.2614	−0.0064	0.3839	0.044
H47	−0.0821	0.0522	0.3536	0.035
H49	−0.0043	0.3819	0.2927	0.033
H50	−0.0907	0.4259	0.1815	0.038
H51	−0.1532	0.3311	0.0795	0.040
H52	−0.1335	0.1913	0.0889	0.037
H53	−0.0472	0.1457	0.1994	0.033
H60	0.0506	0.5572	0.2281	0.056

H63	0.4382	0.5229	0.2386	0.056
H64	0.3234	0.5275	0.1249	0.059
H8	0.0898	0.5126	−0.0019	0.071
H3A	0.0714	0.5443	0.3627	0.085
H3B	0.1908	0.5409	0.4057	0.085
H4A	0.4512	0.5217	0.3710	0.063
H4B	0.3474	0.5314	0.4106	0.063
H65A	0.5951	0.3473	1.1080	0.302
H65B	0.4684	0.3741	1.0992	0.302
H65C	0.4875	0.2873	1.0609	0.302
H65D	0.5867	0.3195	1.0980	0.302
H65E	0.5373	0.4057	1.0880	0.302
H65F	0.4499	0.3211	1.0913	0.302
H66A	0.5985	0.3583	0.9830	0.237
H66B	0.5793	0.4457	1.0216	0.237
H67A	0.3644	0.3282	0.9778	0.238
H67B	0.3778	0.4265	0.9748	0.238
H68A	0.4459	0.4002	0.8457	0.249
H68B	0.3805	0.3068	0.8500	0.249
H69A	0.2553	0.3879	0.7935	0.335
H69B	0.2152	0.3586	0.8695	0.335
H69C	0.2802	0.4516	0.8652	0.335
H69D	0.2589	0.4168	0.7996	0.335
H69E	0.2685	0.3323	0.8399	0.335
H69F	0.2194	0.4070	0.8794	0.335
H66C	0.4965	0.2711	0.9813	0.253
H66D	0.5843	0.3563	0.9780	0.253
H67C	0.3912	0.4205	1.0001	0.249
H67D	0.3302	0.3354	0.9567	0.249
H68C	0.3997	0.4882	0.8906	0.251
H68D	0.4493	0.4127	0.8508	0.251

6 Absorption and Emission Spectroscopy

6.1 General Considerations

Absorption spectra were recorded with a Hitachi Model U-3310 spectrophotometer while fluorescence studies were recorded with a Hitachi F-4500 fluorescence spectrophotometer. Solvents used for spectroscopy experiments were spectrophotometric grade. Absorption and emission spectra were recorded for all compounds in dry degassed tetrahydrofuran at room temperature. Fluorescence quantum yields were measured with respect to 4,4-difluoro-8-phenyl-1,3,5,7-tetramethyl -2,6-diethyl-4-bora-3a,4a-diaza-s-indacene (Φ = 0.76, λ_{abs} = 524 nm, λ_{em} = 537 nm, ε = 86,000 $M^{-1}cm^{-1}$, THF).[1] Dyes were excited at 485 nm and excitation and emission slits were both set to 5 nm.

6.2 Absorption and Emission Spectra

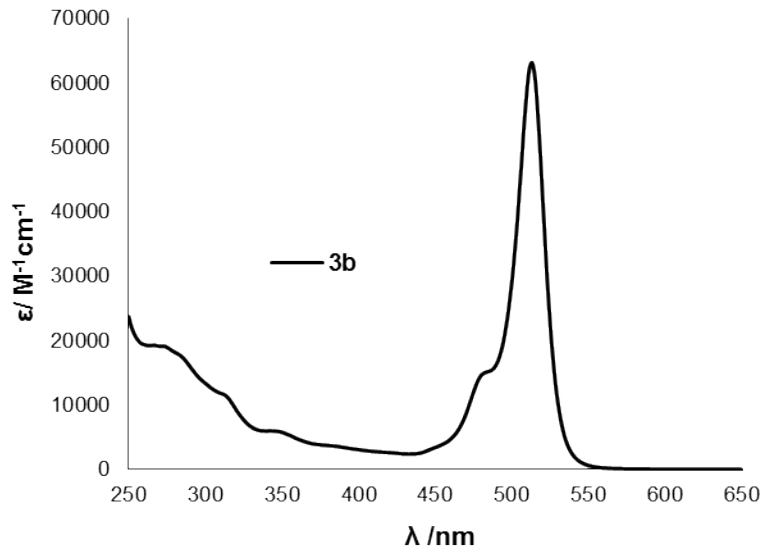

Fig. 6.1. Absorption spectrum of **3b** in THF at room temperature.

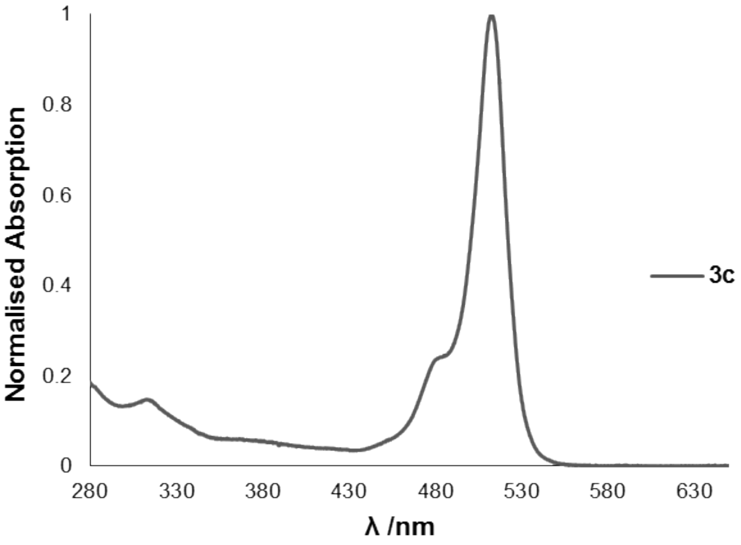

Fig. 6.2. Absorption spectrum of **3c** in THF at room temperature.

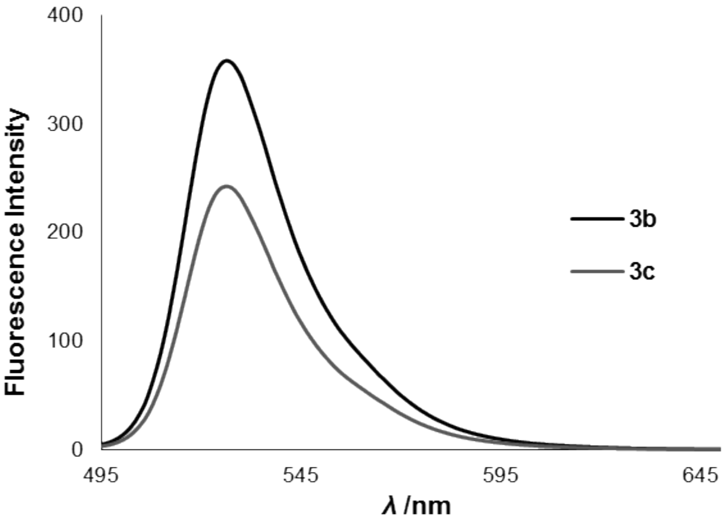

Fig. 6.3. Emission spectra of **3b** and **3c** in THF at room temperature (excitation λ = 485 nm).

6.3 Quantum Yield Method[11]

The fluorescence quantum yield (Φ_F) can be defined as the ratio of emitted photons relative to the number of absorbed photons. All quantum yields were measured in solution at room temperature. In order to determine the fluorescence quantum yield an appropriate reference compound was selected (see General Considerations), which absorbs and emits over ranges comparable to those of the studied sample. The excitation wavelength, slit widths and the

emission range were kept constant for both the reference and the sample. Optically dilute solutions were prepared to possess exactly the same absorbance at the excitation wavelength (A < 0.09 at the excitation wavelength, with an error limit of ± 0.005 between the reference and sample). The following formulae were used to correct the relative emission areas for minor differences in absorbance:

$$A = \log\left(\frac{I_0}{I_T}\right)$$

$$I_a + I_T = I_0 = 1$$

$$QY_{REL} = \frac{area}{I_a}$$

A is the absorbance, I_0 is the intensity of incident light, I_T is the intensity of the transmitted light, I_a is the intensity of absorbed light and QY_{REL} is the relative quantum yield.

Where necessary the quantum yields were corrected for the differences in the refractive index of the solvent:

$$QY_A = \frac{\eta_A^2}{\eta_B^2} QY_B$$

The subscripts A and B denote two difference solvents and η is the refractive index.

The quantum yield of the sample can now be determined using the following equation:

$$QY^{Sl} = \frac{QY_{\mathrm{Re}l}^{Sl}}{QY_{\mathrm{Re}l}^{\mathrm{Re}f}} QY^{\mathrm{Re}f}$$

Superscript Sl and Ref denote sample and reference respectively.

7 References

1. (a) A. Coskun and E. U. Akkaya, *J. Am. Chem. Soc.*, 2005, **127**, 10464-10465; (b) Y. Gabe, Y. Urano, K. Kikuchi, H. Kojima and T. Nagano, *J. Am. Chem. Soc.*, 2004, **126**, 3357.
2. L. E. Crascall, J. L. Spencer, R. A. Doyle and R. J. Angelici, *Inorg. Synth.*, 1990, **28**, 346.
3. J. Chatt, J. R. Dilworth, H. P. Gunz and G. J. Leigh, *J. Organomet. Chem.*, 1974, **64**, 245.
4. L. H. Davies, R. W. Harrington, W. Clegg and L. J. Higham, manuscript in preparation.
5. S. P. Schmidt, J. Nitschke, W. C. Trogler, S. I. Huckett and R. J. Angelici, *Inorg. Synth.*, 1989, **26**, 113.
6. L. H. Davies, B. Stewart, R. W. Harrington, W. Clegg and L. J. Higham, *Angew. Chem. Int. Ed.*, 2012, **51**, 4921.
7. H. Jiang, B. B. Kasten, H. Liu, S. Qi, Y. Liu, M. Tian, C. L. Barnes, H. Zhang, Z. Cheng and P. D. Benny, *Bioconjugate Chem.*, 2012, **23**, 2300.
8. *CrysAlisPro*, Agilent Technologies Ltd., Oxford, 2008–2013.
9. *SHELXTL*, Bruker AXS Inc., Madison, Wisconsin, 2008–2013; *SHELXL-2013*, G. M. Sheldrick, University of Göttingen, Germany, 2013.
10. A. L. Spek, *Acta Crystallogr. Sect. D*, 2009, **65**, 148.
11. J. R. Lakowicz, *Principles of Fluorescence Spectroscopy*, 3rd ed., Springer, New York, **2006**.
